# Neuroanatomy of the equine brain as revealed by high-field (3Tesla) magnetic-resonance-imaging

**DOI:** 10.1371/journal.pone.0213814

**Published:** 2019-04-01

**Authors:** Martin J. Schmidt, Carola Knemeyer, Helmut Heinsen

**Affiliations:** 1 Small Animal Clinic–Neurosurgery, Neuroradiology and Clinical Neurology, Justus Liebig-University, Giessen, Germany; 2 Department of Psychiatry, Psychosomatics and Psychotherapy, Mental Health Center, University Hospital, Wuerzburg, Germany; 3 Department of Pathology, University of São Paulo Medical School, São Paulo, Brazil; University College London, UNITED KINGDOM

## Abstract

In this study, the morphology of the horse brain (*Equus caballus*) is decribed in detail using high field MRI. The study includes sagittal, dorsal, and transverse T2-weighted images at 0.25 mm resolution at 3 Tesla and 3D models of the brain presenting the external morphology of the brain. Representative gallocyanin stained histological slides of the same brain are presented. The images represent a useful tool for MR image interpretation in horses and may serve as a starting point for further research aiming at in vivo analysis in this species.

## Introduction

Neuroimaging is increasingly important in veterinary large animal neurology. Magnetic resonance imaging (MRI) is more and more used to evaluate intracranial diseases in horses with neurological signs [1–12]. High magnetic field strengths (3 Tesla) are now available in veterinary medicine. Images collected at this field strength provide improvements in image clarity and detail. Despite considerable progress in the technical adaptions of scanners and detection coils to the practical requirements in equine medicine, the description of brain morphology in the horse has been somewhat neglected. Currently, studies describing equine brain anatomy are only available at reduced resolution and from the brain of foals [13,14]. As the number of MRI-investigations of the equine brain grows, so does the need for detailed information about brain structures and special characters of the equine brain, as existing for a number of domestic species [15–19]. The aim of this study was therefore to examine the brain of the horse using high field MRI and to describe the morphology of the equine brain as shown in these images.

## Materials and methods

### Animals

The head of an eight years old warm blood horse was examined post-mortem. The animal was euthanized due to a complicated tarsal fracture. The horse was sedated with 0.4 mg/kg xylazin (Xylazin 2%, CP-Pharma GmbH, Burgdorf, Germany) injected in an intraveneous catheter in the jugular vein. General anesthesia was induced with 0.1 mg/kg diazepam (Diazepam AbZ) and 2.2 mg/kg ketamine (Narketan 10). Euthanasia was performed using 0.12ml/kgkg embutramid (T61). No neurological findings were observed at previous clinical examination. Directly after euthanasia the head was dissected between fourth and fifth cervical vertebrae and was trimmed to fit into a standard human knee coil. MRI was performed 90 minutes after death of the animal. Owner consent was obtained for use of the head in scientific research. All data was anonymized.

### MR imaging

MRI scans were performed on a 3-Tesla Magnetom Verio scanner (Siemens Healthcare, Erlangen, Germany) using an eight channel phased array human knee coil. Anatomical images of the entire brain were acquired in transverse, sagittal and dorsal planes, using a T2-weighted spin echo sequence. To achieve a sufficient signal-to-noise ratio (SNR), 32 averages were accumulated obtaining slices of 3 mm thickness with 672x672mm^2^ field of view (FOV) at 512 x 512 matrix size resulting in 0.25 mm in-plane resolution and 1mm slice distance. Optimum contrast was obtained with 8500 milliseconds (ms) repetition time (TR) and while the echotime (TE) was adjusted to 12 ms. Image acquisition was anisotropic, which is why sagittal and horizontal planes were not recalculated but obtained in consecutive scan sessions. Total scan time was 4 hours and 55 minutes.

### Image processing

Images were reviewed using AMIRA (Mercury Computer Systems) graphical software. This program allows interactive assessment of morphology in all image planes. A 3D model of the outer brain surface was generated based on free hand segmentation of the brain outlines in transverse MR-images. Image segmentation in this context describes the manual tracing of the brain surface. All voxels corresponding to a single anatomical structure in the images are selected and assigned to the same value in the mask. The final mask thus contains information about all selected anatomical structures and, in combination with the original data and polygonal surface reconstruction algorithms, allows the identification of sulci and gyri in 2 D images in association with the produced 3D model. Anatomic structures including the sulci and gyri were identified using a published atlas [20] and by comparison with histological slices produced from the examined brain. Morphological substructures were labelled in transverse images and thereafter located in dorsal and sagittal images using the four-viewer mode of the software. Signal intensities are described in relation to the cortical grey matter as hyperintense (brighter signal compared to cerebral cortex) or hypointense (darker signal as the cerebral cortex).

## Histological preparation

Histological slides in transverse orientation were obtained to support image analysis. After scanning, the brain was removed and fixed by immersion in 10% formalin for 2 weeks. Due to the large size of the brain, the brainstem with the cerebellum was severed at the level of the rostral pons and the hemispheres were divided. Brain tissue was dehydrated in 96% alcohol and embedded with 8% celloidin (180 g of commercial alcohol-moistened celloidin dissolved in a mixture of 750 ml 100% ethanol and 750 ml diethyl ether) for 48 h. The brain together with an excess of 8% celloidin were placed into a big transparent PVC embedding form. The celloidin was concentrated to 16% in a dessicator by a slight vacuum (150 millibar). The 16% celloidin was finally hardened by chloroform vapors and one day prior to cutting with 70% ethanol. The celloidin blocks were serially sectioned on a sliding microtome (Polycut, Leica Mikrosysteme Vertrieb GmbH Wetzlar, Germany) with section thickness of 350 μm. Celloidin served as a support, guiding the microtome knife through the tissue block and preventing tangentially cut gyri from floating away during subsequent staining procedures. Slicing of the histology slides was performed perpendicular to the brainstem axis to match the same orientation as the MR transverse image slices. The slices were then stained free-floating in gallocyanin-chromalum [21] quenched between two filter papers and two perforated stainless steel plates to prevent distortion of the single slices during dehydration in ethanol, ispopropanol:xylene 1:1, and finally xylene. The sections were coverslipped with Permount.

## Results

**Fig 1:**
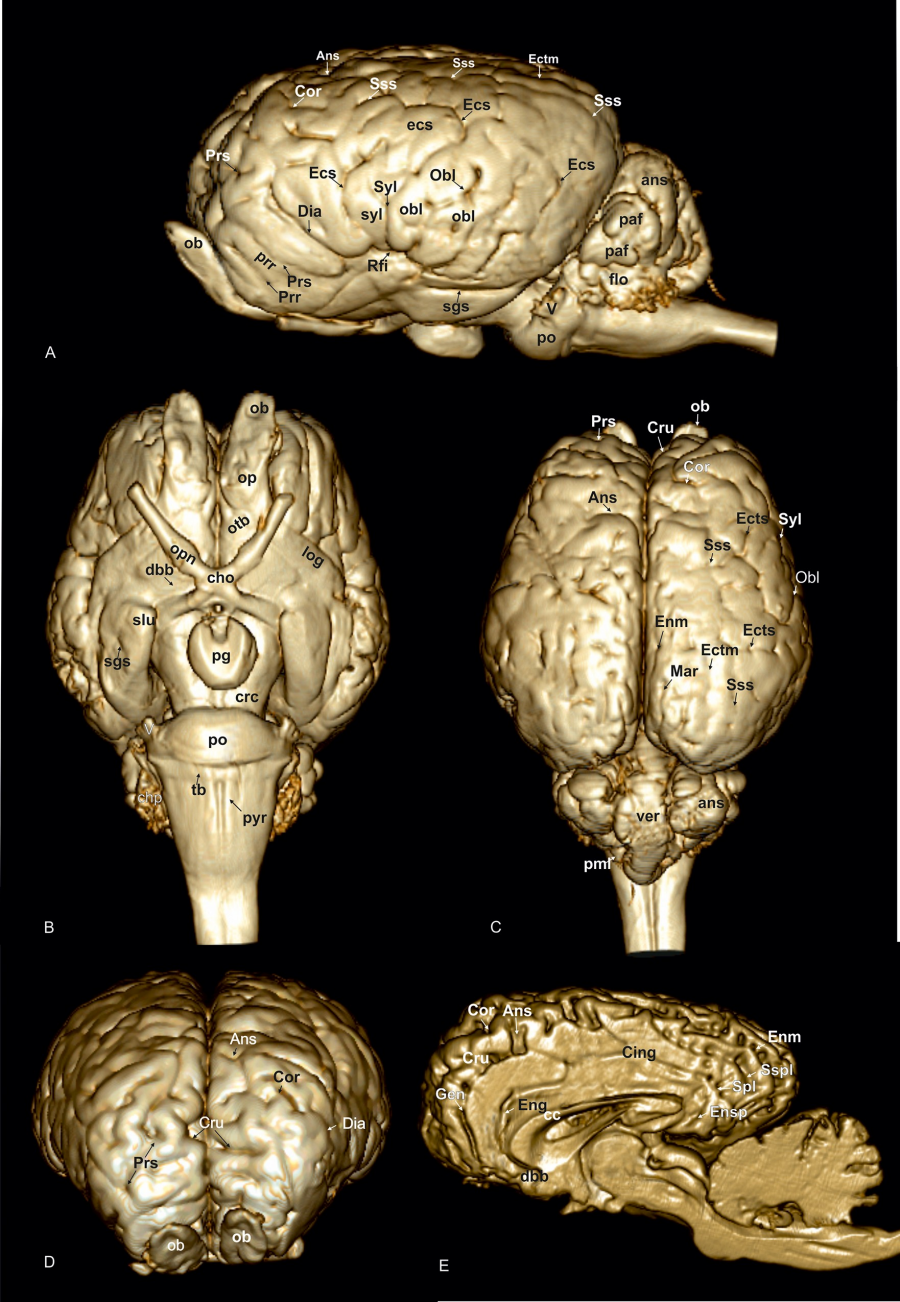
3D model of the equine brain based on magnetic resonace images. (A) Lateral view: Ans: ansate sulcus, ans: ansiform lobule, Cor: coronal sulcus, Dia: diagonal sulcus, Ecs: ectosylvian sulcus, ecs: ectosylvian gyrus, Ectm: ectomarginal sulcus, enrh: endorhinal sulcus, flo: flocculus, ob: olfactory bulb, Obl: oblique sulcus, obl: oblique gyrus, paf: paraflocculus, po: pons, Prr: prorean sulcus, prr: prorean gyrus, Prs: presylvian sulcus, Rfi: rhinal fissure, Sgs: sagittal sulcus, Sss: suprasylvian sulcus, Syl: sylvian fissure, syl: sylvian gyrus, V: trigeminal nerve. (B) Ventral view: cho: optic chiasma, chp: choroid plexus, crc: cerebral crus, dbb: diagonal band of broca, log: lateral olfactory gyrus, ob: olfactory bulb, op: olfactory peduncle, opn: optic nerve, otb: olfactory tubercle, pg: pituitary gland, po: pons, pyr: pyramidal tract, Sgs: sagittal sulcus, slu: semilunar gyrus, tb: trapezoid body. (C) Dorsal view, ans: ansiform lobule, Ans: ansate sulcus, Cor: coronal sulcus, Cru: cruciate sulcus, Ecs: ectosylvian sulcus, Ectm: ectomarginal sulcus, Enm: endomarginal sulcus, Mar: marginal sulcus, ob: olfactory bulb, Obl: oblique sulcus, pml: paramedian lobule, Prs: presylvian sulcus, Sss: suprasylvian sulcus, Syl: sylvian fissure, ver: vermis. (D) Frontal view, Ans: ansate sulcus, Cor: coronal sulcus, Cru: cruciate sulcus, Dia: diagonal sulcus, ob: olfactory bulb, Prs: presylvian sulcus. (E) Midsagittal view, Ans: ansate sulcus, cc: corpus callosum, Cing: cingulate sulcus, Cor: coronal sulcus, Cru: cruciate sulcus, dbb: diagonal band of broca, Eng: endogenual sulcus, Enm: endomarginal sulcus, Ensp: endosplenial sulcus, Gen: genual sulcus, Spl: splenial sulcus, Sspl: suprasplenial sulcus.

**Fig 2:**
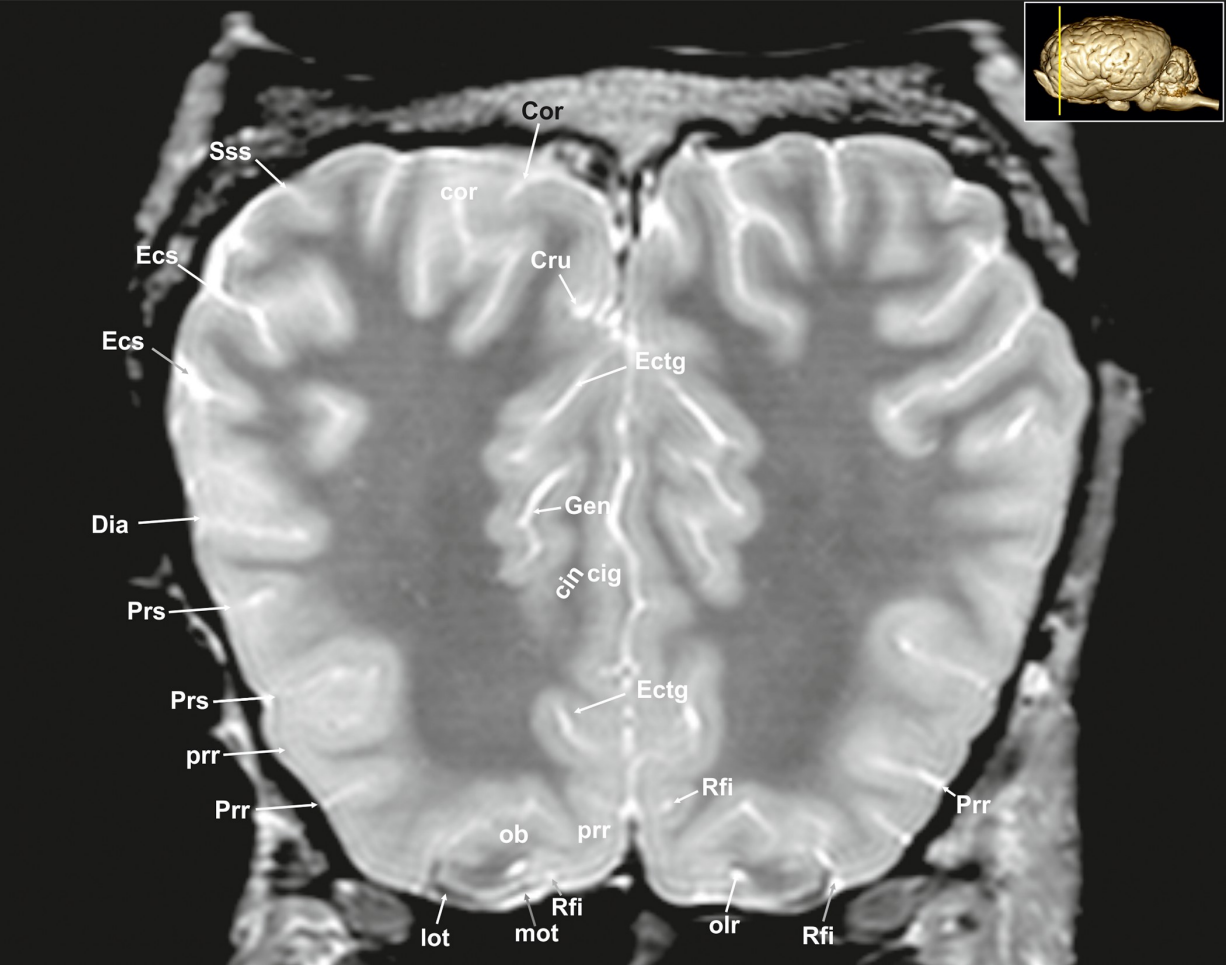
Transverse magnetic resonance image of the equine brain on the level of the olfactory peduncle. cin: cingulum, cig: cingulate gyrus, Cor: coronal sulcus, Cru: cruciate sulcus, Dia: diagonal sulcus, Ecs: ectosylvian sulcus, Ectg: ectogenual sulcus, Gen: genual sulcus, lot: lateral olfactory tract, mot: medial olfactory tract, olr: olfactory recess, Prr: prorean sulcus, prr: prorean gyrus, Prs: presylvian sulcus, Rfi: rhinal fissure, Sss: suprasylvian sulcus.

**Fig 3:**
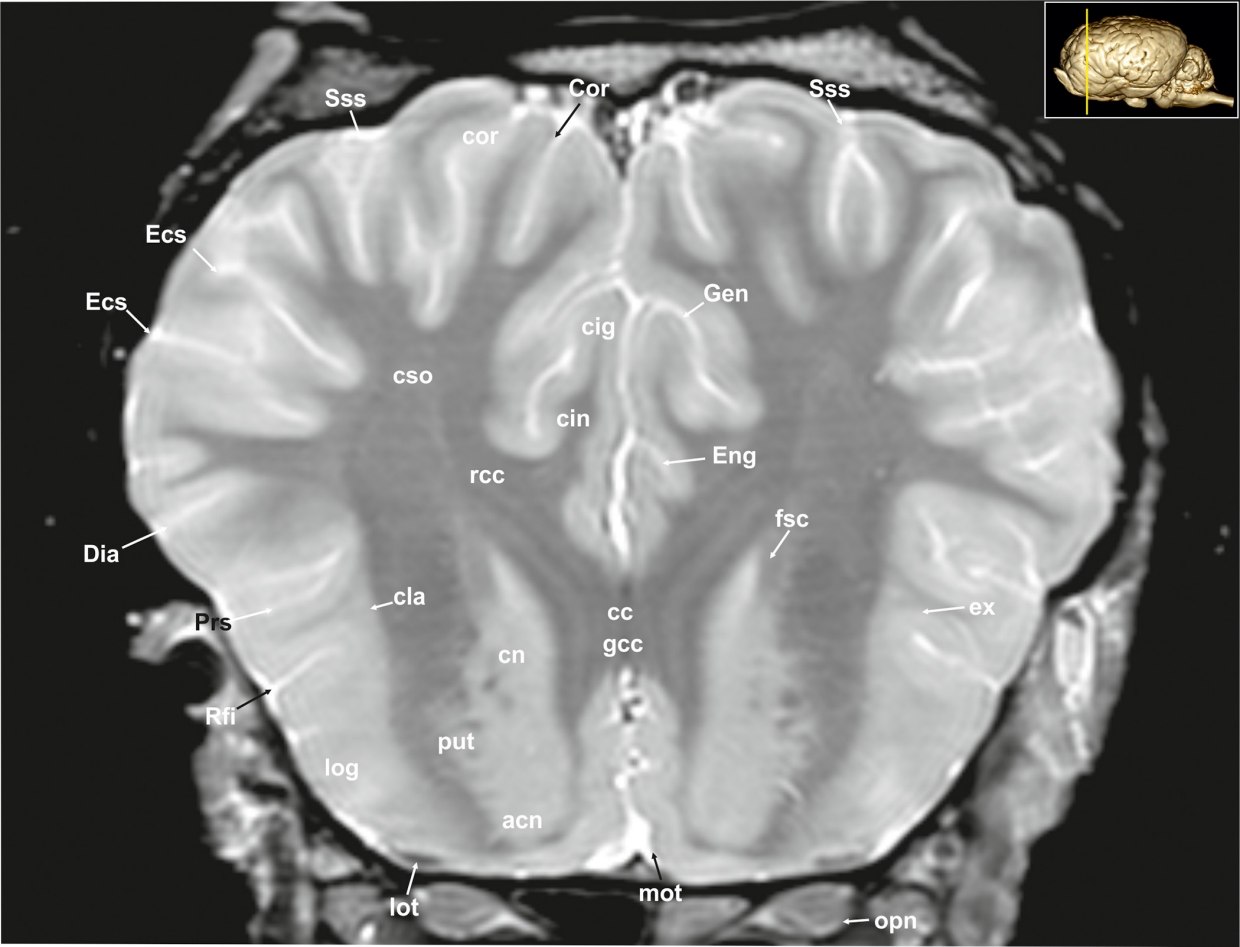
Transverse magnetic resonance image of the equine brain at the level of the genu of the corpus callosum. acn: accumbens nucleus, cin: cingulum, cig: cingulate gyrus, cla: claustrum, cn: caudate nucleus (caput), Cor: coronal sulcus, cor: coronal gyrus, cso: centrum semiovale, Dia: diagonal sulcus, Ecs: ectosylvian sulcus, Eng: endogenual sulcus, ex: extreme capsule, fsc: subcallosal fasciculus, gcc: genu of the corpus callosum, Gen: genual sulcus, log: lateral olfactory gyrus, lot: lateral olfactory tract, mot: medial olfactory tract, opn: optic nerve, Prs: presylvian sulcus, put: putamen, rcc: radiation of corpus callosum, Rfi: rhinal fissure, Sss: suprasylvian sulcus.

**Fig 4:**
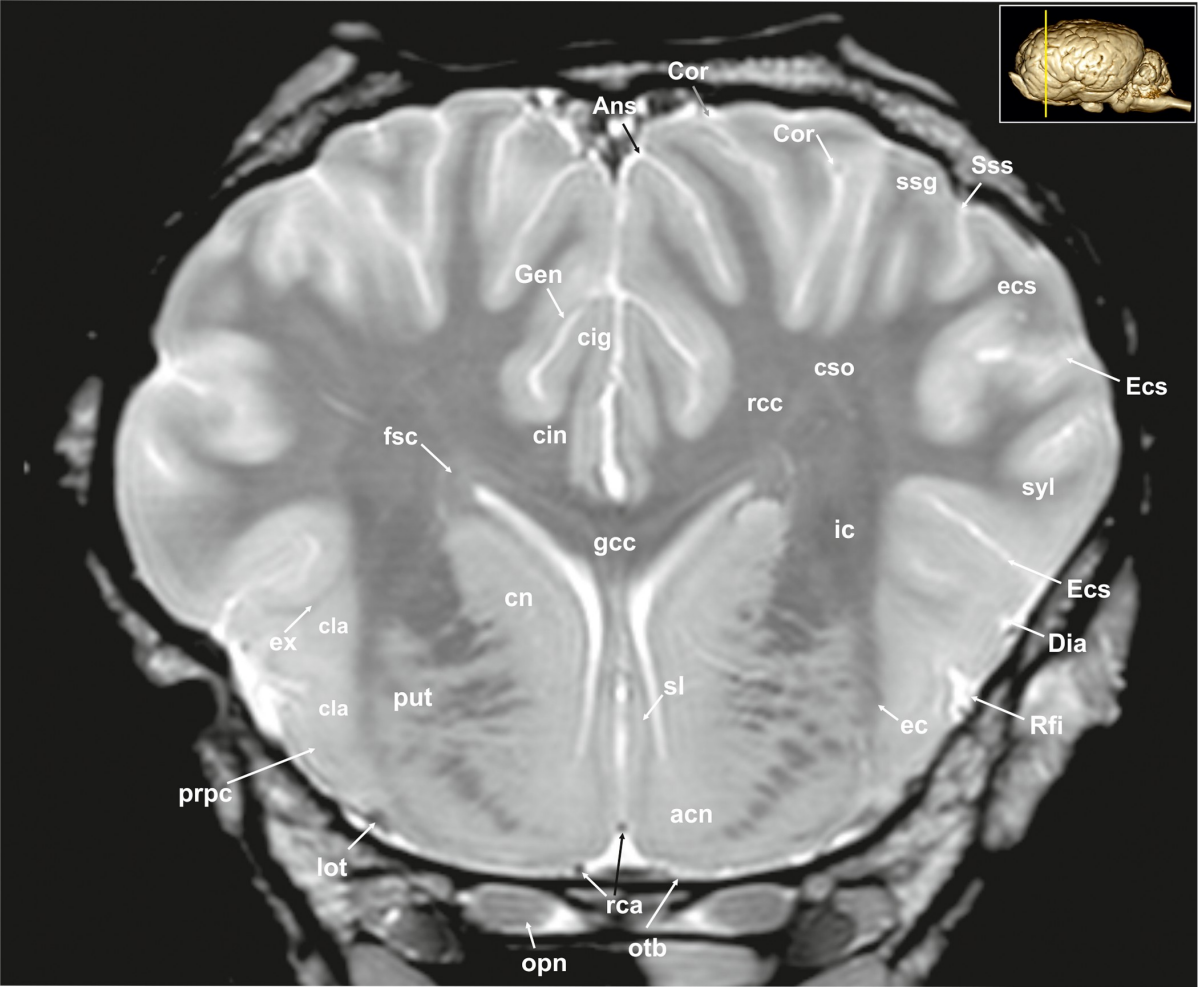
Transverse magnetic resonance image of the equine brain on the level of the septal nuclei. acn: accumbens nucleus, Ans: ansate sulcus, cig: cingulate gyrus, cin: cingulum, cla: claustrum, cn: caudate nucleus (caput), Cor: coronal sulcus, cso: centrum semiovale, Dia: diagonal sulcus, ec: external capsule, ecs: ectosylvian gyrus, Ecs: ectosylvian sulcus, ex: extreme capsule, fsc: subcallosal fasciculus, gcc: genu of the corpus callosum, Gen: genual sulcus, ic: internal capsule, lot: lateral olfactory tract, opn: optic nerve, otb: olfactory tubercle, put: putamen, rca: rostral cerebral artery, rcc: radiation of the corpus callosum, Rfi: rhinal fissure, sl: lateral septal nuclei, ssg: suprasylvian gyrus, Sss: suprasylvian sulcus, syl: sylvian gyrus.

**Fig 5:**
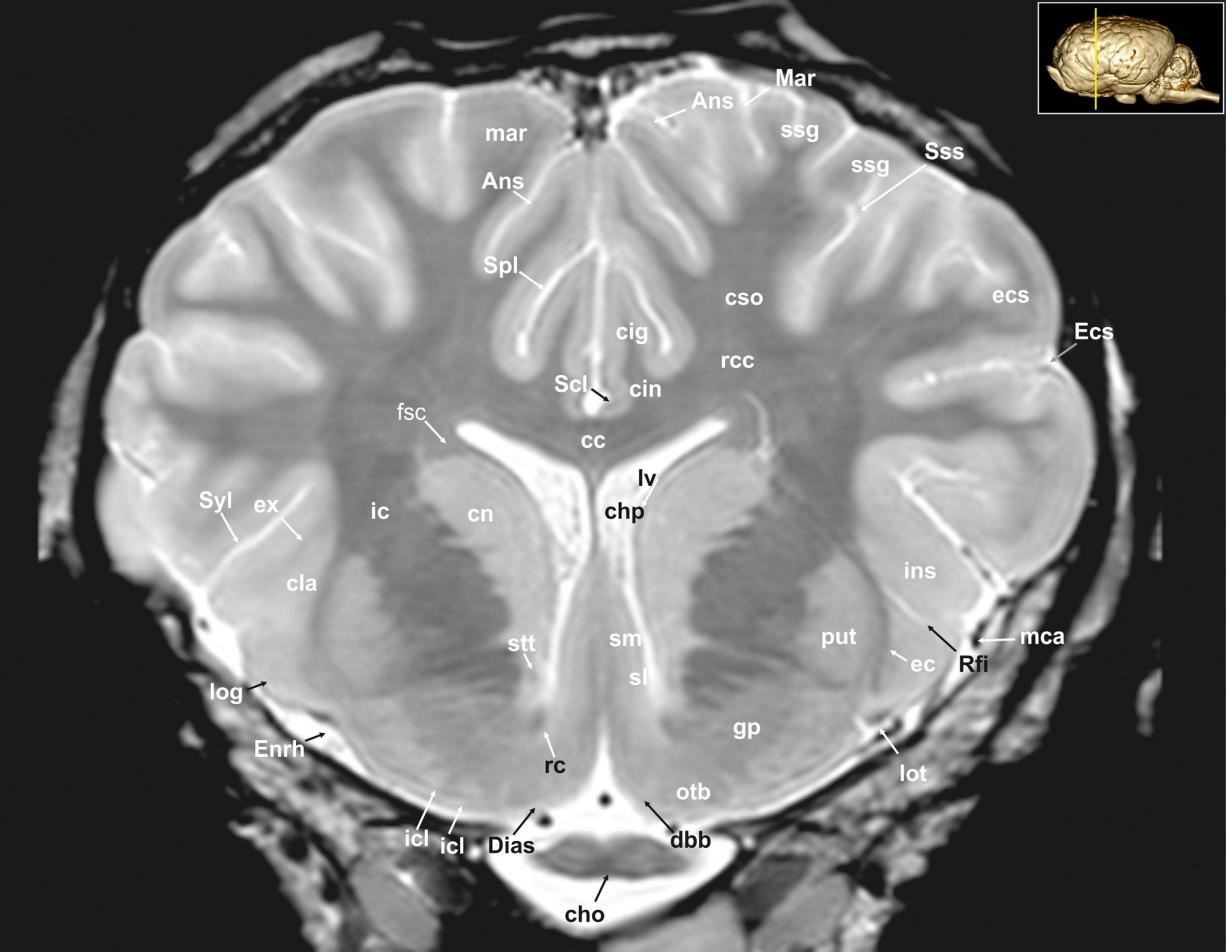
Transverse magnetic resonance image of the equine brain on the level of the diagonal band of Broca. Ans: ansate sulcus, cc: corpus callosum, cho: optic chiasm, chp: choroid plexus, cin: cingulum, cig: cingulate sulcus, cla: claustrum, cn: caudate nucleus, cso: centrum semiovale, dbb: diagonal band of broca, Dias: diagonal sulcus (Rhinencephalon), ec: external capsule, ecs: ectosylvian gyrus, Ecs: ectosylvian sulcus, Enrh: endorhinal sulcus, ex: extreme capsule, fsc: subcallosal fasciculus, gp: globus pallidus, ic: internal capsule, icl: islands of Calleja, ins: insular cortex, log: lateral olfactory gyrus, lot: lateral olfactory tract, lv: lateral ventricle, mar: marginal gyrus, Mar: marginal sulcus, mca: medial cerebral artery, otb: olfactory tubercle, put: putamen, rc: rostral commissure, rcc: radiation of corpus callosum, Rfi: rhinal fissure, Scl: sulcus of corpus callosum, sl: lateral septal nuclei, sm: medial septal nuclei, Spl: splenial sulcus, ssg: suprasylvian gyrus, Sss: suprasylvian sulcus, Syl: Sylvian fissure.

**Fig 6:**
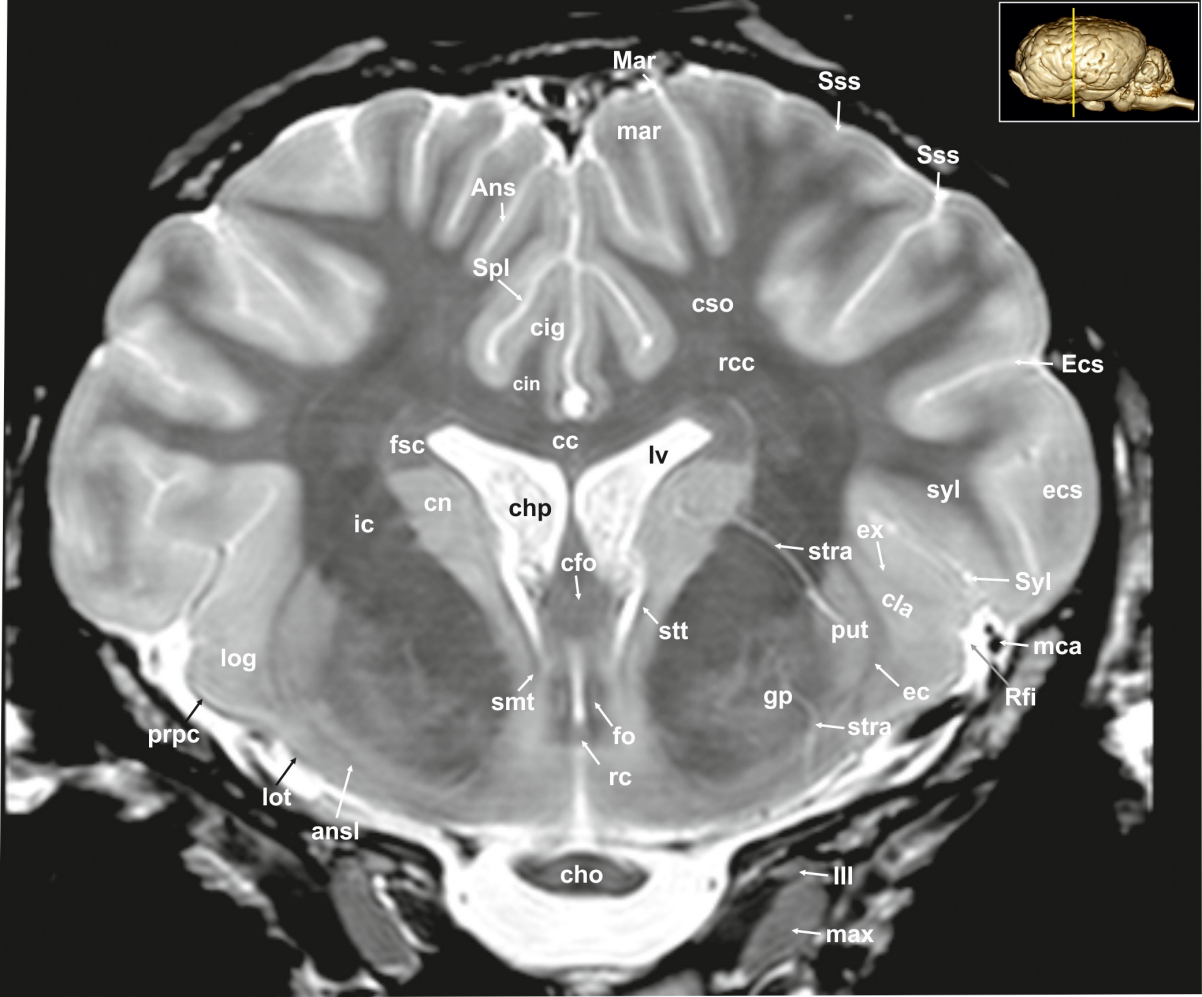
Transverse magnetic resonance image of the equine brain on the level of the rostral commissure. Ans: ansate sulcus, ansl: ansa lenticularis, cc: corpus callosum, cfo: corpus of fornix, cho: optic chiasm, chp: choroid plexus, cig: cingulate gyrus, cin: cingulum, cla: claustrum, cn: caudate nucleus, cso: centrum semiovale, ec: external capsule, Ecs: ectosylvian sulcus, ecs: esctosylvian gyrus, ex: extreme capsule, fo: fornix, fsc: subcallosal fasciculus, gp: globus pallidus, ic: internal capsule, log: lateral olfactory gyrus, lot: lateral olfactory tract, lv: lateral ventricle, Mar: Marginal sulcus, mar: marginal gyrus, max: maxillary nerve, mca: medial cerebral artery, prpc: prepiriform cortex, put: putamen, rc: rostral commissure, rcc: radiation of corpus callosum, Rfi: rhinal fissure, smt: stria medullaris thalami, Spl: splenial sulcus, Sss: suprasylvian sulcus, stra: striate artery, stt: terminal stria, Syl: sylvian fissure, syl: sylvian sulcus, III: oculomotor nerve.

**Fig 7:**
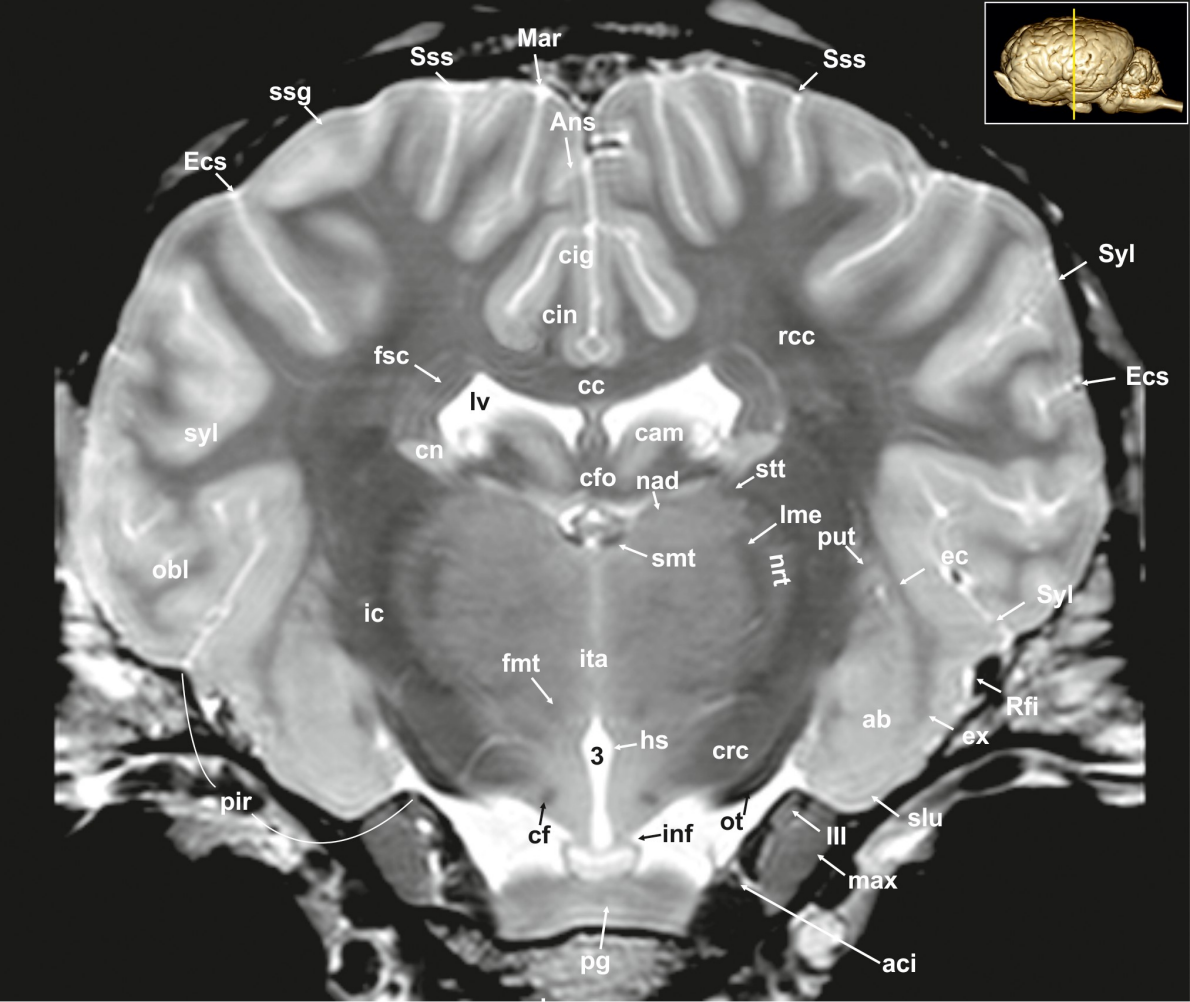
Transverse magnetic resonance image of the equine brain on the level of the amygdala. ab: amygdaloid body, aci: internal carotid artery, Ans: ansate sulcus, cc: corpus callosum, cig: cingulate gyrus, cin: cingulum, cf: column of fornix, cfo: corpus of fornix, cn: caudate nucleus (tail), crc: cerebral crus, ec: external capsule, Ecs: ectosylvian sulcus, ex: extreme capsule, fmt: mammilo-thalamic fasciculus, fsc: subcallosal fasciculus, hs: hypothalamic sulcus, ic: internal capsule, inf: infundibular stalk, ita: interthalamic adhesion, lme: external medullary lamina, lv: lateral ventricle, Mar: marginal sulcus, max: maxillary nerve, nad: nucleus anterior dorsalis thalami, nrt: reticular nucleus of the thalamus, obl: oblique gyrus, ot: optic tract, pg: pituitary gland, put: putamen, rcc: radition of corpus callosum, Rfi: rhinal fissure, slu: gyrus semilunaris, smt: stria medullaris thalami, ssg: suprasylvian gyrus, Sss: suprasylvian sulcus, Syl: sylvian fissure, syl: sylvian gyrus, III: oculomotor nerve, 3: third ventricle.

**Fig 8:**
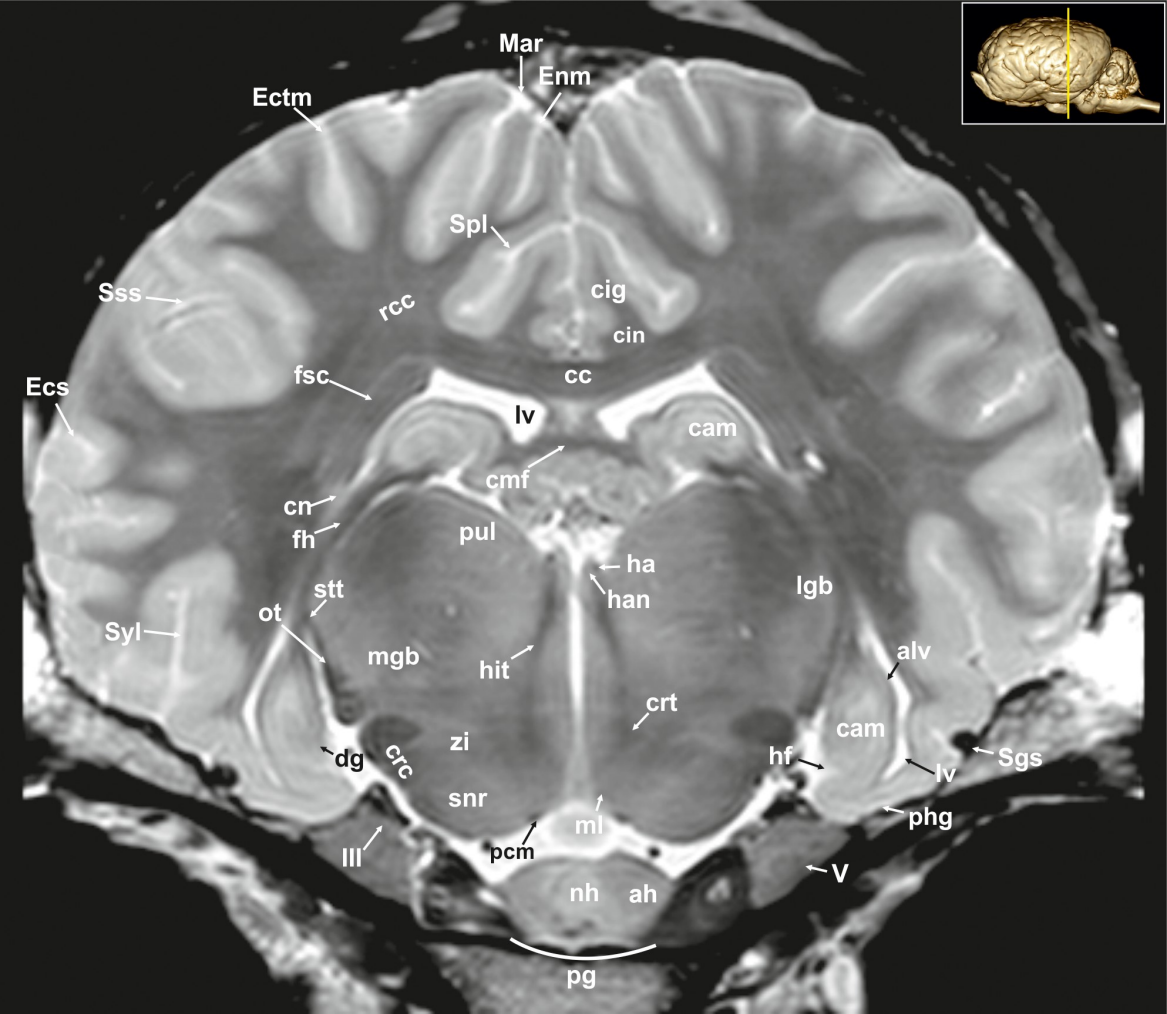
Transverse magnetic resonance image of the equine on the level of the hippocampus. ah: adenohypophysis, alv: alveus, cam: ammon’s horn, cc: corpus callosum, cin: cingulum, cmf: commissure of fornix, cn: caudate nucleus, crc: cerebral crus, crt: rubro-cerebello-thalamic tract, dg: dentate gyrus, Ecs: ectosylvian sulcus, Ectm: ectomarginal sulcus, Enm: endomarginal sulcus, fh: fimbria of the hippocampus, fsc: subcallosal fasciculus, ha: habenula, han: habenular nuclei, hf: hippocampal fissure, hit: habenulo-interpeduncular tract, lgb: lateral geniculate body, lv: lateral ventricle, Mar: marginal sulcus, max: maxillary nerve, mgb: medial geniculate body, ml: medial lemniscus, nh: neurohypophysis, ot: optic tract, pcm: peduncles of the mammillary body, phg: parahippocampal gyrus, pul: pulvinar nuclei, rcc: radiation of corpus callosum, Sgs: sagittal sulcus, snr: substantia nigra, Sss: suprasylvian sulcus, stt: terminal stria; Syl: sylvian fissure, zi: zona incerta, III: oculomotor nerve.

**Fig 9:**
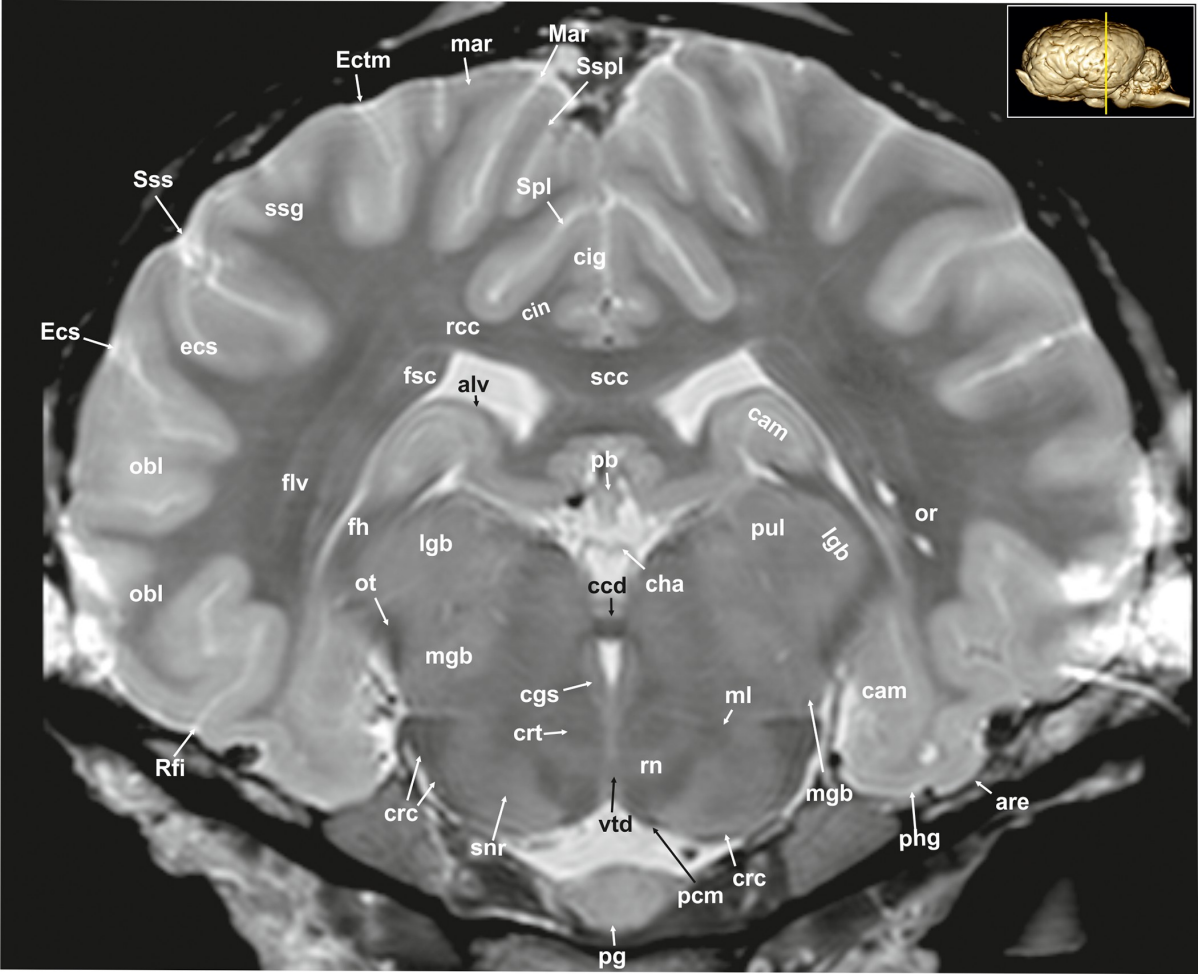
Transverse magnetic resonance image of the equine brain on the level of the caudal commissure. are: entorhinal area, alv: alveus; cam: ammon’s horn, ccd: caudal commissure, cdc: caudal colliculus, cgs: central grey substance, cha: habenular commissure, cig: cingulate gyrus, cin: cingulum, crc: cerebral crus, crt: rubro- cerebello-thalamic tract, df: dentate fascia, ecs: ectosylvian gyrus, Ecs: ectosylvian sulcus, Ectm: ectomarginal suclcus, flv: ventral longitudinal fasciculus, fsc: subcallosal fasciculus, lgb: lateral geniculate body, mar: marginal gyrus, Mar: marginal sulcus, mgb: medial geniculate body, ml: medial lemniscus, obl: oblique gyrus, or: optic radiation, ot: optic tract, pb: pineal body, pcm: peduncles of the mammillary body, pg: pituitary gland, phg: parahippocampal gyrus, pul: pulvinar nuclei, rcc: radiation of corpus callosum, Rfi: rhinal fissure, rn: red nucleus, scc: splenium of corpus callosum, snr: substantia nigra, Spl: splenial sulcus, ssg: suprasylvian gyrus, Sspl: suprasplenial sulcus, Sss: suprasylvian sulcus, vtd: ventral tegmental decussation.

**Fig 10:**
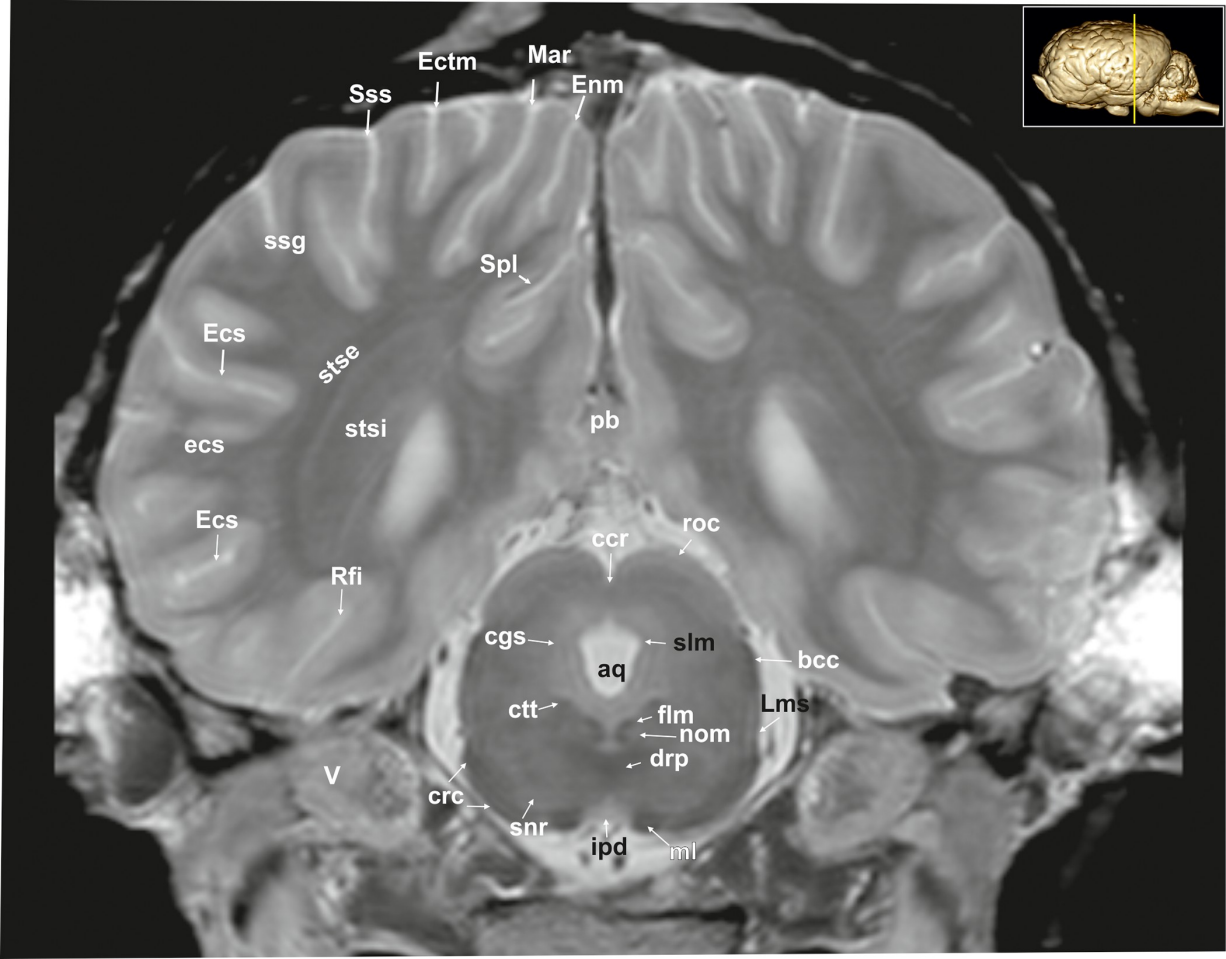
Transverse magnetic resonance image of the equine brain on the level of the rostral colliculi. aq: mesencephalic aqueduct, bcc: brachium of the caudal colliculus, ccr: commissure of the rostral colliculus, cgs: central grey substance, crc: cerebral crus, ctt: central tegmental tract, drp: decussation of the rostral cerebellar peduncles, Ecs: ectosylvian sulcus, Ectm: ectomarginal sulcus, Enm: endomarginal sulcus, flm: medial longitudinal fasciculus, ipd: interpeduncular nucleus, Lms: lateral mesencephalic sulcus, Mar: marginal sulcus, ml: medial lemniscus, nom: nucleus of the oculomotor nerve, pb: pineal body, Rfi: rhinal fissure, roc: rostral colliculus, snr: substantia nigra, Spl: splenial sulcus, stse: stratum sagittale externum, stsi: stratum sagittale internum, Sss: suprasylvian sulcus, V: trigeminal nerve.

**Fig 11:**
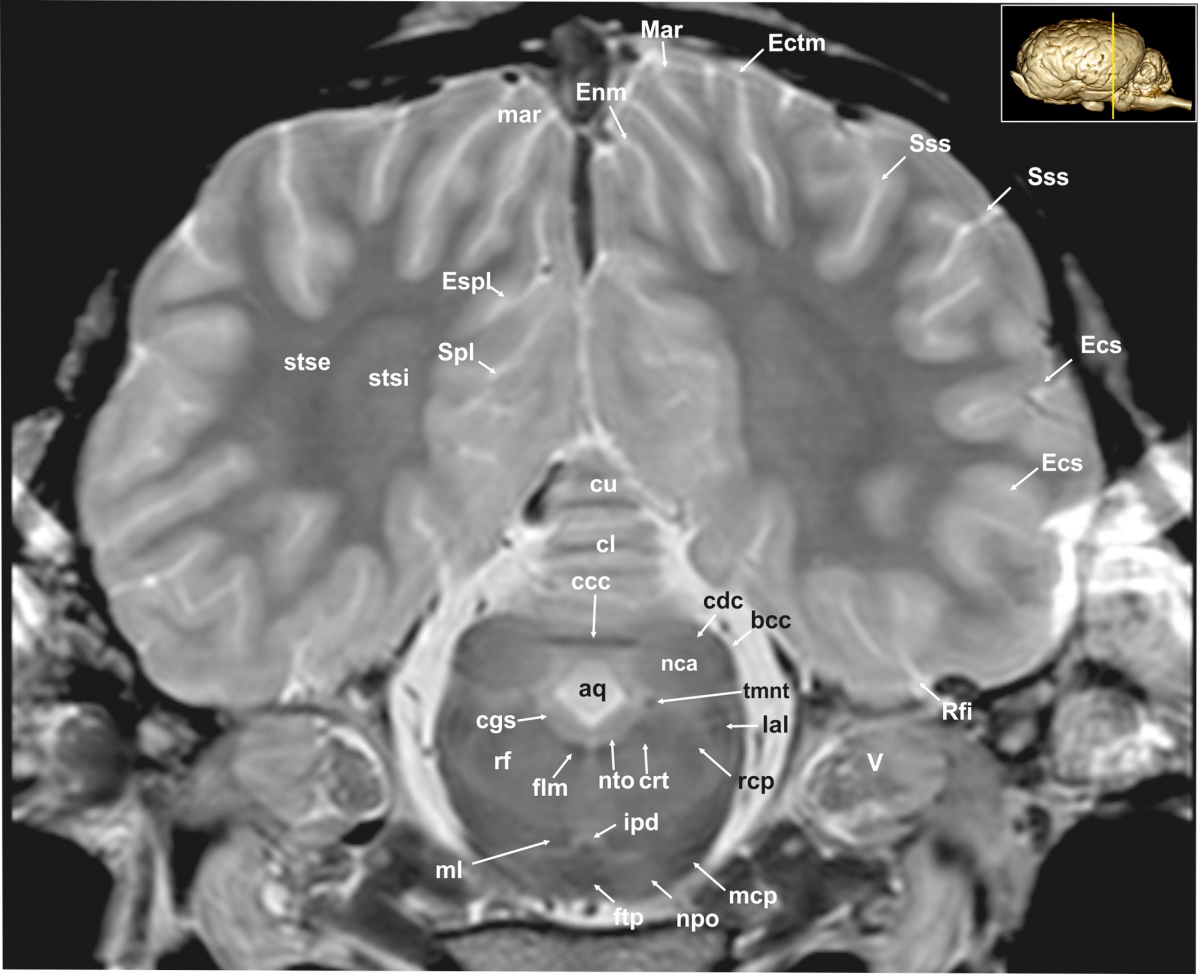
Transverse magnetic resonance image of the equine brain on the level of the caudal colliculi. aq: mesencephalic aqueduct, bcc: brachium of the caudal colliculus, ccc: commissure of the caudal colliculus, cdc: caudal colliculus, cgs: central grey substance, cl: central lobule, crt: rubro-cerebello-thalamic tract, cu: culmen, Ecs: ectosylvian sulcus, Ectm: ectomarginal sulcus, Enm: endomarginal sulcus, Espl: ectosplenial sulcus, flm: medial longitudinal fasciculus, ftp: transverse fibres of the pons, ipd: interpeduncular nucleus, lal: lateral lemniscus, mar: marginal gyrus, mcp: medial cerebellar peduncle, mgb: medial geniculate body, ml: medial lemniscus, nca: nucleus of the caudal colliculus, npo: nuclei of the pons, nll: Nucleus of the lateral lemniscus, nto: nucleus of trochlear nerve, rcp: rostral cerebellar peduncle, rf: reticular formation, Rfi: rhinal fissure, Spl: splenial sulcus, Sss: suprasylvian sulcus, stse: stratum sagittale externum, stsi: stratum sagittale internum, tmnt: mesencephalic tract of the trigeminal nerve, V: trigeminal nerve.

**Fig 12:**
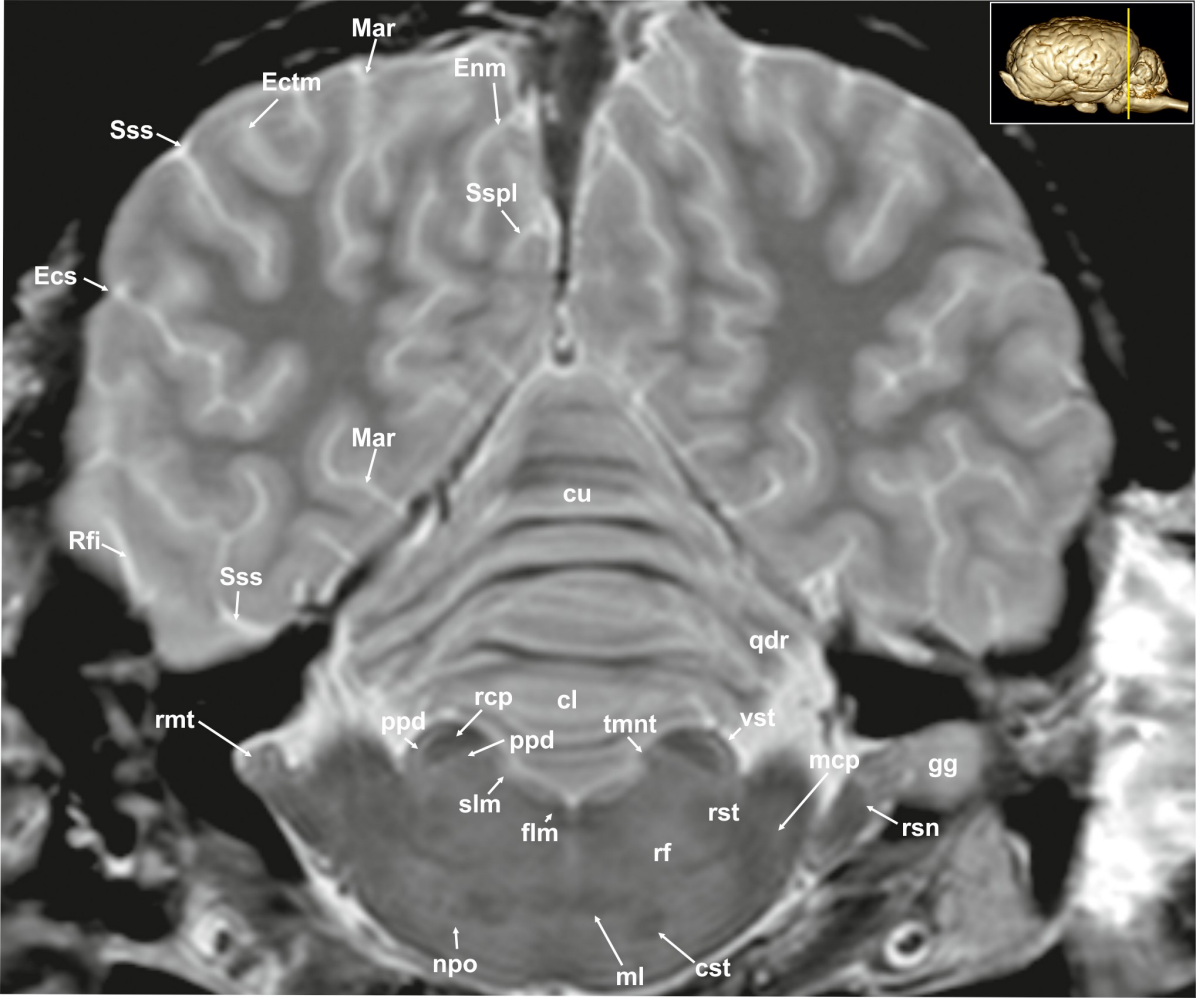
Transverse magnetic resonance image of the equine brain on the level of the rostral cerebellar peduncle. cl: central lobule, cst: corticospinal tract, cu: culmen, Ecs: ectosylvian sulcus, Ectm: ectomarginal sulcus, Enm: endomarginal sulcus, flm: medial longitudinal fasciculus, gg: Gasserian ganglion, Mar: marginal sulcus, mcp: medial cerebellar peduncle, ml: medial lemniscus, npo: nuclei of the pons, ppd: parapeduncular nuclei, qdr: quadrangulare lobule, rcp: rostral cerebellar peduncle, rf: reticular formation, Rfi: rhinal fissure, rmt: radix motoria of the trigeminal nerve, rsn: radix sensoria of the trigeminal nerve, rst: rubrospinal tract, Sspl: suprasplenial sulcus, Sss: suprasylvian sulcus, tmnt: mesencephalic tract of the trigeminal nerve, vst: vestibulospinal tract.

**Fig 13:**
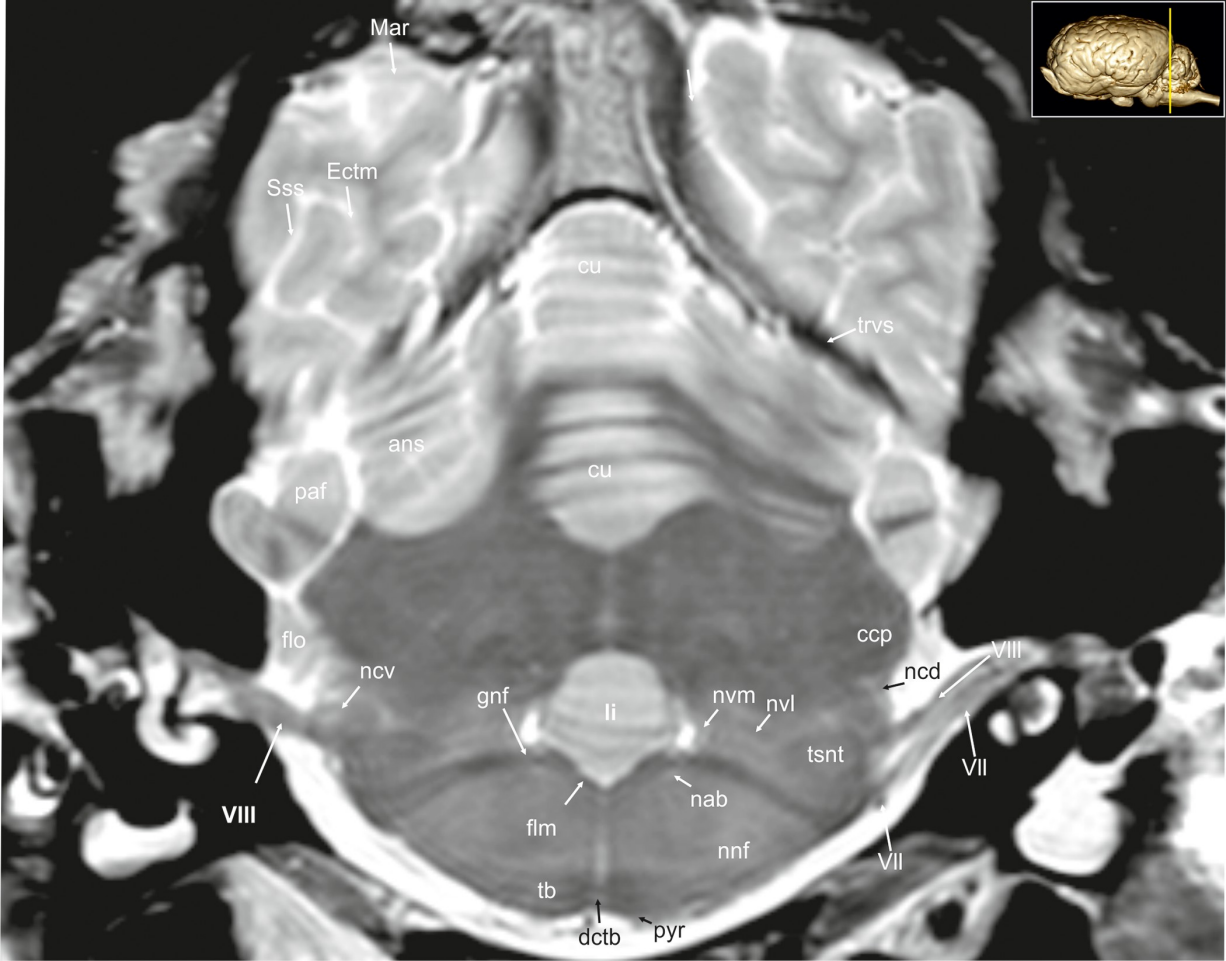
Transverse magnetic resonance image of the equine brain on the level of the genu of the facial nerve. ans: ansiform lobule, ccp: caudal cerebellar peduncle, cu: culmen, Ectm: ectomarginal sulcus, flm: medial longitudinal fasciculus, flo: flocculus, gnf: genu of the facial nerve, li: lingula of the vermis, Mar: marginal sulcus, nab: nucleus of the abducent nerve, ncd: dorsal cochlear nucleus, ncv: ventral cochlear nucleus, ndct: superior olivary nucleus, nvl: lateral vestibular nuclei, nvm: medial vestibular nucleus, paf: paraflocculus, pyr: pyramidal tract, rst: rubro-spinal tract, Sss: suprasylvian sulcus, tb: trapezoid body, trvs: transverse sinus, tsnt: spinal tract of the trigeminal nerve, qdr: quadrangular lobe, VII: facial nerve, VIII: vestibulocochleal nerve.

**Fig 14:**
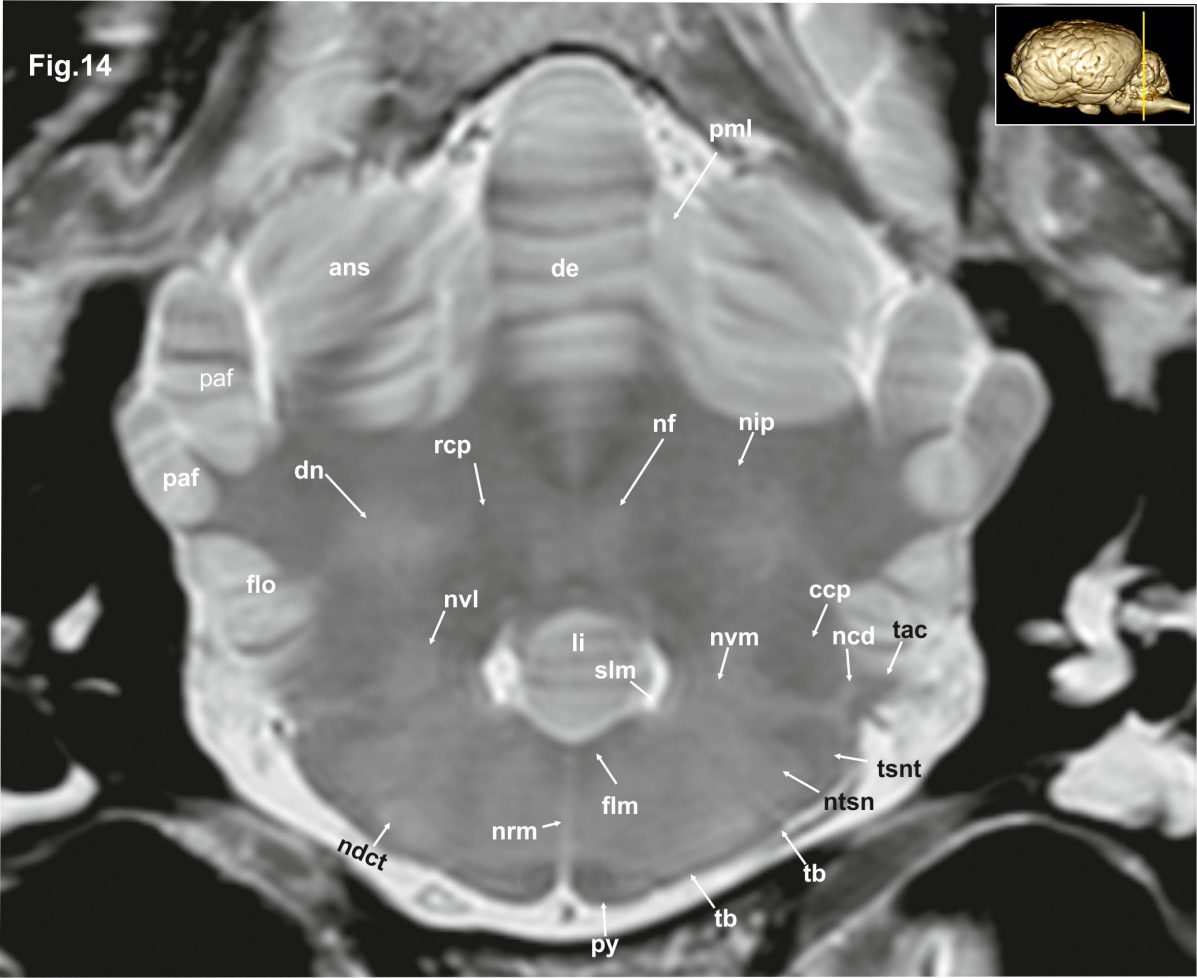
Transverse magnetic resonance image of the equine brain on the level of the acoustic tubercle. ans: ansiform lobule, ccp: caudal cerebellar peduncle, dctb: decussation of the fibres of trapezoid body, de: declive of the vermis, dn: dentate nucleus, flm: medial longitudinal fasciculus, flo: flocculus, li: lingula of the vermis, ncd: dorsal cochlear nucleus, ndct: superior olivary nucleus, nf: fastigial nucleus, nip: interpositus nucleus, ntsn: nucleus of the spinal tract of the trigeminal nerve, nvl: lateral vestibular nuclei, nvm: medial vestibular nucleus, paf: paraflocculus, pml: paramedian lobule, py: pyramis of the vermis, rcp: rostral cerebellar peduncle, slm: sulcus limitans, tac: acoustic tubercle, tb: trapezoid body, tsnt: spinal tract of the trigeminal nerve.

**Fig 15:**
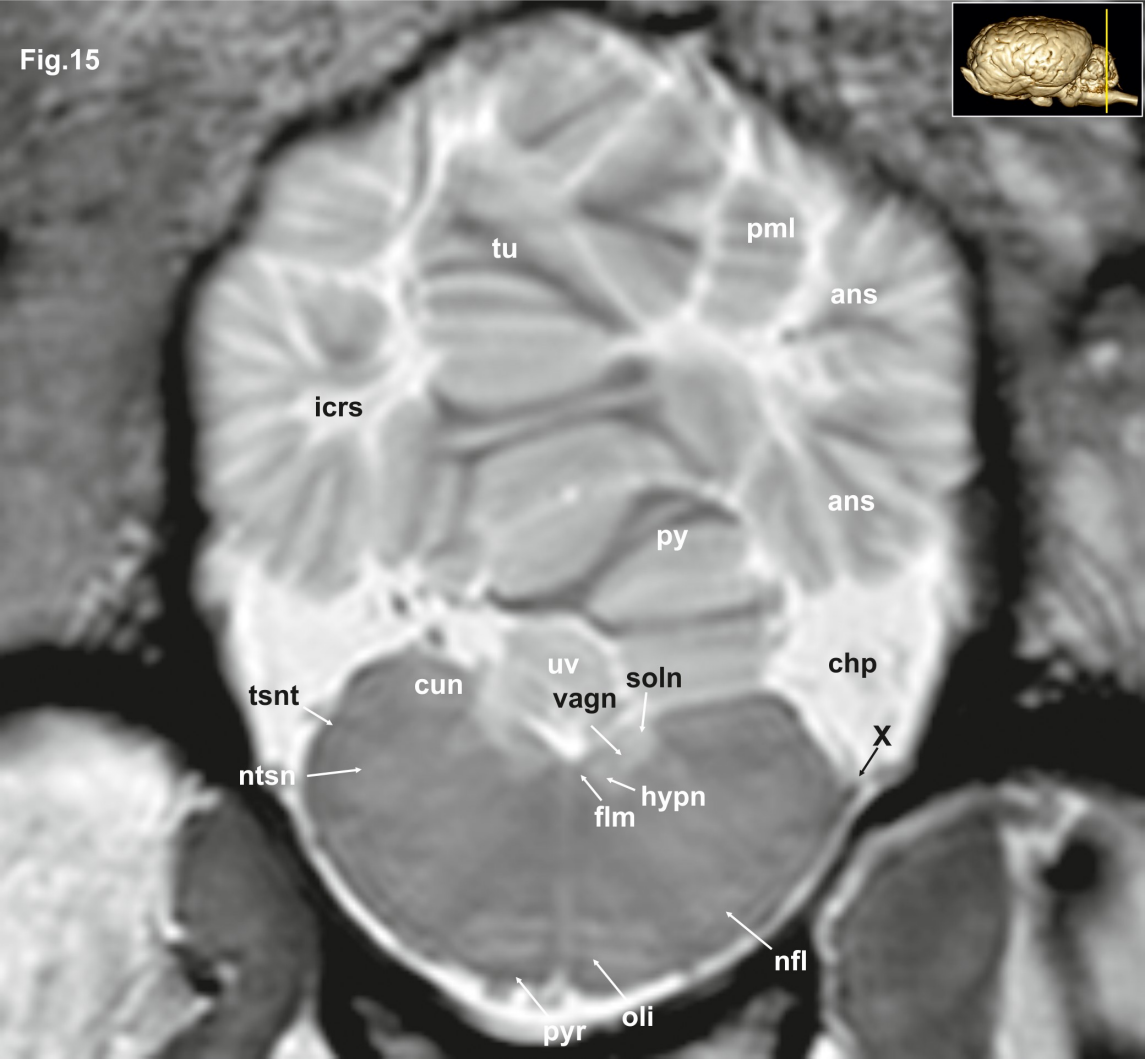
Transverse magnetic resonance image of the equine brain on the level of the cuneate nuclei. ans: ansiform lobule, chp: choroid plexus; cun: cuneate nucleus, flm: medial longitudinal fasciculus, hypn: nucleus of the hypoglossal nerve, icrs: intercrural sulcus, nfl: nucleus of the lateral fascicle, ntsn: nucleus of the spinal tract of the trigeminal nerve, oli: olivary nucleus, pml: paramedian lobule, py: pyramis of the vermis, pyr: pyramidal tract, tsnt: spinal tract of the trigeminal nerve, tu: tuber of the vermis, uv: uvula of the vermis, vagn: nucleus of the vagus nerve, X: vagus nerve.

**Fig 16:**
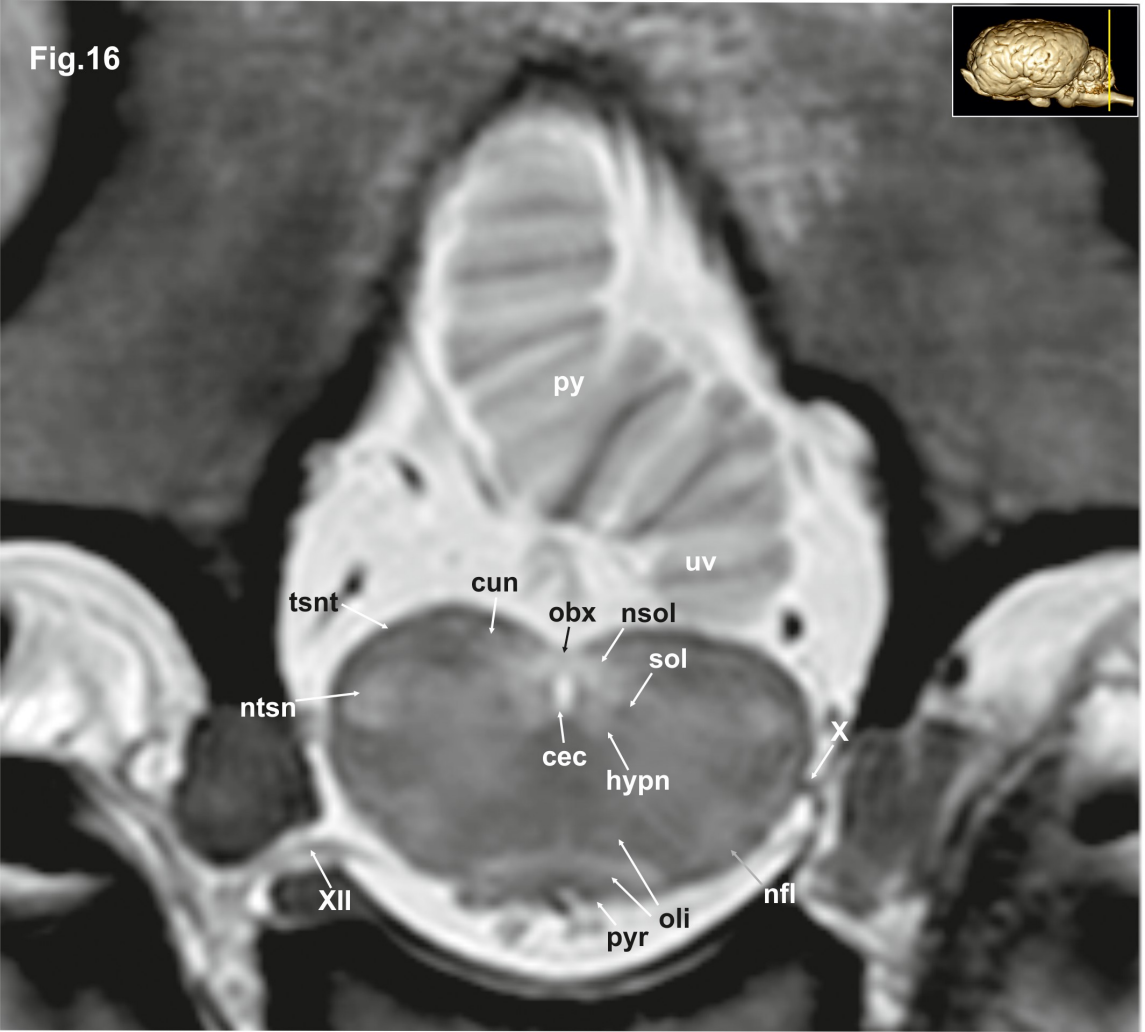
Transverse magnetic resonance image of the equine brain on the level of the obex. cun: cuneate nucleus, hypn: nucleus of the hypoglossal nerve, nfl: nucleus of the lateral fascicle, ntsn: nucleus of the spinal tract of the trigeminal nerve, oli: olivary nucleus, py: pyramis of the vermis, pyr: pyramidal tract, sol: solitary tract, tsnt: spinal tract of the trigeminal nerve, uv: uvula of the vermis, vgn: nucleus of the vagus nerve, X: vagus nerve, XII: hypoglossal nerve.

**Fig 17:**
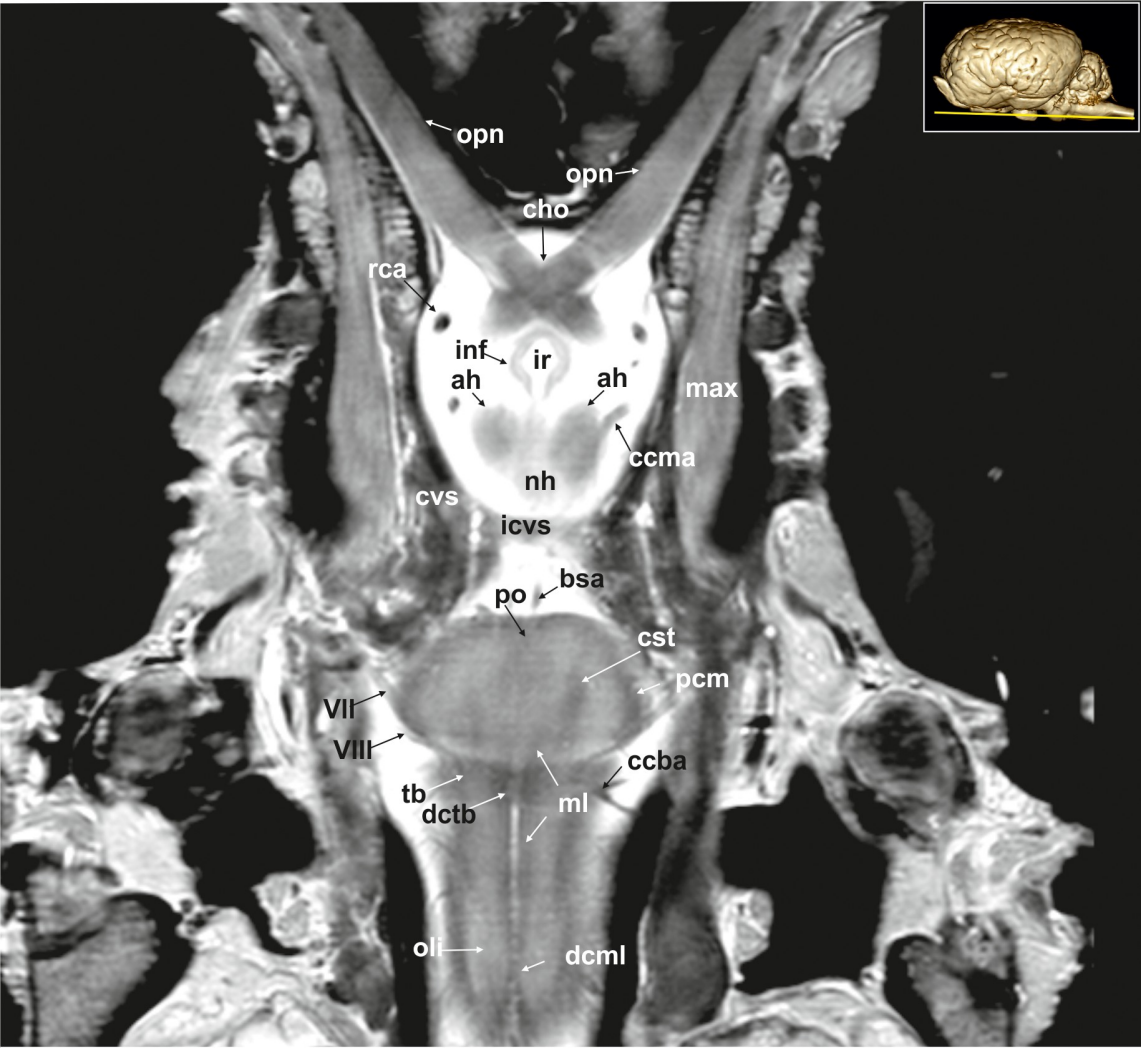
Dorsal magnetic resonance image of the equine brain on the level of the optic chiasm. ah: adenohypophysis, bsa: basilar artery, ccba: caudal cerebellar artery, ccma: caudal communicating artery, cho: optic chiasm, cst: corticospinal tract, cvs: cavernus sinus, dcml: decussation of medial lemniscus, dctb: decussation of the fibres of trapezoid body, icvs: intercavernous sinus, inf: infundibular stalk, ir: infundibular recess, max: maxillary nerve, ml: medial lemniscus, nh: neurohypophysis, oli: olivary nucleus, opn: optic nerve, pcm: peduncles of the mammillary body, po: pons, rca: rostral cerebral artery, tb: trapezoid body, VII: facial nerve, VIII: vestibulocochleal nerve.

**Fig 18:**
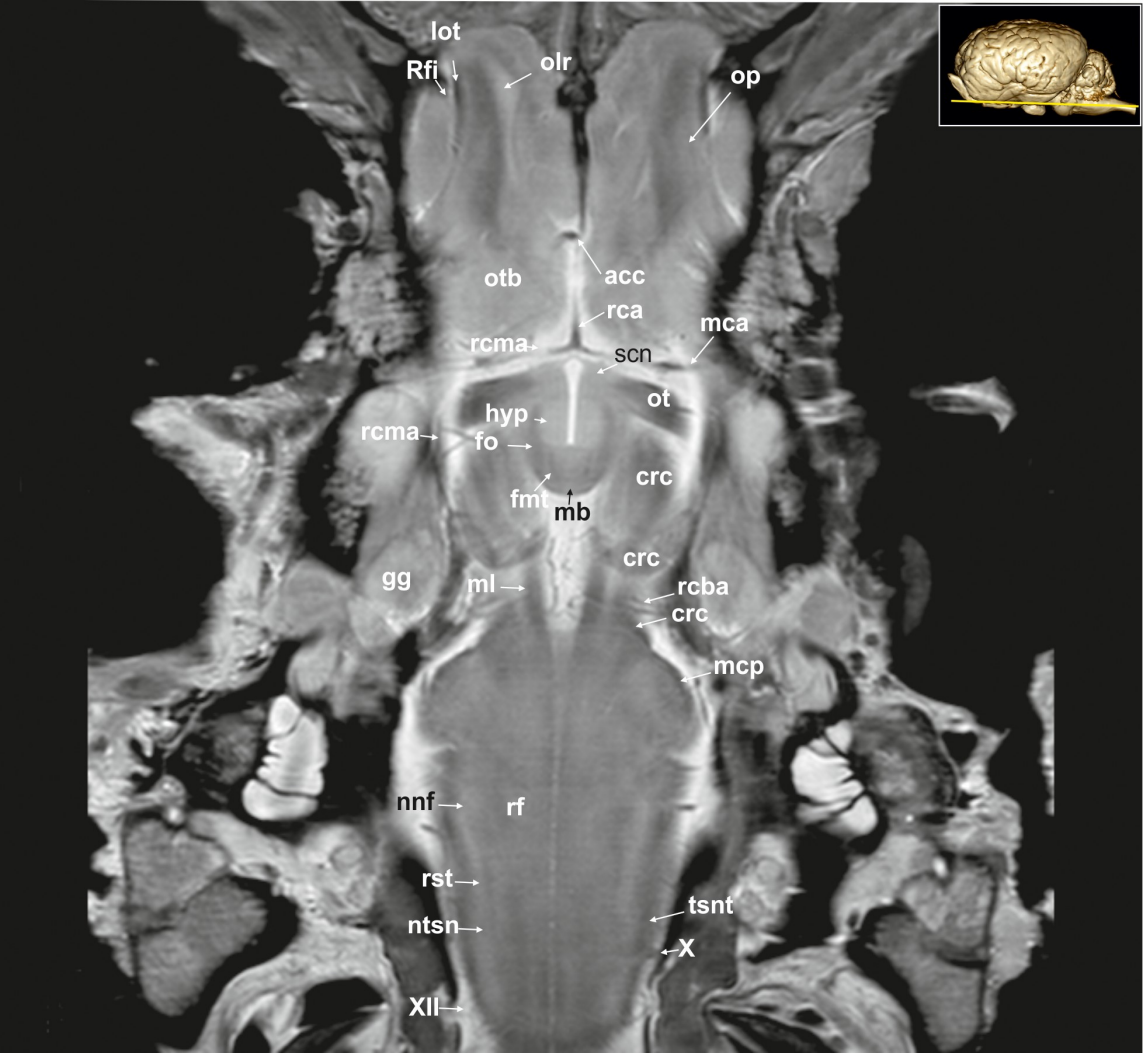
Dorsal magnetic resonance image of the equine brain on the level of the mamillary body. acc: corpus callosum artery, crc: cerebral crus, fmt: mammilo-thalamic fasciculus, fo: fornix, gg: Gasserian ganglion, hyp: hypothalamus, lot: lateral olfactory tract, mb: mamillary body, mca: medial cerebral artery, mcp: medial cerebellar peduncle, ml: medial lemniscus, nnf: nucleus of the facial nerve, ntsn: nucleus of the spinal tract of the trigeminal nerve, olr: olfactory recess, op: olfactory peduncle, ot: optic tract, otb: olfactory tubercle, rca: rostral cerebral artery, rcba: rostral cerebellar artery, rcma: rostral communicating artery, rf: reticular formation, Rfi: rhinal fissure, rst: rubrospinal tract, scn: supracommissural nucleus hypothalami, tsnt: spinal tract of the trigeminal nerve, X: vagus nerve, XII: hypoglossal nerve.

**Fig 19:**
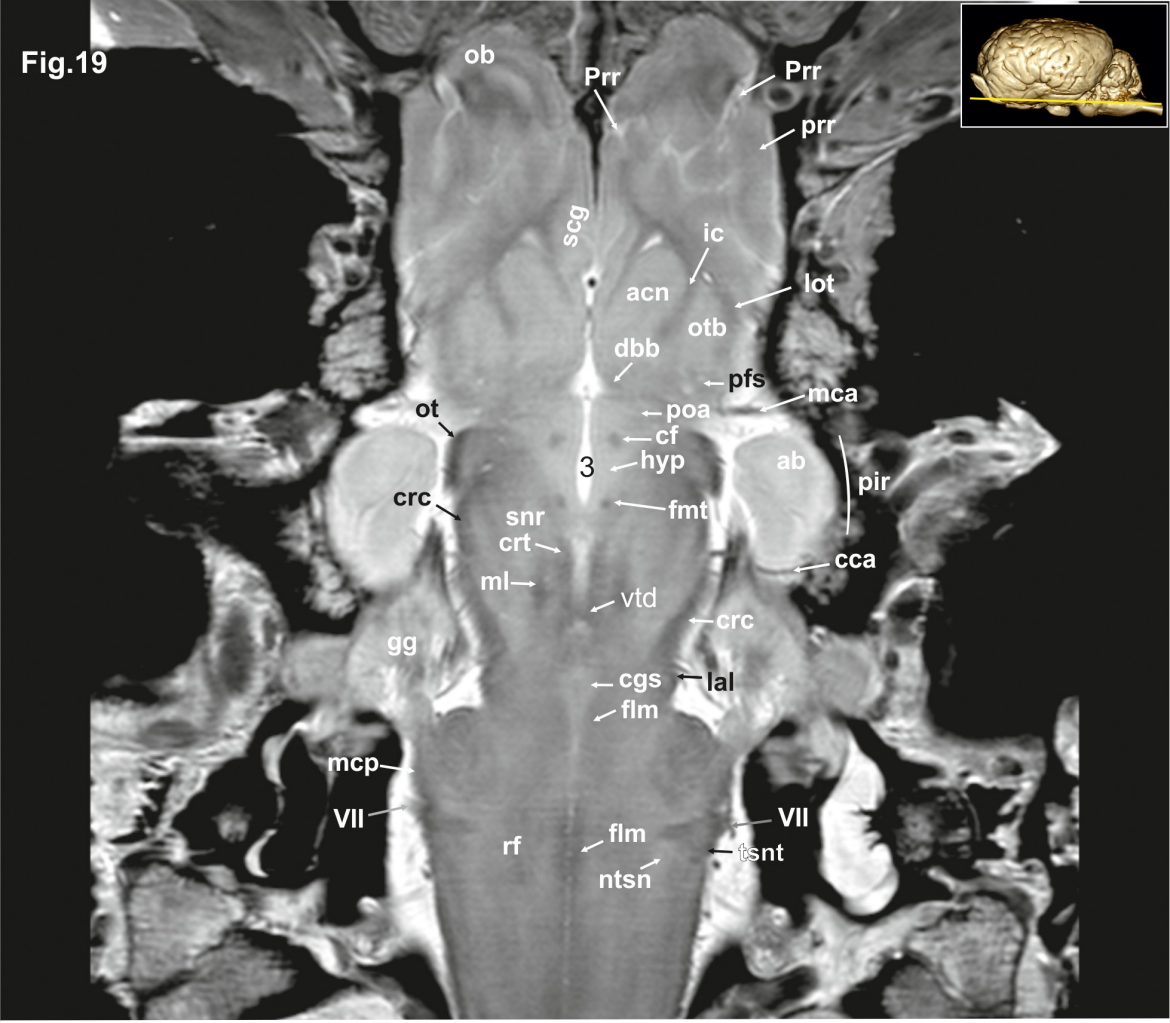
Dorsal magnetic resonance image of the equine brain on the level of the amygdaloid nuclei. ab: amygdaloid body, acn: accumbens nucleus, cca: caudal cerebral artery, cf: column of fornix, cgs: central grey substance, crc: cerebral crus, crt: rubro-cerebello-thalamic tract, dbb: diagonal band of broca, flm: medial longitudinal fasciculus, fmt: mammilo-thalamical fasciculus, gg: Gasserian ganglion, hyp: hypothalamus, ic: internal capsule, IaI: lateral lemniscus, lot: lateral olfactory tract, mca: medial cerebral artery, mcp: medial cerebellar peduncle, ml: medial lemniscus, ntsn: nucleus of the spinal tract of the trigeminal nerve, ob: olfactory bulb, ot: optic tract, otb: olfactory tubercle, pfs: perforated substance, pir: piriform lobe, poa: preoptic area, prr: prorean gyrus, Prr: prorean sulcus, rf: reticular formation, scg: subcallosal gyrus, snr: substantia nigra, tsnt: spinal tract of the trigeminal nerve, vtd: ventral tegmental decussation, VII: facial nerve, 3: third ventricle.

**Fig 20:**
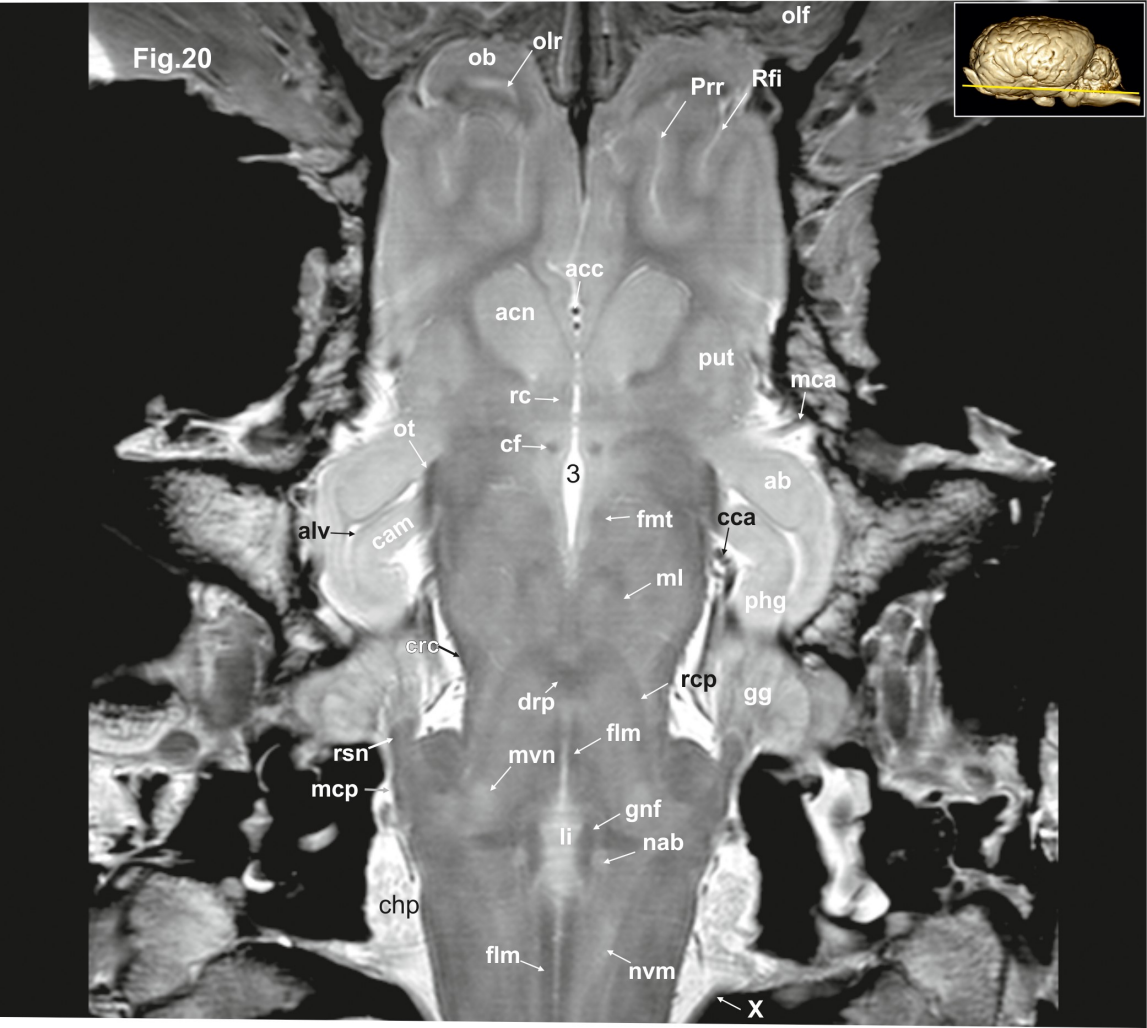
Dorsal magnetic resonance image of the equine brain on the level of the lingula. ab: amygdaloid body, acc: corpus callosum artery, acn: accumbens nucleus, cam: ammon’s horn, cca: caudal cerebral artery, chp: choroid plexus, crc: cerebral crus, drp: decussation of the rostral cerebellar peduncles, fh: fimbria of the hippocampus, flm: medial longitudinal fasciculus, fmt: mammilo-thalamical fasciculus, fo: fornix, gg: Gasserian ganglion, gnf: genu of the facial nerve, li: lingula of the vermis, mca: medial cerebral artery, mcp: medial cerebellar peduncle, ml: medial lemniscus, nab: nucleus of the abducent nerve, nvm: medial vestibular nucleus, ob: olfactory bulb, olf: olfactory fibres, olr: olfactory recess, ot: optic tract, phg: parahippocampal gyrus, Prr: prorean sulcus, put: putamen, rc: rostral commissure, rcp: rostral cerebellar peduncle, Rfi: rhinal fissure, rsn: radix sensoria of the trigeminal nerve, X: vagus nerve, III: third ventricle.

**Fig 21:**
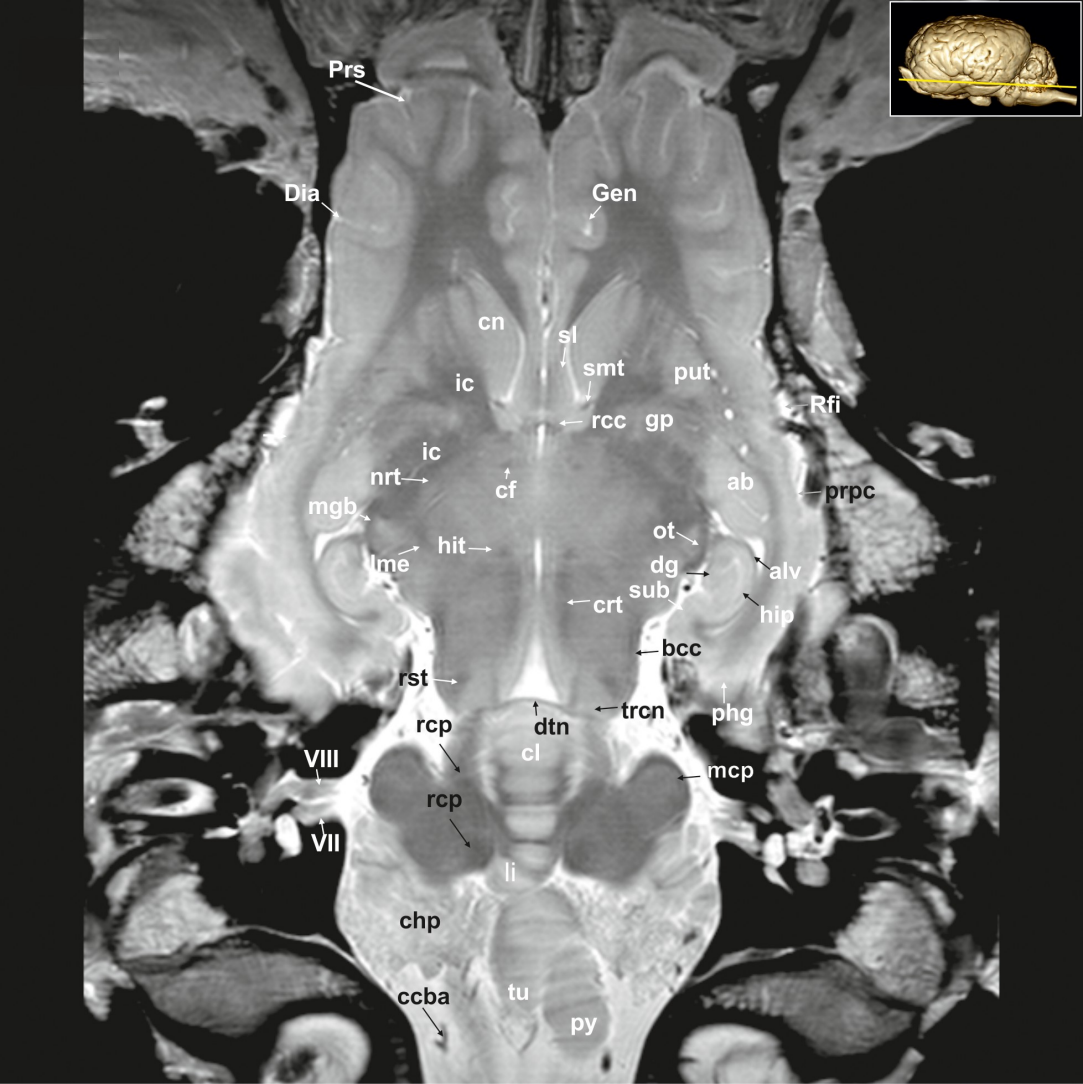
Dorsal magnetic resonance image of the equine brain with visible gyri and sulci. ab: amygdaloid body, alv: alveus, bcc: brachium of the caudal colliculus, ccba: caudal cerebellar artery, cfo: corpus of fornix, chp: choroid plexus, cl: central lobule, cn: caudate nucleus, crt: rubro-cerebello-thalamic tract, df: dentate fascia, Dia: diagonal sulcus, dtn: decussation of trochlear nerve, Gen: genual sulcus, gp: globus pallidus, hip: hippocampus proper, hit: habenulo-interpeduncular tract, ic: internal capsule, li: lingula of the vermis, lme: external medullary lamina, mcp: medial cerebellar peduncle, mgb: medial geniculate body, nrt: reticular nucleus of the thalamus, ot: optic tract, phg: parahippocampal gyrus, prpc: praepiriform cortex, Prs: presylvian sulcus, put: putamen, py: pyramis of the vermis, rcc: radiation of corpus callosum, rcp: rostral cerebellar peduncle, Rfi: rhinal fissure, rst: rubrospinal tract, smt: stria medullaris thalami, sl: lateral septal nuclei, sub: subiculum, trcn: trochlear nucleus, tu: tuber of the vermis, VII: facial nerve, VIII: vestibulocochleal nerve.

**Fig 22:**
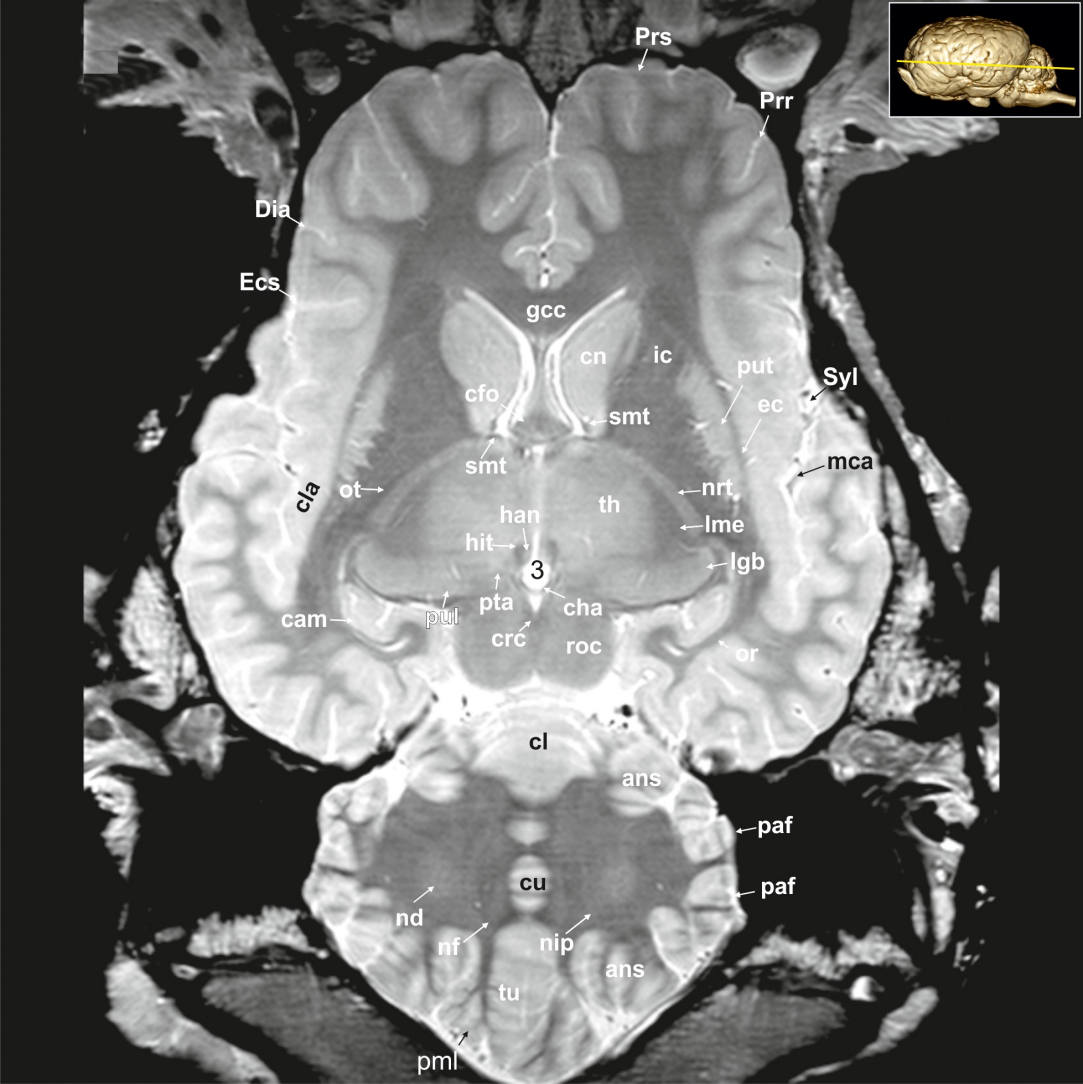
Dorsal magnetic resonance image of the equine brain on the level of the rostral colliculi. ans: ansiform lobule, cam: ammon’s horn, cfo: corpus of fornix, cha: habenular commissure, cl: central lobule, cla: claustrum, cn: caudate nucleus, crc: cerebral crus, cu: culmen, Dia: diagonal sulcus, ec: external capsule, Ecs: ectosylvian sulcus, gcc: genu of the corpus callosum, han: habenular nuclei, hit: habenulo-interpeduncular tract, ic: internal capsule, lgb: lateral geniculate body, lme: external medullary lamina, mca: medial cerebral artery, nd: dentate nucleus, nf: fastigial nucleus, nip: interpositus nucleus, nrt: reticular nucleus of the thalamus, or: optic radiation, ot: optic tract, paf: paraflocculus, pml: paramedian lobule, Prr: prorean sulcus, Prs: presylvian sulcus, pta: pretectal area, pul: pulvinar nuclei, put: putamen, roc: rostral colliculus, smt: stria medullaris thalami, Syl: sylvian fissure, th: thalamus, tu: tuber of the vermis, 3: third ventricle.

A 3D rendered model (Fig 1A–1E) demonstrates the morphology of the brain as a whole from a dorsal, lateral, frontal, and ventral as well as from a midsagittal-medial view. T2-weighted high-resolution images with detailed structural identification are provided in transverse, sagittal and dorsal planes shown in Figs 2–34. Based on comparison with histological slices, morphological structures of the adult equine brain are labelled in detail in the MR images. A list of anatomical terms and the figures, in which they are displayed, are shown in the Supporting information (S1 Table). The transverse images correspond to a reference line presented along with the 3D model in the right upper corner of the image. Selected examples of histological slides corresponding to the transverse MR-images are presented as Supporting material (S1–15 Figs). Anatomical structures were named according to the Nomina Anatomica Veterinaria [22]. A description of the brain parts, their substructures and connections is given in the following.

**Fig 23:**
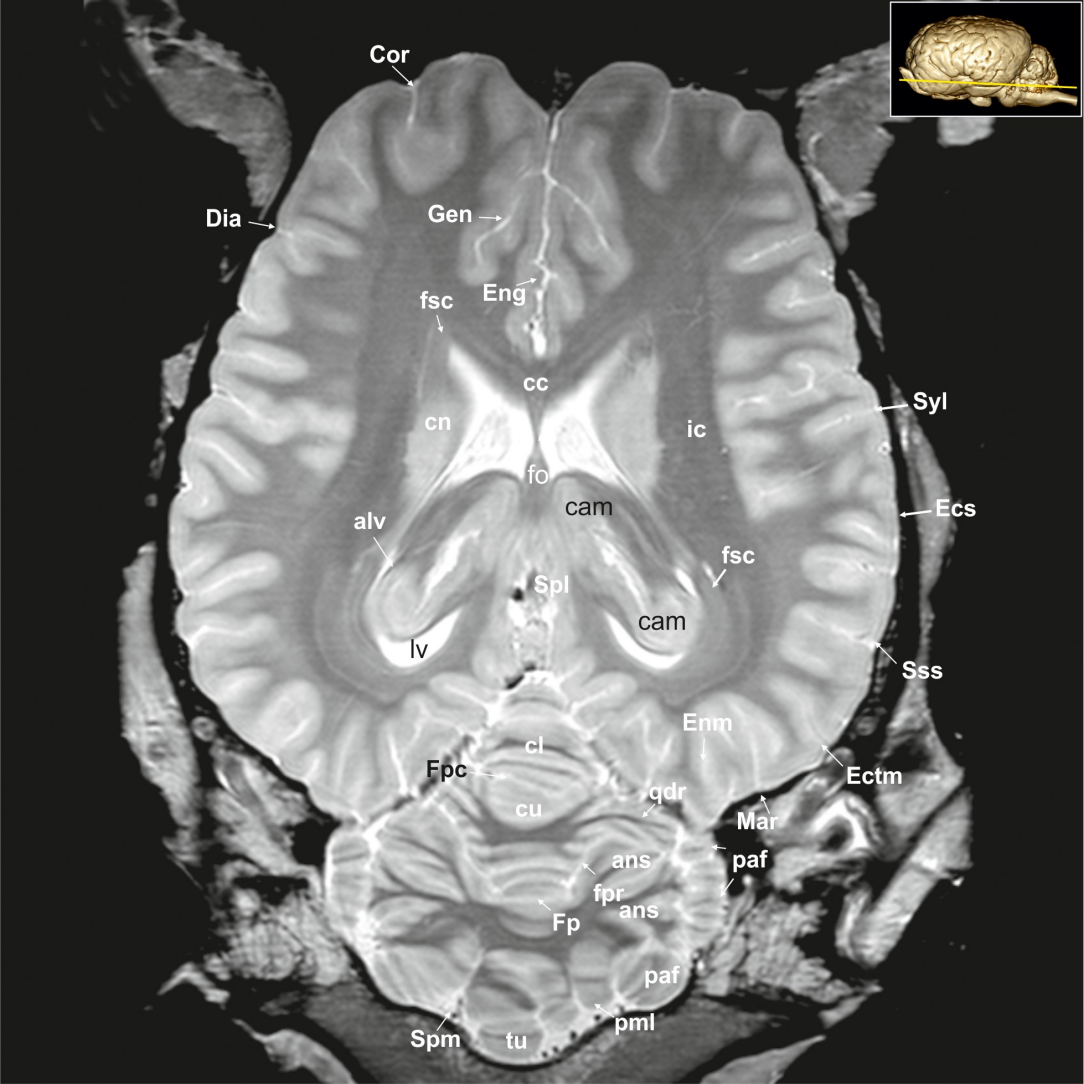
Dorsal magnetic resonance image of the equine brain on the level of the pineal gland. alv: alveus, ans: ansiform lobule, cam: ammon’s horn, cc: corpus callosum, cl: central lobule, cn: caudate nucleus, Cor: coronal sulcus, cu: culmen, Dia: diagonal sulcus, Ecs: ectosylvian sulcus, Ectm: ectomarginal sulcus, Eng: endogenual sulcus, Enm: endomarginal sulcus, fo: fornix, Fp: primary fissure, fpaf: paraflocculare fissure, Fpc: praeculminate fissure, fsc: subcallosal fasciculus, Gen: genual sulcus, ic: internal capsule, lv: lateral ventricle, Mar: marginal sulcus, paf: paraflocculus, pml: paramedian lobule, qdr: quadrangulare lobule, Spl: splenial sulcus, Spm: paramedian sulcus, Sss: suprasylvian sulcus, Syl: sylvian fissure, tu: tuber of the vermis.

**Fig 24:**
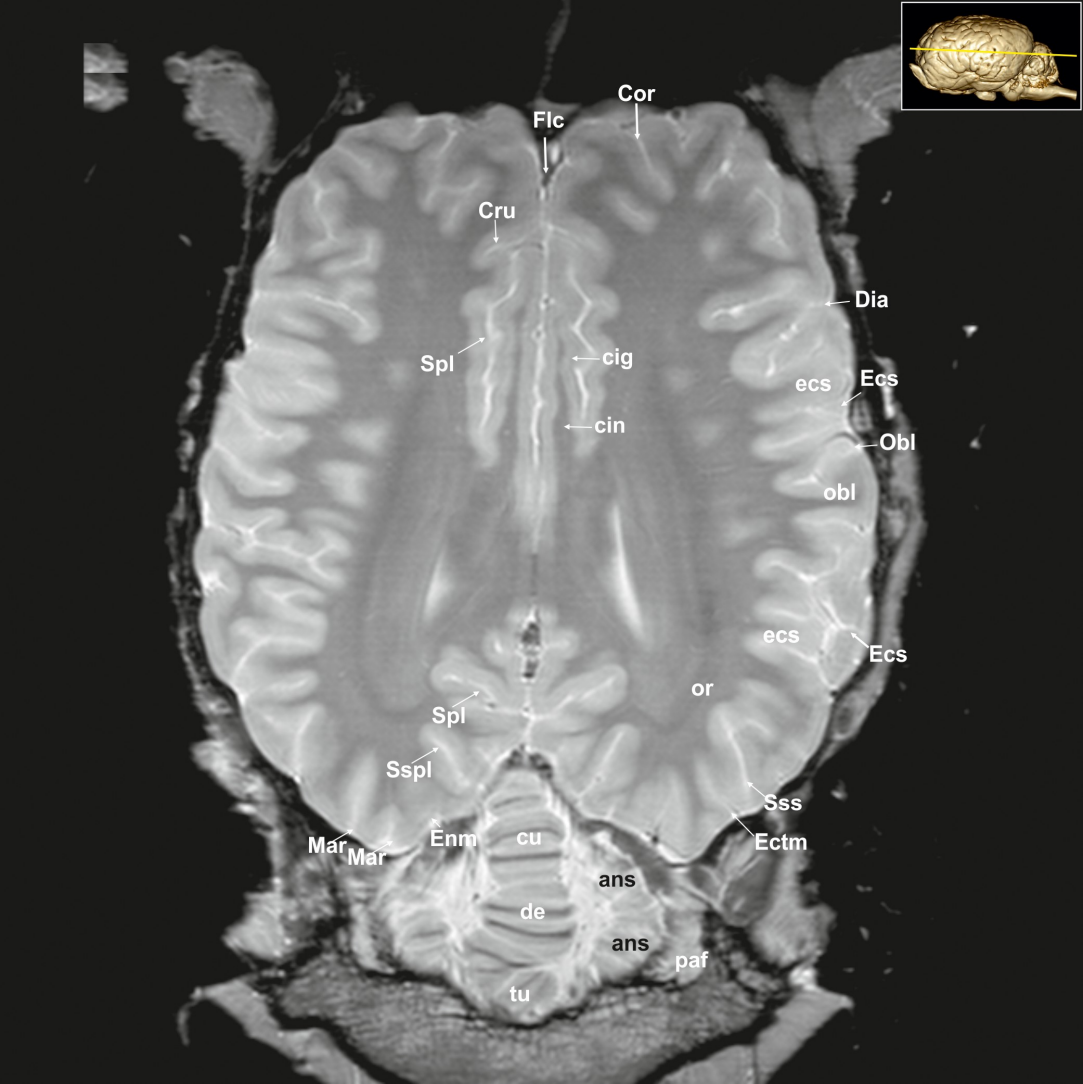
Dorsal magnetic resonance image of the equine brain on the level of the cingulated gyrus. ans: ansiform lobule, ci: cingulum, Cor: coronal sulcus, Cru: cruciate sulcus, cu: culmen, de: declive of the vermis, Dia: diagonal sulcus, Ecs: ectosylvian sulcus, ecs: ectosylvian gyrus, Ectm: ectomarginal sulcus, Enm: endomarginal sulcus, Flc: longitudinal cerebral fissure, Mar: marginal sulcus, Obl: oblique sulcus, obl: oblique gyrus, or: optic radiation, paf: paraflocculus, Spl: splenial sulcus, Sspl: suprasplenial sulcus, Sss: suprasylvian sulcus, tu: tuber of the vermis.

**Fig 25:**
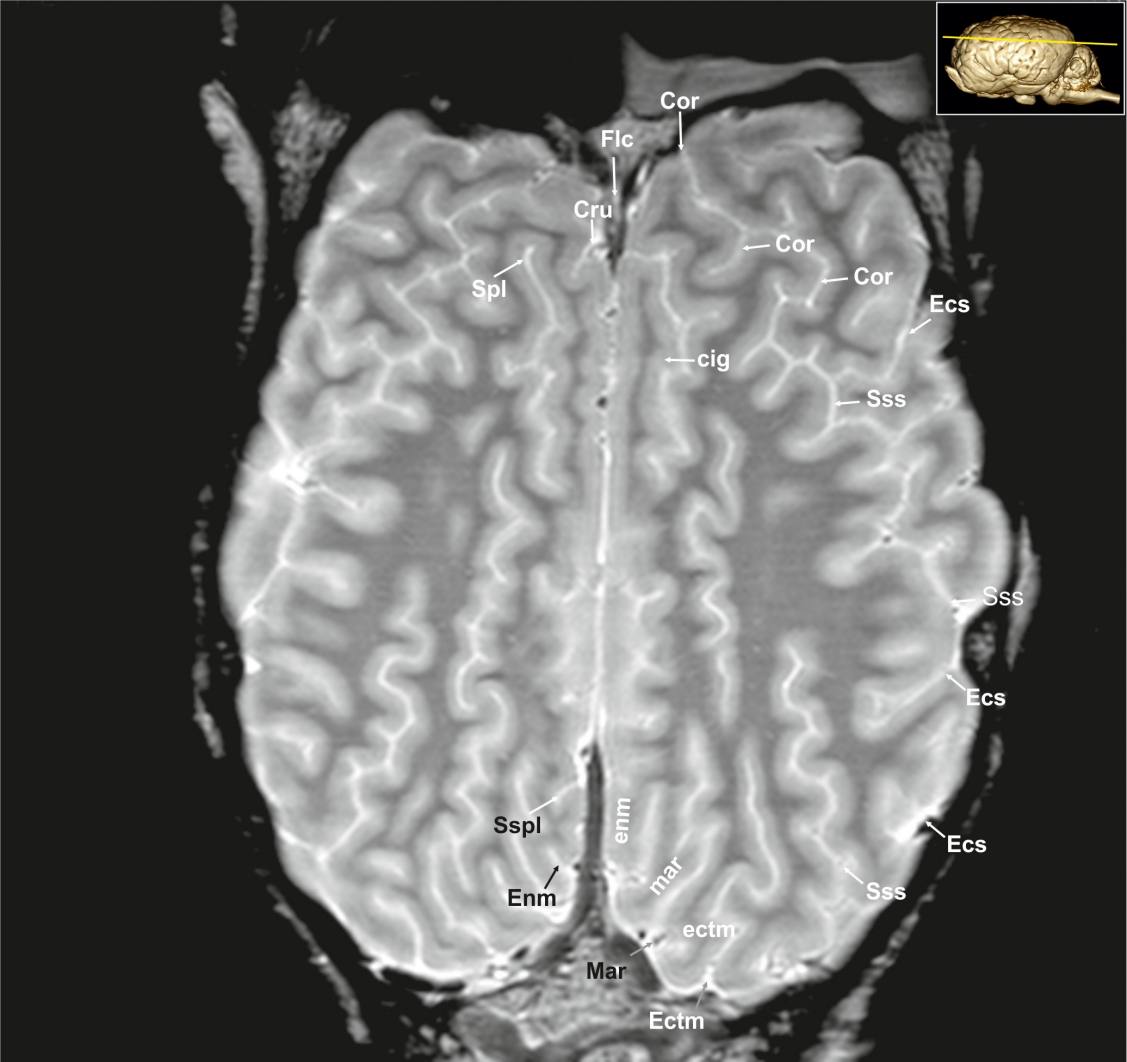
Dorsal magnetic resonance image of the equine brain on the level above the corpus callosum and cingulate gyrus. cig: cingulate gyrus, cin: cingulum, Cor: coronal sulcus, Cru: cruciate sulcus, Ecs: ectosylvian sulcus, Ectm: ectomarginal sulcus, ectm: ectomarginal gyrus, Enm: endomarginal sulcus, enm: endomarginal gyrus, Flc: longitudinal cerebral fissure, Mar: marginal sulcus, mar: marginal gyrus, Spl: splenial sulcus, Sspl: suprasplenial sulcus, Sss: suprasylvian sulcus.

**Fig 26:**
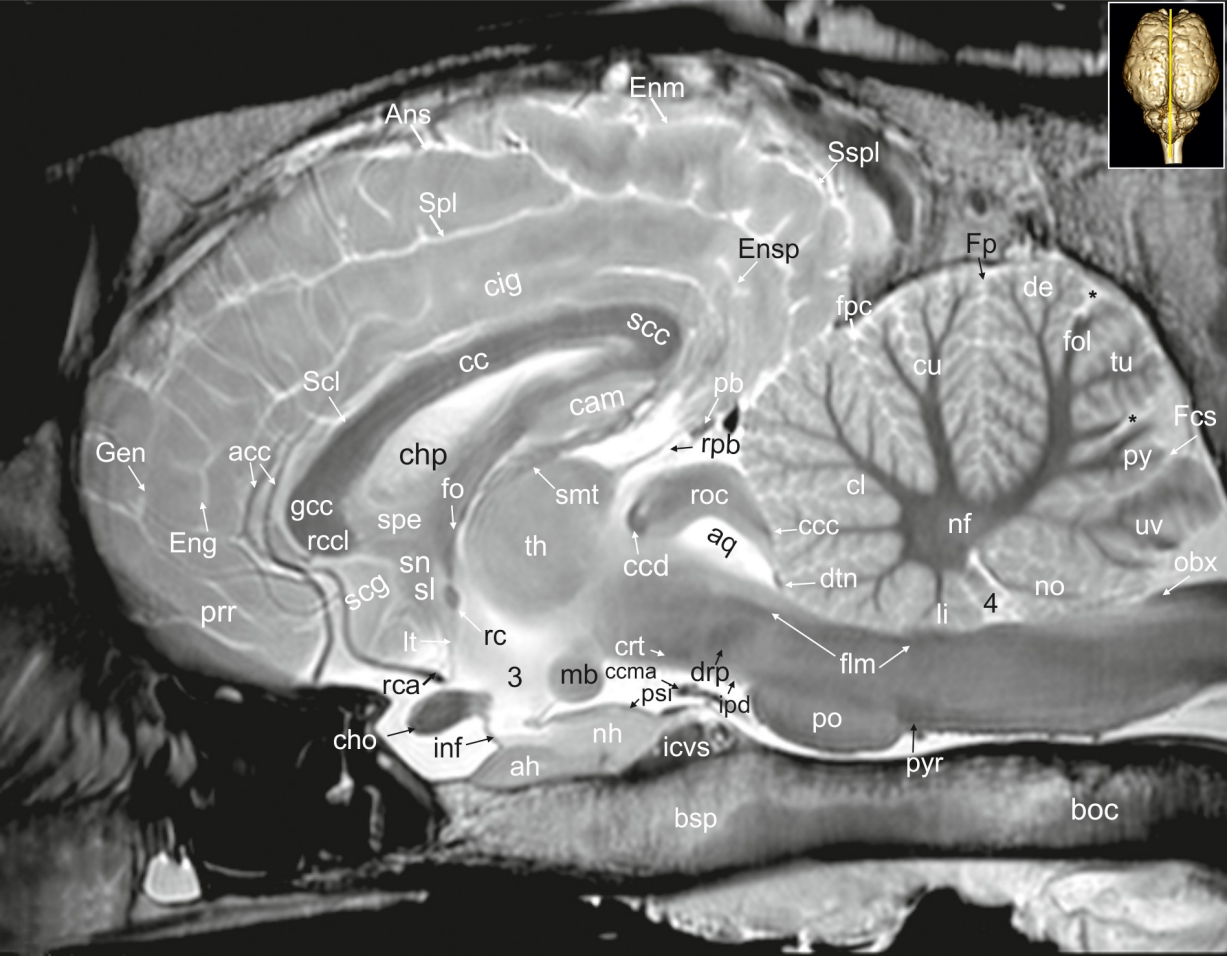
Mid-sagittal magnetic resonance image of the equine brain. acc: corpus callosum artery, ah: adenohypophysis, Ans: ansate sulcus, aq: mesencephalic aqueduct, boc: basioccipital bone, bsp: basisphenoidal bone, cam: ammon’s horn, cc: corpus callosum, ccc: commissure of the caudal colliculus, ccd: caudal commissure, ccma: caudal communicating artery, cho: optic chiasm, chp: choroid plexus, cig: cingulate gyrus, cin: cingulum, cl: central lobule, crt: rubro-cerebello-thalamic tract, cu: culmen, de: declive of the vermis, drp: decussation of the rostral cerebellar peduncles, dtn: decussation of trochlear nerve, Eng: endogenual sulcus, Enm: endomarginal sulcus, Espl: ectosplenial sulcus, flm: medial longitudinal fasciculus, fo: fornix, fol: folium of the vermis, Fp: primary fissure, Fpc: praeculminate fissure, gcc: genu of the corpus callosum, Gen: genual sulcus, icvs: intercavernous sinus, inf: infundibular stalk, ipd: interpeduncular nucleus, li: lingula of the vermis, lt: terminal lamina, mb: mamillary body, nf: fastigial nucleus, nh: neurohypophysis, no: nodulus of the vermis, obx: obex, pb: pineal body, po: pons, prr: prorean gyrus, psi: pars intermedia of the pituitary gland, py: pyramis of the vermis, pyr: pyramidal tract, rc: rostral commissure, rca: rostral cerebral artery, rcc: radiation of corpus callosum, rccl: rostrum of the corpus callosum, rpb: recess of the pineal body, roc: rostral colliculus, scc: splenium of corpus callosum, Scf: secondary fissure, scg: subcallosal gyrus, Scl: sulcus of corpus callosum, sl: lateral septal nuclei, smt: stria medullaris thalami, sn: septal nuclei, Spl: splenial sulcus, Sspl: suprasplenial sulcus, th: thalamus, tu: tuber vermis, uv: uvula of the vermis, 3: third ventricle, 4: fourth ventricle. asterisk: blind ending medullary branches, uncovered with cortex.

**Fig 27:**
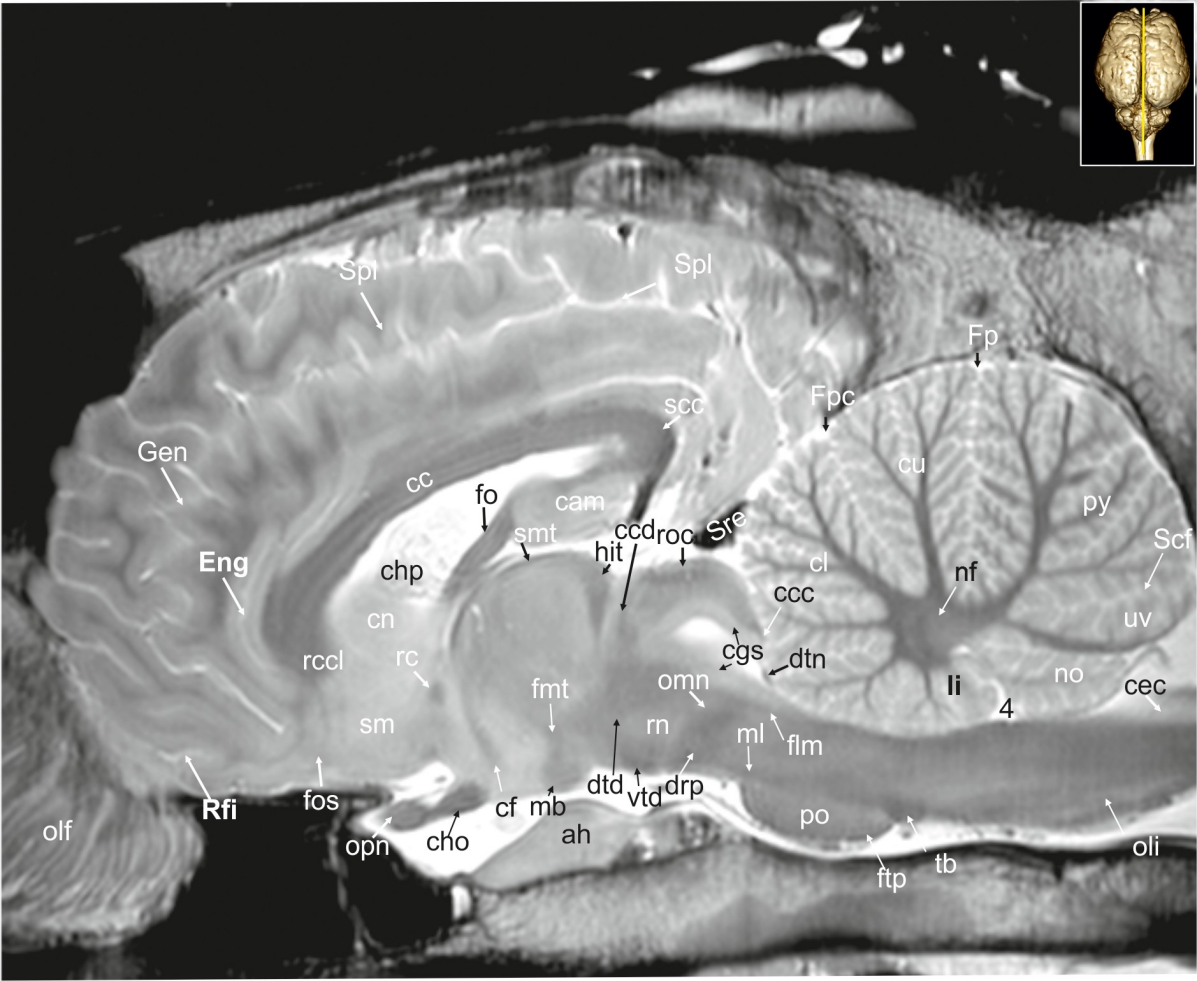
Parasagittal magnetic resonance image of the equine brain on the level of the columns of the fornix. cam: ammon’s horn, cc: corpus callosum, ccc: commissure of the caudal colliculus, ccd: caudal commissure, cec: central canal, cf: column of fornix, cgs: central grey substance, cho: optic chiasm, chp: choroid plexus, cl: central lobule, cn: caudate nucleus, cu: culmen, drp: decussation of the rostral cerebellar peduncles, dtd: dorsal tegmental decussation, dtn: decussation of trochlear nerve, Eng: endogenual sulcus, flm: medial longitudinal fasciculus, fmt: mammillo-thalamic fascicle, fo: fornix, Fp: primary fissure, Fpc: praeculminate fissure, ftp: transverse fibres of the pons, Gen: genual sulcus, hit: habenulo-interpeduncular tract, li: lingula of the vermis, mb: mamillary body, ml: medial lemniscus, nf: fastigial nucleus, no: nodulus of the vermis, olf: olfactory fibres, oli: olivary nucleus, omn: oculomotor nerve, opn: optic nerve, po: pons, py: pyramis of the vermis, rc: rostral commissure, rcc: radiation of corpus callosum, rccl: rostrum of the corpus callosum, Rfi: rhinal fissure, rn: red nucleus, roc: rostral colliculus, scc: splenium of corpus callosum, Scf: secondary fissure, sm: medial septal nuclei, smt: medullary stria of the thalamus, Spl: splenial sulcus, sre: straight sinus, tb: trapezoid body, uv: uvula of the vermis, vtd: ventral tegmental decussation, 4: fourth ventricle.

**Fig 28:**
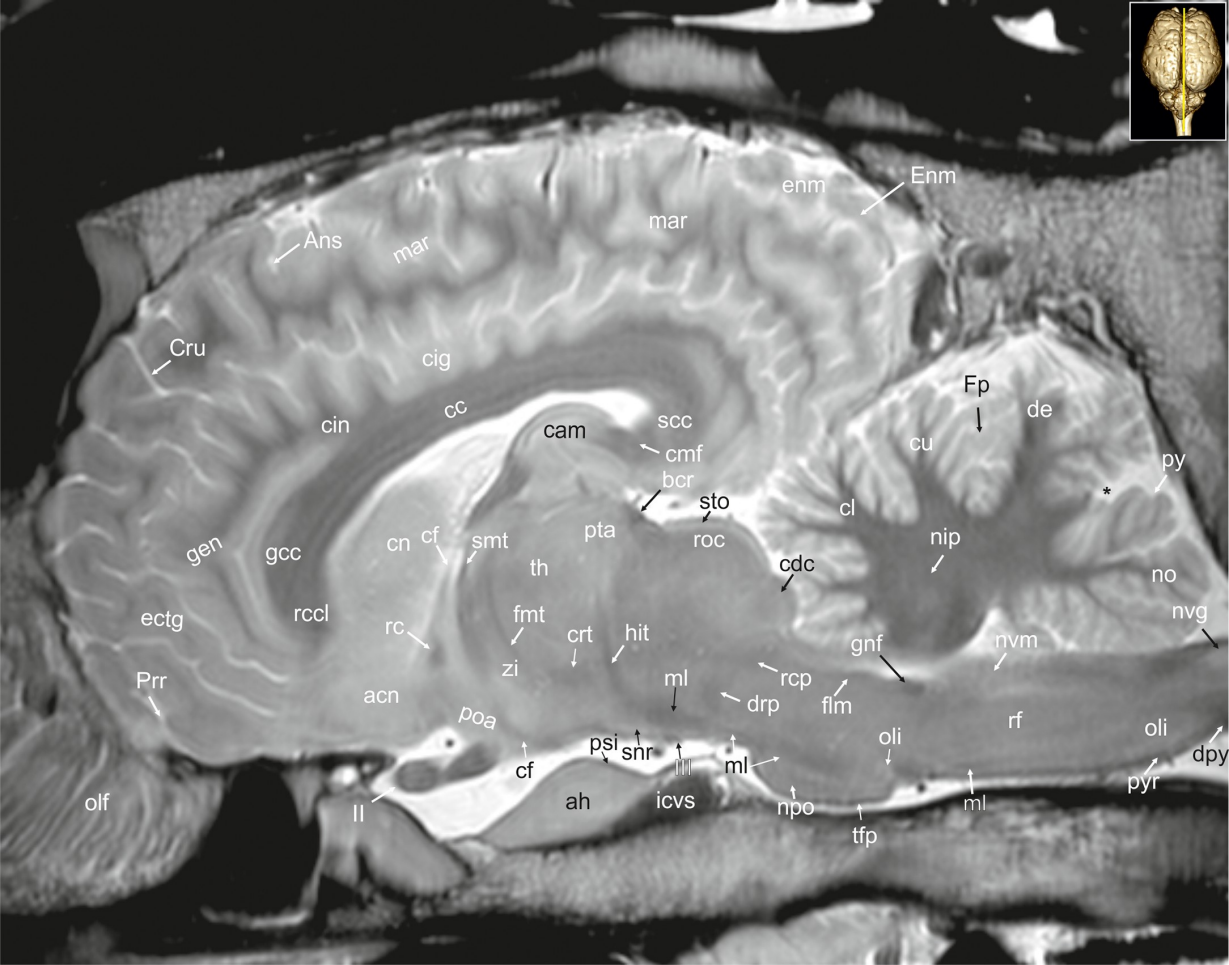
Parasagittal magnetic resonance image of the equine brain on the level of the habenulo-interpeduncular tract. acn: accumbens nucleus, ah: adenohypophysis, Ans: ansate sulcus, bcr: brachium of the rostral colliculus, cam: ammon’s horn, cc: corpus calosum, cdc: caudal colliculus, cf: column of fornix, cig: cingulate gyrus, cin: cingulum, cl: central lobule, cmf: commissure of fornix, cn: caudate nucleus, crt: rubro-cerebello-thalamic tract, Cru: cruciate sulcus, cu: culmen, de: declive of the vermis, dpy: pyramidal decussation, drp: decussation of the rostral cerebellar peduncles, ectg: ectogenual gyrus, enm: endomarginal gyrus, Enm: endomarginal sulcus, flm: medial longitudinal fasciculus, fmt: mammilo-thalamical fasciculus, Fp: primary fissure, gcc: genu of the corpus callosum, gen: genual gyrus, gnf: genu of the facial nerve, hit: habenulo-interpeduncular tract, icvs: intercavernous sinus, mar: marginal gyrus, ml: medial lemniscus, nip: interpositus nucleus, no: nodulus of the vermis, npo: nuclei of the pons, nvg: nucleus of the vagal nerve, nvm: medial vestibular nucleus, olf: olfactory fibres, oli: olivary nucleus, opn: optic nerve, poa: preoptic area, Prr: prorean sulcus, psi: pars intermedia of the pituitary gland, pta: pretectal area, py: pyramis of the vermis, pyr: pyramidal tract, rc: rostral commissure, rccl: rostrum of corpus callosum, rcp: rostral cerebellar peduncle, rf: reticular formation, roc: rostral colliculus, scc: splenium of corpus callosum, smt: medullary stria of the thalamus, snr: substantia nigra, sto: stratum opticum of the rostral colliculus, tfp: transvers fibres of pons, th: thalamus, zi: zona incerta, III: oculomotor nerve. asterisk: blind ending medullary branches, uncovered with cortex.

**Fig 29:**
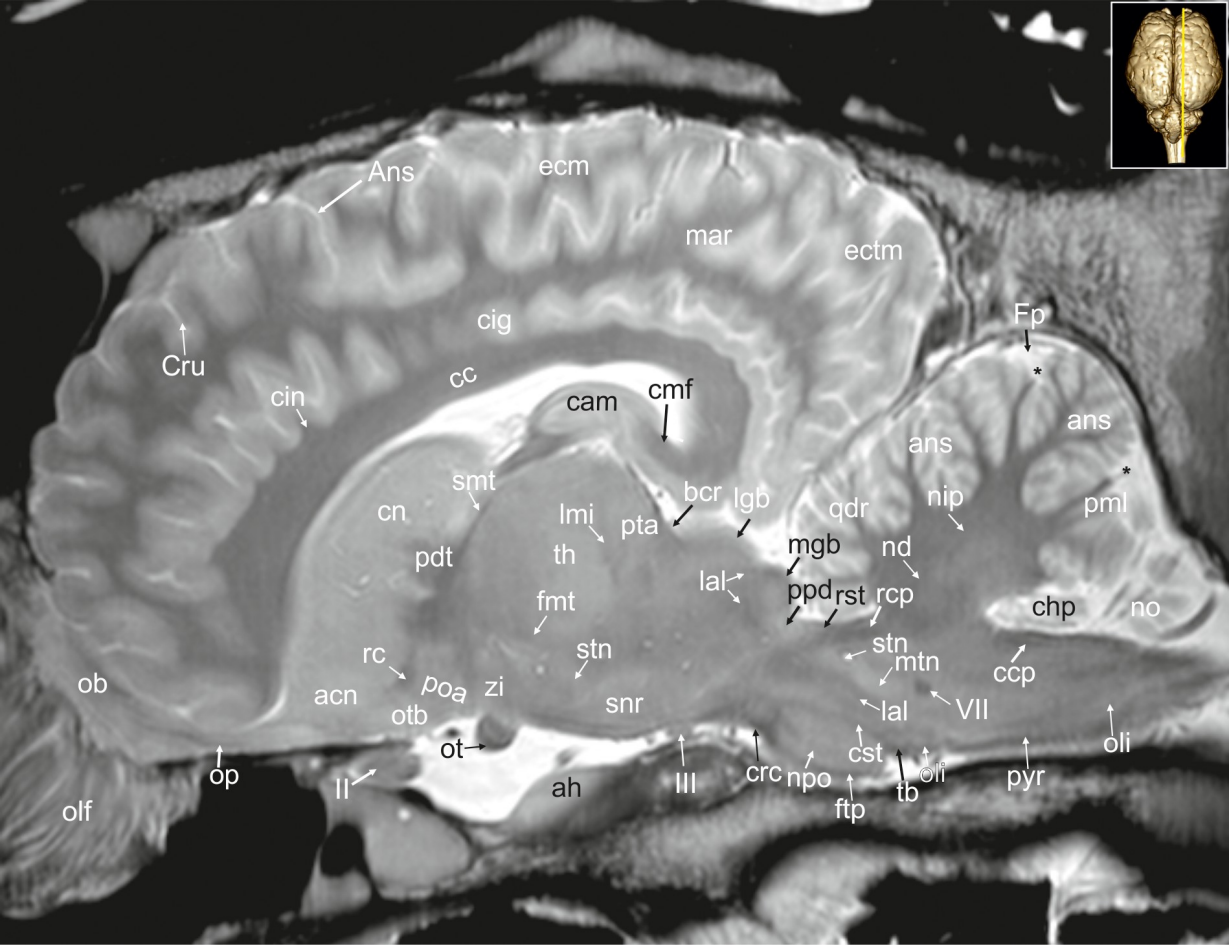
Parasagittal magnetic resonance image of the equine brain at the level of the pretectal area. acn: accumbens nucleus, ah: adenohypophysis, Ans: ansate sulcus, ans: ansiform lobule, bcr: brachium of the rostral colliculus, cam: ammon’s horn, cc: corpus callosum, ccp: caudal cerebellar peduncle, chp: choroid plexus, cig: cingulate gyrus, cin: cingulum, cmf: commissure of fornix, cn: caudate nucleus, crc: cerebral crus, Cru: cruciate sulcus, cst: corticospinal tract, ectm: ectomarginal gyrus, fmt: mammilo-thalamical fasciculus, Fp: primary fissure, ftp: transverse fibres of the pons, lal: lateral lemniscus, lgb: lateral geniculate body, lmi: internal medullary lamina, mar: marginal gyrus, mgb: medial geniculate body, mtn: motor nucleus of the trigeminal nerve, nd: dentate nucleus, nip: interpositus nucleus, no: nodulus of the vermis, npo: nuclei of the pons, ob: olfactory bulb, olf: olfactory fibres, oli: olivary nucleus, op: olfactory peduncle, ot: optic tract, otb: olfactory tubercle, pdt: thalamic peduncle, pml: paramedian lobule, poa: preoptic area, ppd: parapeduncular nuclei, pta: pretectal area, pyr: pyramidal tract, qdr: quadrangulare lobule, rc: rostral commissure, rcp: rostral cerebellar peduncle, rst: rubrospinal tract, smt: medullary stria of the thalamus, snr: substantia nigra, stn: subthalamic nucleus, stt: terminal stria, tb: trapezoid body, th: thalamus, zi: zona incerta, II: optic nerve, III: oculomotor nerve, VII: facial nerve. asterisk: blind ending medullary branches, uncovered with cortex.

**Fig 30:**
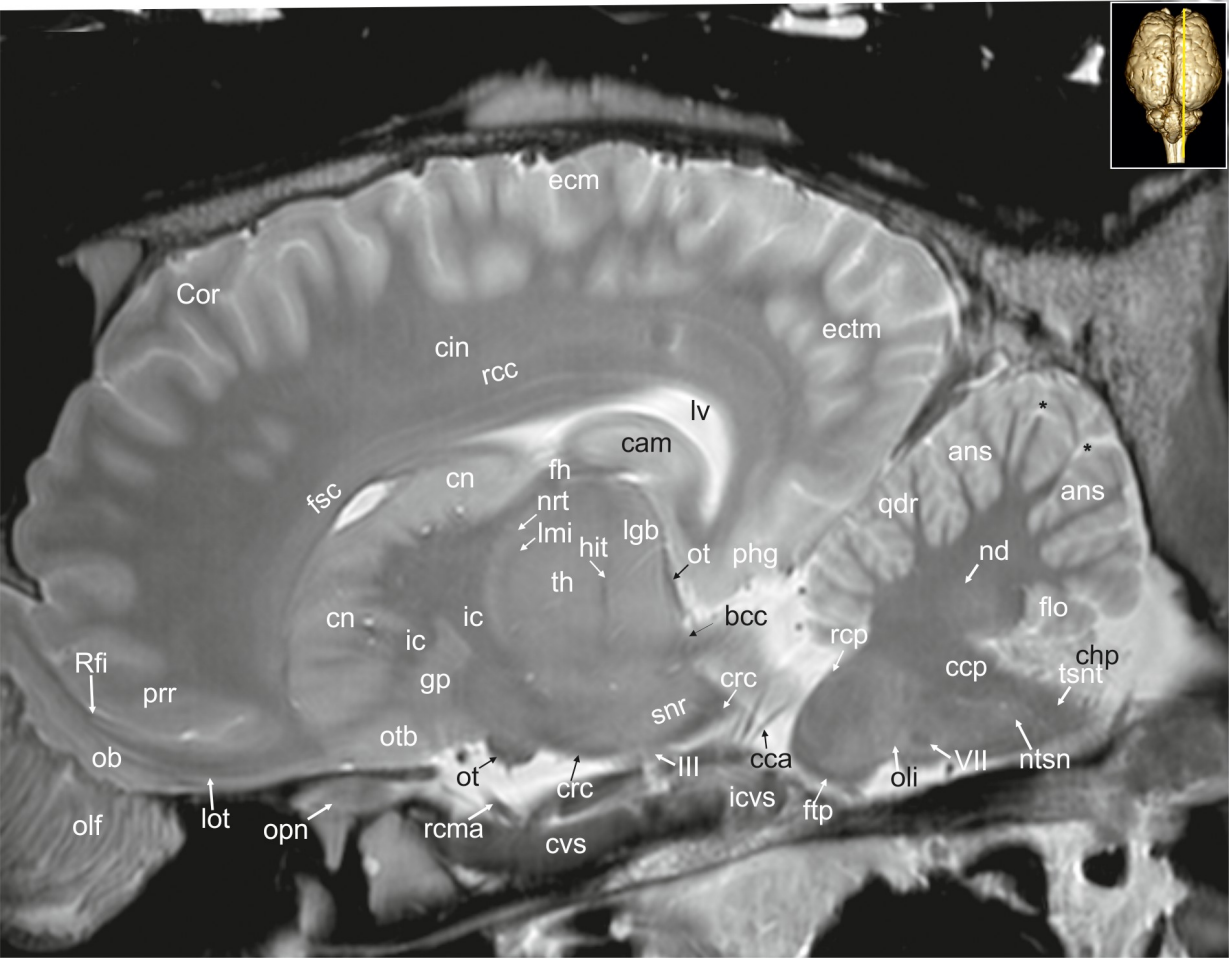
Parasagittal magnetic resonance image of the equine brain cerebral crus. ans: ansiform lobule, bcc: brachium of the caudal colliculus, cam: ammon’s horn, cca: caudal cerebral artery, ccp: caudal cerebellar peduncle, chp: choroid plexus, cin: cingulum, cn: caudate nucleus, Cor: coronal sulcus, crc: cerebral crus, cvs: cavernus sinus, ectm: ectomarginal gyrus, fh: fimbria of the hippocampus, flo: flocculus, fsc: subcallosal fasciculus, ftp: transverse fibres of the pons, gp: globus pallidus, hit: habenulo-interpeduncular tract, ic: internal capsule, icvs: intercavernous sinus, lgb: lateral geniculate body, lmi: internal medullary lamina, lot: lateral olfactory tract, lv: lateral ventricle, nd: dentate nucleus, nrt: reticular nucleus of the thalamus, ntsn: nucleus of the spinal tract of the trigeminal nerve, ob: olfactory bulb, olf: olfactory fibres, oli: olivary nucleus, opn: optic nerve, ot: optic tract, otb: olfactory tubercle, phg: parahippocampal gyrus, prr: prorean gyrus, qdr: quadrangulare lobule, rcc: radiation of corpus callosum, rcma: rostral communicating artery, rcp: rostral cerebellar peduncle, Rfi: rhinal fissure, snr: substantia nigra, th: thalamus, tsnt: spinal tract of the trigeminal nerve, III: oculomotor nerve, VII: facial nerve.

**Fig 31:**
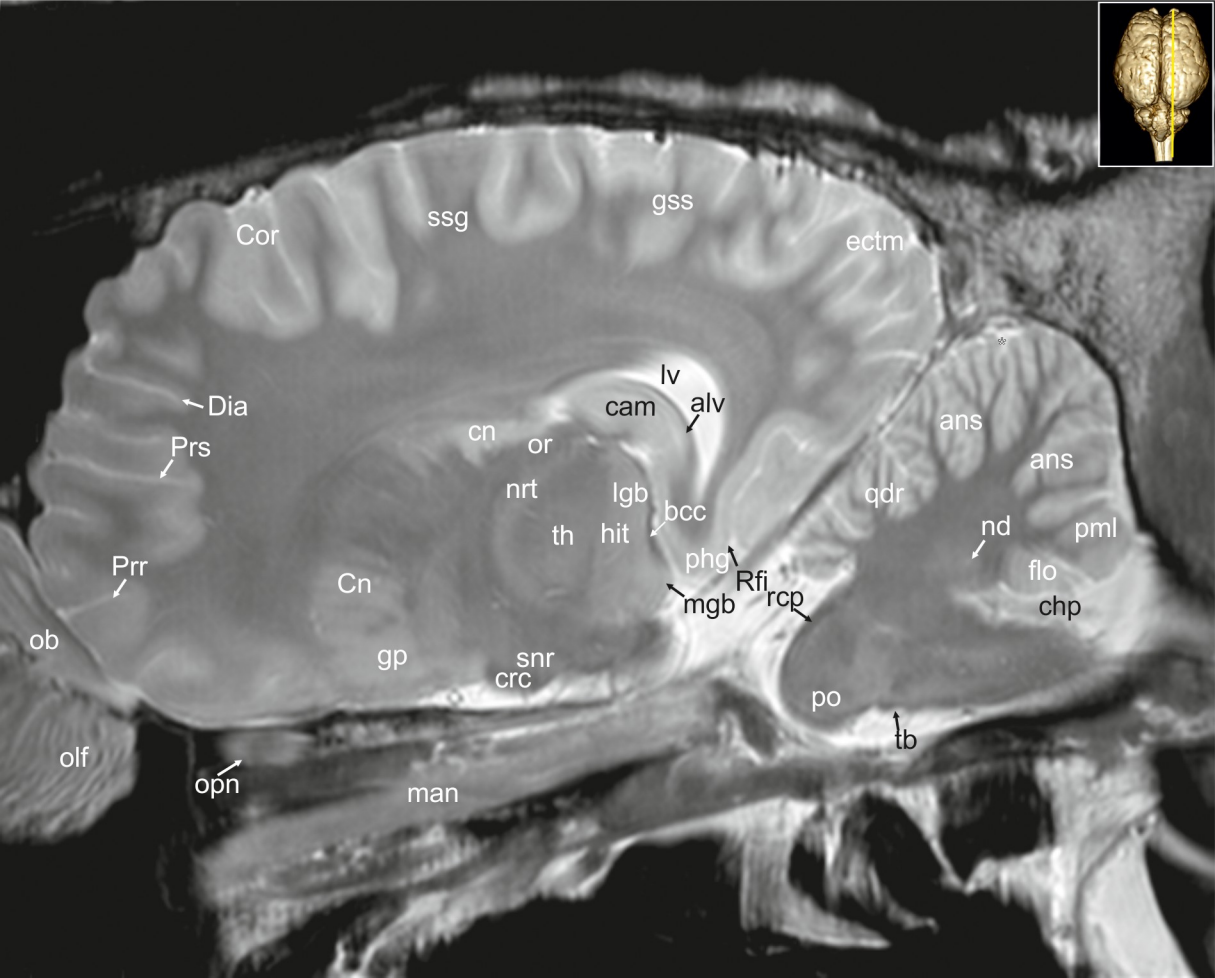
Parasagittal magnetic resonance image of the equine brain at the level of the nucleus reticularis thalami. alv: alveus, ans: ansiform lobule, bcc: brachium of the caudal colliculus, cam: ammon’s horn, chp: choroid plexus, cn: caudate nucleus, Cor: coronal sulcus, crc: cerebral crus, Dia: diagonal sulcus, ectm: ectomarginal gyrus, flo: flocculus, gp: globus pallidus, hit: habenulo-interpeduncular tract, lgb: lateral geniculate body, lv: lateral ventricle, man: mandibular nerve, mgb: medial geniculate body, nd: dentate nucleus, nrt: reticular nucleus of the thalamus, ob: olfactory bulb, olf: olfactory fibres, opn: optic nerve or: optic radiation, phg: parahippocampal gyrus, pml: paramedian lobule, po: pons, Prr: prorean sulcus, Prs: presylvian sulcus, qdr: quadrangulare lobule, rcp: rostral cerebellar peduncle, Rfi: rhinal fissure, snr: substantia nigra, ssg: suprasylvian gyrus, th: thalamus.

**Fig 32:**
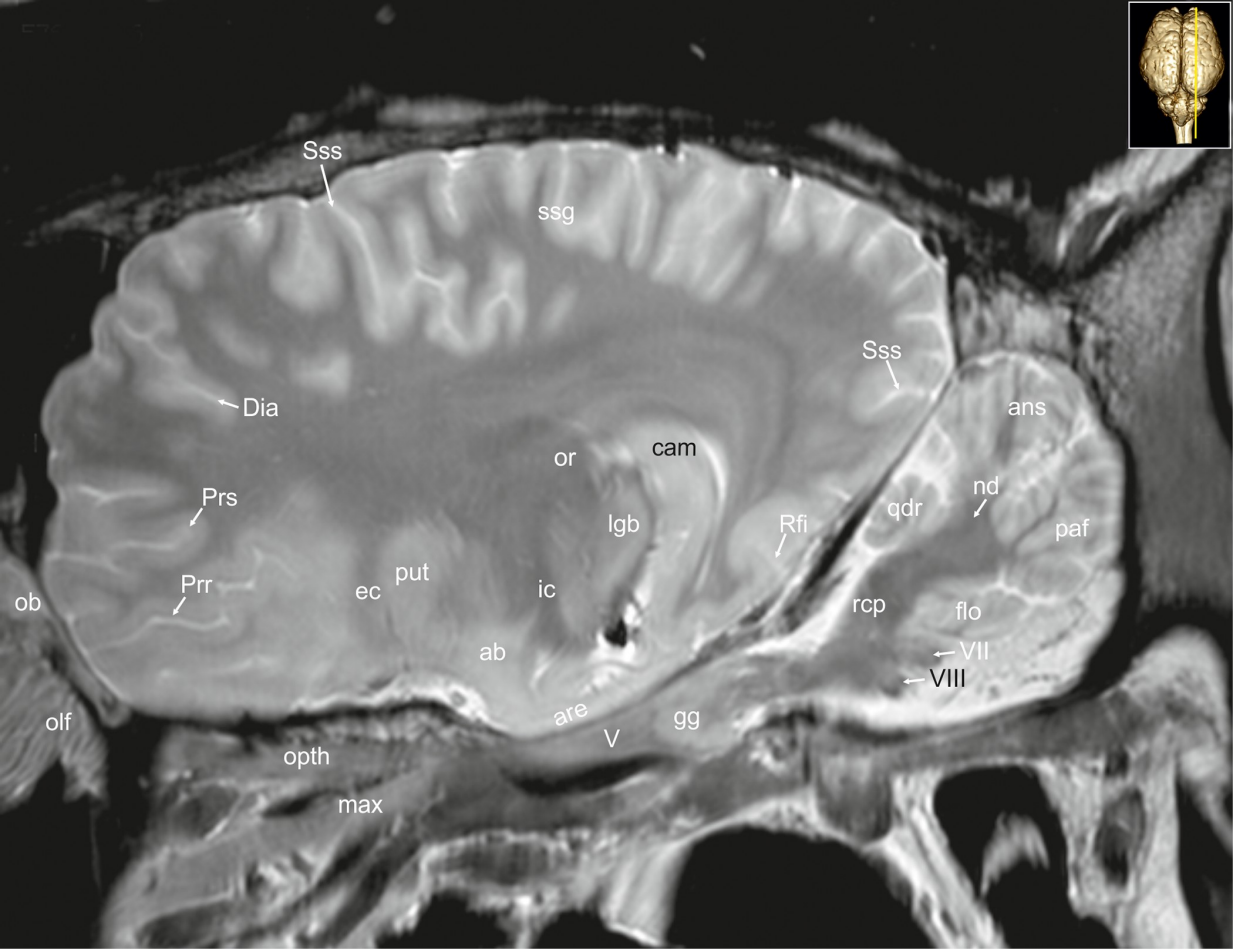
Parasagittal magnetic resonance image of the equine brain at the level of the optic radiation. ab: amygdaloid body, ans: ansiform lobule, are: entorhinal aera, cam: ammon’s horn, chp: choroid plexus, Dia: diagonal sulcus, ec: external capsule, flo: flocculus, gg: Gasserian ganglion, ic: internal capsule, lgb: lateral geniculate body, max: maxillary nerve, mgb: medial geniculate body, nd: dentate nucleus, ob: olfactory bulb, olf: olfactory fibres, opth: ophthalmic nerve, or: optic radiation, paf: paraflocculus, Prr: prorean sulcus, Prs: presylvian sulcus, put: putamen, qdr: quadrangulare lobule, rcp: rostral cerebellar peduncle, Rfi: rhinal fissure, ssg: suprasylvian gyrus, Sss: suprasylvian sulcus, V: trigeminal nerve, VII: facial nerve, VIII: vestibulocochleal nerve.

**Fig 33:**
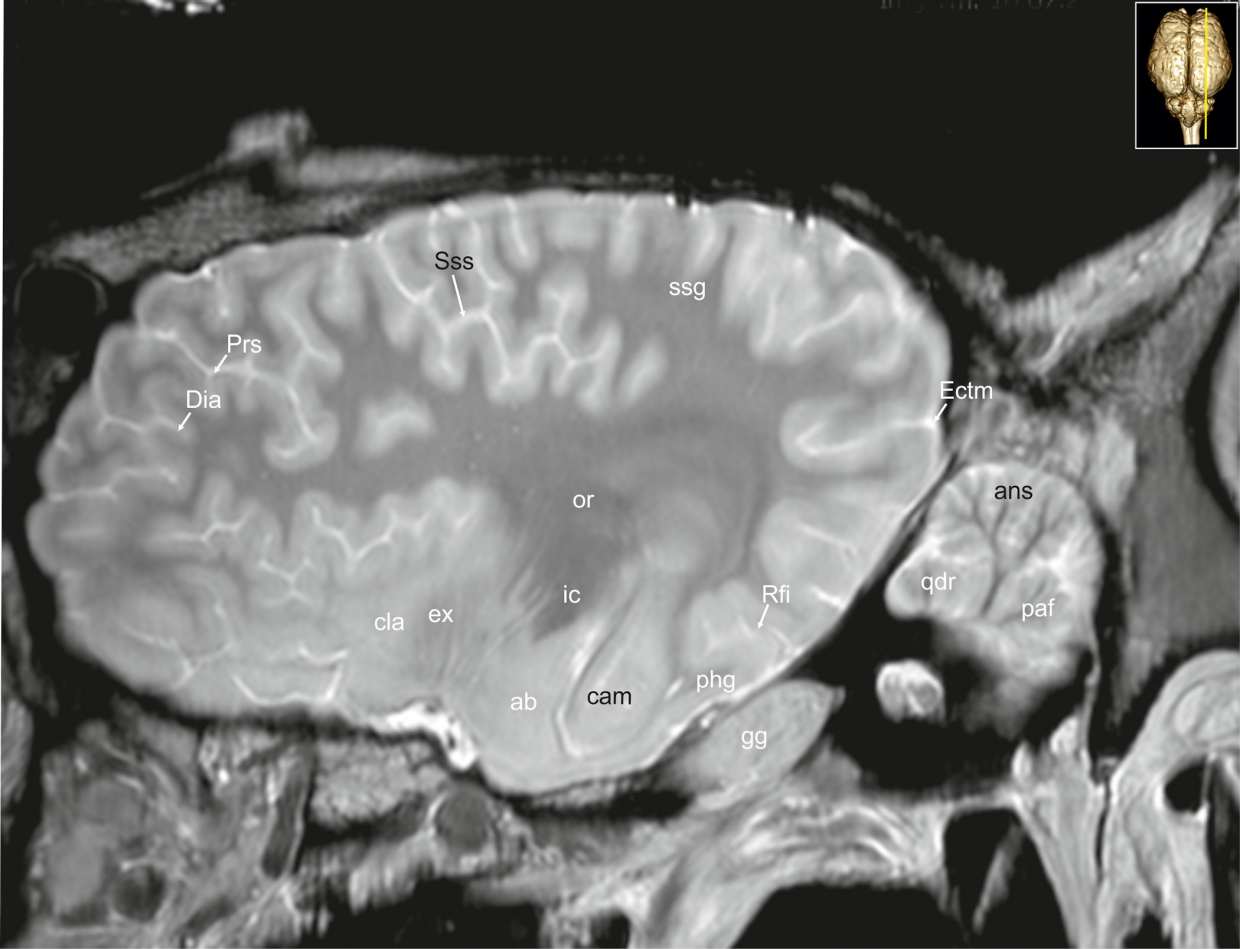
Parasagittal magnetic resonance image of the equine brain at the level of the amygdaloid body. ab: amygdaloid body, ans: ansiform lobule, cam: ammon’s horn, cla: claustrum, Dia: diagonal sulcus, Ectm: ectomarginal sulcus, ex: extreme capsule, gg: Gasserian ganglion, ic: internal capsule, or: optic radiation, paf: paraflocculus, phg: parahippocampal gyrus, Prs: presylvian sulcus, qdr: quadrangulare lobule, Rfi: rhinal fissure, ssg: suprasylvian gyrus, Sss: suprasylvian sulcus.

**Fig 34:**
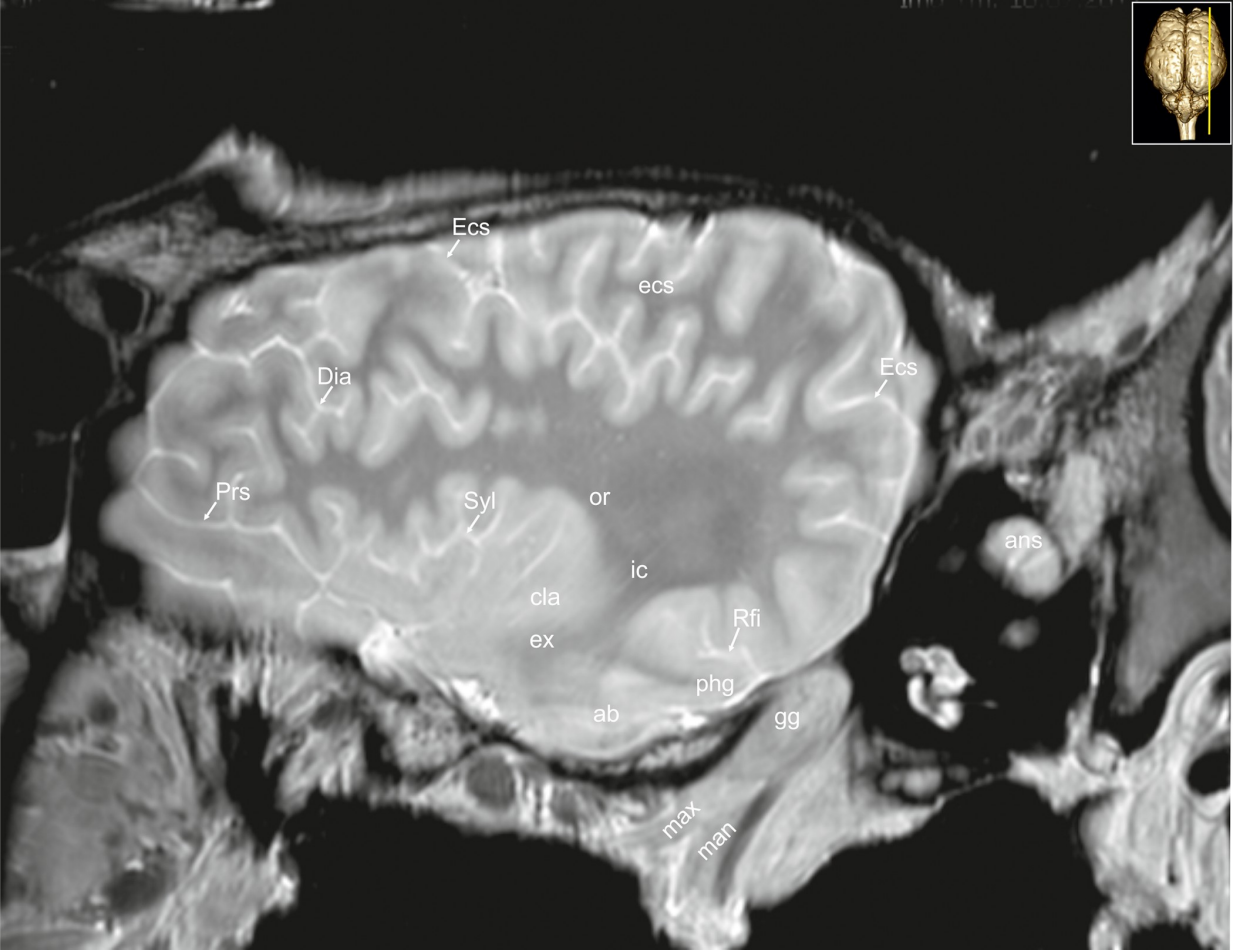
Parasagittal magnetic resonance image of the equine brain at the level of the external capsule. ab: amygdaloid body, ans: ansiform lobule, cla: claustrum, Dia: diagonal sulcus, Ecs: ectosylvian sulcus, ecs: ectosylvian gyrus, ex: extreme capsule, gg: Gasserian ganglion, ic: internal capsule, man: mandibular nerve, max: maxillary nerve, or: optic radiation, phg: parahippocampal gyrus, Prs: presylvian sulcus, Rfi: rhinal fissure, Syl: sylvian fissure.

### Telencephalon

#### Allocortex

The lateral rhinal fissure (Fissura rhinalis lateralis; Figs 1A, 2–9, 10–12, 18, 20, 21, 27 and 30–34; S1–S8 Figs: Rfi) separates the prominent rhinencephalon from the laterally adjoining telencephalon. The latter can be subdivided into a basal, septal, and a limbic part [23–25]. The rostral-most aspect of the basal part constitutes the large olfactory bulb (Bulbus olfactorius; Figs 1A–1D, 19, 20, 21, 29 and 30–32; S1 Fig: ob), which is long and flat, lying mostly underneath the frontal lobe (Fig 1A and 1B). Each has a visible oval olfactory recess of the ventricular system (Figs 2, 18 and 20; S1 Fig: olr). On the ventral aspect of the bulb, the medial (Figs 2 and 3; S2 Fig: mot) and lateral olfactory tracts (Figs 2–6, 18, 19 and 30; S1 and S2 Figs: lot) are visible as irregular hypointense bands. The lateral olfactory tract is rather thick lying within the faint endorhinal sulcus (Sulcus endorhinalis; Fig 5; S4 Fig: Enrh). Together with the lateral rhinal fissure the latter borders the shallow lateral olfactory gyrus (Gyrus olfactorius lateralis; Figs 1B, 3 and 5; S2 and S4 Figs: log), housing the prepiriform (Figs 4 and 6; S3 and S5 Figs: prpc), and more caudally the periamygdaloid cortex. Medio-ventral to the lateral olfactory tract lies the large olfactory tubercle (Tuberculum olfactorium; Figs 1B, 4, 18, 19, 29 and 30; S4 Fig: otb) ventrally adjacent to the accumbens nucleus (Nucleus accumbens septi; Figs 3, 4, 19, 20, 28 and 29; S2 and S3 Figs: acn) and the striatum.

Caudal to the olfactory tubercle on each side is the rostral perforated substance (Substantia perforata; Fig 19: pfs). It is dorsally continuous with the basal nuclei. The perforated substance is characterized by hyperintense penetrating branches of the striate arteries (ramus striatus of the medial cerebral artery), which supply the internal capsule and the basal nuclei (Fig 6: stra). Together, the olfactory tubercle and the perforated substance build the olfactory area (area olfactoria). The diagonal band of Broca (Figs 1B, 1E and 19; S4 Fig: dbb), also called the diagonal gyrus, and the diagonal sulcus (Sulcus diagonalis; Fig 5; S4 Fig: Dias), indicate the caudal transition from the olfactory area to the large piriform lobe of the rhinencephalon. As a special feature of the horse, the massive piriform lobe is grooved by faint sulci [23], of which only the lateral sagittal sulcus (Sulcus sagittalis; Figs 1A, 1B and 8; S7 Fig: Sgs) can be identified in MR-images based on comparison with histological slides. The ventral-most bulging part of the piriform lobe is the broad semilunar gyrus (Gyrus semilunaris; Figs 1B and 7; S6 Fig: slu), indicating the cortical region of the amygdaloid complex (Figs 7, 19, 20, 21 and 32–34; S6 Fig: ab). The amygdaoid complex consists of a cortical- or corticoid- and a subcortical nuclear part with numerous subnuclei, which can be differentiated based on its cytoarchitectonics and histochemistry (S6 Fig: ab) [26]. In our scans, the amygdaloid body can only be seen as a mildly hyperintense ovoid nuclear mass, which is covered ventrally by the paleocortex of the piriform lobe (Lobus piriformis; Figs 7 and 19: pir). Subnuclei are not visible in our MR-images. A slender hypointense fibre bundle runs around the thalamus and the caudate nucleus, which connects the amygdala with the hypothalamus and other basal forebrain regions. This is the terminal stria (Stria terminalis; Figs 6–8; S5–S7 Figs: stt), part of the extended amygdala.

The amygdaloid bodies are associated to the limbic part of the rhinencephalon (Pars limbica rhinencephali [23]. The other components are the cingulate (Gyrus cinguli; Figs 2, 3, 5, 6, 8, 9, 24–26 and 28–30; S2, S4 and S5 Figs: cig) and parahippocampal gyrus (Gyrus parahippocampalis; Figs 8, 9, 20, 21, 30, 33 and 34; S7 and S8 Figs: phg), the hippocampal formation including the Cornu ammonis (hippocampus proper), the dentate gyrus, and fornix, as well as the mammillary body (Corpus mamillare; Figs 18, 26 and 27: mb) and the mammillothalamic tract (Fasciculus mamillo-thalamicus; Figs 7, 18, 19, 20 and 27–29; S6 Fig: fmt). The large Ammon’s horn (Cornu ammonis; Figs 8, 9, 20, 22, 23 and 26–33; S7 and S8 Figs: cam) is seen as a curved convoluted elevation of the floor of the caudal horn of the lateral ventricle (Ventriculus lateralis; Figs 5–8, 23 and 30; S2–S7 Figs: lv) around the diencephalon. The dorsal (septal) part of the Ammon’s horn is well developed proceeding beneath the corpus callosum (Figs 1E, 7, 8, 23 and 26–29; S2–S7 Figs: cc) running in a spiral-like course around the thalamus. As part of the allocortex, the Ammon`s horn is characterized by three layers in contrast to the six-layered neocortex (isocortex) (S8 Fig). Definite identification of these layers is difficult in MR-images, however, the surface is certainly covered with a thin hypointense layer of white matter, the so-called alveus (Figs 8, 9, 21 and 23; S7 and S8 Figs: alv). The nerve fibers of the alveus converge to form the Fimbria hippocampi (Figs 8, 20 and 30; S7 and S8 Figs: fh) that form the crus of the fornix, arching ventral to the corpus callosum. The two crura then come together in the midline to form the body of the fornix (Corpus fornicis; Figs 6, 7, 21 and 22; S6 Fig: cfo). They are interconnected by fibers passing from either side representing the hippocampal commissure or commissure of the fornix (Figs 8, 28 and 29: cmf), which actually joins the two hippocampi.

The dentate gyrus (Figs 8, 9 and 21; S7 and S8 Figs: dg) is an integral part of the hippocampus. It is located medial and dorsal to the parahippocampal gyrus. The latter extends from the endorhinal fissure laterally, to the hippocampal fissure (Fissura hippocampi; Fig 8; S7 Fig: hf) medially. In transverse and dorsal images, the dentate gyrus appears as a pointed V-shaped structure apposed to the pyramidal cell layer CA1 of the Ammons’ horn (S8 Fig). The subicular complex connects the entorhinal region (S8 Fig: sub) and presubiculum. The entorhinal cortex (Figs 8, 9 and 32; S7 and S8 Figs: are) together with the presubiculum and parasubiculum are transition areas where the number of layers increases from three to six towards the entorhinal cortex and neocortex [26].

The septal region of the rhinenecphalon (Area septalis, Pars septalis rhinencephali) is seen as a thin sheet of brain tissue below the rostrum of the corpus callosum (Rostrum corporis callosi; Figs 26–28: rccl) extending ventrally to the olfactory tubercle, forming the medial wall of the rostral horn of the lateral ventricle [23]. Based on the topographical relation to the rostral commissure within the lamina terminalis (Fig 26, S5 Fig: lt) the septal area is divided into two sections. The precommissural part extends from below the rostrum of corpus callosum (subcallosal gyrus; Figs 19 and 26: scg) to the diagonal gyrus (band of Broca). It also includes the septal nuclei. The medial and lateral septal nuclei (Nuclei septales mediales, -laterals; Figs 4, 5 and 27; S3 and S4 Figs: sm; sl) are obvious in our MR-images as distinct elongated structures located at the medio-ventral border of the brain. These septal nuclei project to the habenular nuclei (Nuclei habenulares; Figs 8 and 22; S7 Fig: han) via the stria medullaris thalami (Figs 6, 7, 21, 22 and 26–29; S5 and S6 Figs: smt). This latter tract is seen as a hypointense band on the third ventricular surface of the thalamus. By way of the habenulo-interpeduncular tract the habenular nuclei project down to the interpeduncular nucleus (Nucleus interpeduncularis; Figs 10, 11 and 26; S10 Fig: ipd) ventral in the mesencephalic tegmentum.

The postcomissural part of the septal area includes the septum pellucidum (Fig 26: spe) and the columns of the fornix (Figs 7, 19, 27 and 28; S6 Fig: cf).

Above the corpus callosum is the cingulate gyrus (Gyrus cinguli; Figs 3, 5, 6, 8, 9, 24–26, 28 and 29; S3, S4 and S5 Figs: cig), which forms a dorsal cortical component of the limbic system and an integral part of Broca’s limbic lobe. It extends from the lamina terminalis rostrally, around the genu (Figs 3, 4, 22, 26 and 28; S2 and S3 Figs: gcc) and body of the corpus callosum ending just ventral to the splenium passing into the parahippocampal gyrus. It is bordered dorsally by the deep sulcus cinguli, caudally named splenial sulcus (Figs 1E, 5, 6, 8, 9, 10, 11 and 23–27; S4 and S5 Figs: Cig, Spl) [23, 27], which extends latero-ventrally in the direction of the external edge of the lateral ventricle. The white matter tract that underlies cingulate cortex projecting from the cingulate gyrus to the entorhinal cortex is referred to as the cingulum (Figs 2, 3, 5, 6, 8, 9, 24 and 28–30; S2, S4 and S5 Figs: cin) [23, 27].

#### Neocortex

**Cortical surface.** The cortex of the equine telencephalic hemisphere is intricately folded and the major sulci show a high variability in ramification [23, 24]. Even the cerebral vessels can create sulci of considerable depth, which sometimes complicate their clear identification. On the lateral surface of the hemispheres the short almost vertical sylvian fissure (Fissura sylvia; Figs 1A, 1C, 6, 7, 8, 22, 23 and 34; S3 and S5–S7 Figs: Syl) originates near the middle of the lateral rhinal fissure. Rostrally and caudally to the sylvian fissure, the ectosylvian sulcus (Sulcus ectosylvius; Figs 1A, 1C, 2, 4–10, 12, 22–25 and 34; S1, S2 and S6 Figs: Ecs) can be identified arching around the sylvian fissure. As a special feature in the horse, there is a cortical area between the sylvian and ectosylvian gyrus (Gyrus ectosylvius; Figs 1A, 4, 5, 9 and 34: ecs) referred to as the rostral and caudal oblique gyrus (Gyrus obliquus caudalis; Figs 1, 7 and 9: obl) separated by the corresponding sulcus (Figs 1A, 1C and 24: Obl) [23]. The suprasylvian sulcus (Sulcus suprasylvius; Figs 1A, 1C, 2–13, 23–25, 32 and 33; S1–S6 Figs: Sss) is positioned dorsally to the sylvian sulcus and the ectosylvian sulci. Further caudally and dorsally the marginal sulcus (Figs 1C, 5, 7, 8–10, 12–13 and 23–25; S6 Fig: Mar) can be found, flanked medially by the endomarginal sulcus (Figs 1C, 1E, 8, 10, 11, 12, 23, 24, 25, 26 and 28: Enm) and laterally by the ectomarginal sulcus (Figs 1A, 1C, 8–13, 23–25 and 33: Ectm). The ectomarginal sulcus is positioned dorsocaudally to the suprasylvian sulcus and ventrolaterally to the marginal sulcus. The endomarginal sulcus is positioned mediodorsal to the marginal sulcus.

The coronal sulcus (Figs 1A–1E, 2–4, 6, 23–25, 30 and 31; S1 and S3 Figs: Cor) emerges from dorsal margin of the hemispheres and can be connected caudomedially with the ansate sulcus (Figs 1A, 1C–1E, 4–7, 26, 28 and 29; S3, S5 and S6 Figs: Ans). Rostral to the ansate sulcus there is a small furrow on the medial side of the pallium turning to the dorsal surface, which is the cruciate sulcus (Figs 1C–1E, 2, 24, 25, 28 and 29; S1 and S2 Figs: Cru). This area contains the motor cortex of the equine brain [28]. In the rostro-lateral cortical aspect the diagonal sulcus (Figs 1A, 1D, 2, 3, 4, 21–24 and 31–34; S1–S3 Figs: Dia) crosses the lateral surface running towards the sylvian fissure. It is situated between the rostral ectosylvian and presylvian sulcus (Figs 1A, 1C, 1D, 2, 3, 21, 22 and 31–34; S1–S3 Figs: Prs), which turns rostromedially reaching the dorsal surface towards the coronal sulcus. At the base of the frontal lobe, the prorean gyrus (Figs 1A, 2, 19, 20, 22, 28, 31 and 32; S1 and S2 Figs: prr) is located between the presylvian and prorean sulcus. The latter passes around the frontal edge of the hemisphere ending on the medial side almost at the level of the genu of the corpus callosum. The presylvian sulcus merges dorsally with the coronal sulcus.

On the medial surface the faint sulcus of the corpus callosum (Fig 26: Scl) surrounds the corpus callosum rostrally, dorsally and ventrally separating the corpus callosum from the cingulate cortex above. It is framed dorsally by the cingulate gyrus that appears to be hypointense in comparison. The caudal splenial sulcus (Figs 1E, 5, 6, 8–11 and 23–27; S4 and S5 Figs: Spl) extends around the splenium of the corpus callosum (Figs 9 and 26–28; S8 Fig: scc:) and has a cranial extension over the trunk of the corpus callosum and the genu. In this aspect it is called genual sulcus (Figs 1E, 2, 3, 4, 21, 23, 26 and 27; S1–S3 Figs: Gen), which is flanked by the endogenual- (Figs 1E, 3, 4, 23, 26 and 27; S1 and S3 Figs: Eng) and ectogenual (Figs 2 and 26; S1 Fig: Ectg) sulcus. Immediately rostral to the lamina terminalis beneath the rostrum of the corpus callosum is a cortical part called the subcallosal gyrus. In the caudal aspect of the hemisphere endosplenial (Sulcus endosplenialis; Fig 26: Enspl) and suprasplenial sulcus (Sulcus suprasplenialis; Figs 1E, 9, 12 and 24–26: Sspl) flank the splenial sulcus.

#### Subpallial grey matter

The basal ganglia comprise a distributed set of brain structures in the telencephalon and diencephalon. The substantia nigra (Figs 8, 9, 10, 19 and 28–31; S7 and S8 Figs: snr) of the midbrain as well as the subthalamic nucleus (Fig 29: stn), which is located in the caudal diencephalon are both functionally connected to this system. The largest group of these nuclei is the large striate body (Corpus striatum), which is subdivided into two parts (caudate nucleus, lentiform nucleus) by fiber masses of the internal capsule (Figs 4–7, 19, 21, 22, 23, 30 and 32–34; S2–S6 Figs: ic). The caudate nucleus (Figs 3–8, 21–23, 27, 28 and 29–31; S2–S8 Figs: cn) as the dorso-medial component bulges into the central part of the lateral ventricle. It is isointense to the cerebral cortex. The lentiform nucleus comprises two parts, the putamen (Figs 3–7, 20–22 and 32; S2–S6 Figs: put), lying ventro-laterally and being associated with the globus pallidus (Figs 5, 6, 21, 30 and 31; S4 and S5 Figs: gp), separated from each other by a thin sheet of white matter. In the horse, the head of the caudate nucleus quickly tapers into the tail (cauda) and the body (corpus) is really short compared to other ungulates [17].

Stripes of grey matter connect the head of the caudate nucleus and the putamen. Further caudally, the putamen quickly increases in height and width but never attains the dimensions of the caudate nucleus. The rostral part of the putamen appears on transverse sections as a rather narrow straight band of grey matter stretching from dorso-lateral to ventro-medial. The middle part, the largest, is triangular in shape on the transverse sections. The putamen is laterally bordered by a thin layer of hypointense white matter, the external capsule (Capsula externa; Figs 4–7, 22 and 32; S2–S6 Figs: ec). A hypointense fiber bundle can be seen ventrolateral to the globus pallidus running into the caudal limb of the internal capsule. This is the ansa lenticularis (Fig 6; S6 Fig: ansl). The ansa lenticularis runs dorsally to connect the globus pallidus to the thalamus and subthalamus.

Another part of the basal ganglia is the claustrum (Figs 3–6, 8B, 22, 33 and 34: cla), which is separated from the putamen by the external capsule and from the insular cortex (Cortex insularis; Fig 5; S7 Fig: ins) by the extreme capsule (Capsula extrema; Figs 3–7, 33 and 34; S3–S6 Figs: ex). The claustrum (Figs 3–6, 8, 22, 33 and 34; S2–S5 and S7 Figs: cla) appears as a column of hyperintense grey matter located adjacent to the lateral rhinal fissure. It changes its appearance while running caudally, varying from a polymorphic star-shaped to a triangular form. The dorsal part is lying adjacent to the insular cortex (claustrum proper; claustrum insulare) and the ventral part is located next to the prepiriform cortex (also called endopiriform nucleus) [23]. Histological images (S8 Fig) reveal that on the level of the thalamus, the claustrum can be separated into different subnuclei that cannot be seen in the MR-images.

#### White matter

White matter forms the bulk of the deep parts of the cerebral hemispheres, (corpus medullare of the forebrain). Fibers coming from the cerebral convolutions converge in the dorsal hemisphere to a great mass of white matter. Dorsal to the lateral ventricles and corpus callosum, it forms the semioval centre (Centrum semiovale; Figs 3–6; S2–S5 Figs: cso). Further ventrally the white matter mass does not contain association fibers running from one part of the hemisphere to the other, but only ascending and descending fibers, together referred to as the corona radiata, give rise to the internal capsule. Seen from the dorsal view on the level of the striatum, the curved, fan-shaped internal capsule can be separated into a rostral limb (crus rostralis) between the head of the caudate nucleus and the putamen and a caudal limb (crus caudale) between the thalamus and putamen (Figs 3–7, 20, 21, 22 and 32; S2–S6 Figs: put.).

The delineation of distinct white matter fascicles is possible within the corpus medullare. A large oval to crescent shaped elongated bundle of fibers situated directly above the caudate nucleus and beneath the corpus callosum can be identified as the subcallosal fasciculus (Fascisulus subcallosus; Figs 3–9, 23 and 30; S2–S8 Figs: fsc). This bundle connects the frontal and occipital cortex with the caudate nucleus. The white matter fibers within the cingulate gyrus are together called the cingulum. These pathways connect the frontal lobe (genual gyrus, subcallosal gyrus) and the neurons of the cingulate gyrus with the enthorhinal cortex [23, 29]. The ventral (inferior) longitudinal fasciculus runs sagittally next to the subcallosal fascicle along the lateral walls of the ventral and caudal horn of the lateral ventricle [29]. According to differences in density, fiber diameter, and myelination, different layers (strata) can be differentiated (stratum sagittale externum and–internum, (Figs 10 and 11: stse, stsi) [29]. The optic radiation coming from the lateral geniculate body (Corpus genicluatum laterale; Figs 8, 9, 22 and 29–32; S7 and S8 Figs: lgb) running to the primary visual cortex along the calcarine fissure can be discerned. It is a thin pathway with moderate hypointensity compared with the adjacent radiation of the corpus callosum within the internal capsule. The pathways of the other sensory (radiation acustica, -sensoria) systems cannot be identified with certainty.

The corpus callosum (Figs 1E, 5–8, 23, 26, 27, 28 and 29; S3, S4 and S5 Figs: cc) is well developed. The fibers of the body extend laterally as the radiation of the corpus callosum (Radiatio corporis callosi; Figs 3–9, 21, 26–28 and 30; S2–S6 Figs: rcc). A phylogenetically older connection is the rostral commissure (Figs 5, 6, 20 and 26–29; S4 and S5 Figs: rc) that interconnect basal brain structures of the two cerebral hemispheres. It contains decussating fibers from the olfactory tracts that cross midline in the lamina terminalis in front of the columns of the fornix. In sagittal images the rostral commissure is a round to oval structure within the ventral lamina terminalis. In dorsal images it appears as a characteristic hypointense U-shaped stripe running ventral to the striatum.

#### Diencephalon

The diencephalon consists of five main components–epithalamus, thalamus metathalamus, subthalamus and hypothalamus (Figs 18 and 19: hyp). The largest, rostral-most part is represented by the nuclear masses of two thalami, which enclose the third ventricle. Along the midline the two large ovoid thalamic complexes are so prominent that the ventricle is restricted here to a largely circular vertical residue bordering the fused parts of the thalami (intermediate mass, interthalamic adhesion: 7; S6 Fig: ita). Within the thalamus, some distinct nuclei can be identified in MR-images based on their topographical relation to adjacent structures. The dorsal and ventral thalamic nuclei are separated by thin internal (Fig 29: lmi) and external medullary laminae (Lamina medullaris externa; Figs 7, 8, 21 and 22; S6 and S7 Figs: lme). The anterior nuclei of the thalamus (or anterior nuclear group) are a collection of neurons at the rostral end of the dorsal thalamus. The lateral and medial anterior nuclei are located between the stria medullaris and the stria terminalis. They protrude the dorsal thalamic surface into the third ventricle (Fig 7; S6 Fig: nad) [30, 31].

The hyperintense thalamic reticular nucleus (Nucleus reticularis thalami; Figs 7, 21, 22, 30 and 31; S6 and S7 Figs: nrt) forms a hyperintense capsule around the thalamus medially adjacent to the internal capsule. The elongated structure is separated from the thalamus by the hypointense external medullary lamina. Caudomedially the thalamus shows an angular prominence, the pulvinar thalami (Figs 8 and 9; S7 and S8 Figs: pul), which passes into an ovoid swelling laterally, the lateral geniculate body. It is a distinctive subdivision of the thalamus serving as a visual relay pathway. The microscopic structure of the lateral geniculate body is characterized by a series of alternating grey matter and white matter layers, which is, however, not visible in our scans. Its dorso-lateral border is sharply defined by the hypointense optic radiation (efferents from lgb) and the ventro-lateral border defined by the optic tract (afferents to lgb). Beneath the lateral geniculate body and the pulvinar, a second slightly hyperintense ovoid swelling is located, the medial geniculate body (Corpus geniculatum mediale; Figs 8, 9, 11, 21, 29 and 31; S7 and S8 Figs: mgb) that is the thalamic relay nucleus for the auditory pathway. It receives direct input from the the brachium (connecting arm) of the caudal colliculi (Figs 10, 11, 21, 30 and 31; S9 and S10 Figs: bcc) that carries auditory afferent fibers via the lateral lemniscus (Lemniscus lateralis; Figs 11, 12, 19 and 29; S10 Fig: lal). Lateral and medial in this respect is referred to the conditions in humans, in the horse these geniculate bodies are stacked vertically [23].

#### Subthalamus

The subthalamus is a region formed by several grey matter nuclei and their associated white matter structures. The subthalamic nucleus (Fig 29: stn) is located at the junction of the midbrain and hypothalamus just ventral to the thalamus. The so-called zona incerta (Figs 8, 28 and 29; S7 Fig: zi) is formed of cell groups within the ventral thalamus, which are caudo-dorsally contiguous with the reticular nucleus of the thalamus. It is located close to the midline on the floor of the transition from the diencephalon to the midbrain above the subthalamic nucleus. Medially adjacent is the fasciculus mamillo-thalamicus (Figs 7, 18–20 and 27–29; S6 Fig: fmt). It can be seen as a hypointense stripe between hyperintense regions. These regions (Area tegmentalis) are also named "H-fields" (H stands for the german word “Haube” referring to “tegmentum”). H_1_ (thalamic fasciculus) lies dorsally to the zona incerta, H_2_ ventrally to it arching over the dorsal border of the subthalamic nucleus (lenticular fasciculus) (S7 Fig: H_1_, H_2_) [30–32]. The field H3 or prerubral field cannot be reliably identified in our MR-images.

#### Hypothalamus

The hypothalamus forms the basal wall of the third ventricle; the hypothalamic sulcus (Sulcus hypothalamicus; Fig 7, S6 Fig: hs) delineates its dorsal border towards the thalamus. It extends from the rostral limit of the optic chiasm (Chiasma opticum; Figs 1B, 5, 6, 17, 26 and 27; S4 and S5 Figs: cho) to the caudal end of the mammillary bodies. It is bordered ventro-laterally by the internal capsules (Capsula interna; Figs 4–7, 19, 21, 22, 23, 30 and 32–34; S2–S6 Figs: ic), and ventro-caudally by the crura cerebri (Figs 1B, 7–10, 18, 19, 20, 22, 29, 30 and 31; S6–S8 Figs: crc). Medially, the hypothalamus is bordered by the third ventricle (Figs 7, 19, 20 and 26; S6 Fig: 3). Two distinct fiber bundles pass to the mammillary body along the ventral surface of the midbrain. These are the peduncles of the mammillary body (Pedunculi corporis mammillaris; Figs 8, 9 and 17; S7 and S8 Figs: pcm) connecting the mammillary body to the dorsal and ventral tegmental nuclei. The mamillothalamic tract arise from cells of the medial and lateral nuclei of the mammillary body and from fibres from the fornix, connecting the mammillary body to the the anterior thalamic nucleus. The hypothalamus receives afferent pathway from the amygdala (Corpus amygdaloideum; Figs 7, 19, 20, 21, 32, 33 and 34; S6 Fig: ab) via the stria terminalis.

The pituitary gland (Glandula pituitaria; Figs 1B and 7–9: pg) is a protrusion of the ventral hypothalamus. It is a large dorso-ventrally flattened mass made up of three major divisions in the horse, the adenohypophysis (Figs 8, 17, 26, 28 and 29: ah), the neurohypophysis (Figs 17 and 26: nh), and the intermediate part (pars intermedia; Figs 26 and 28: psi). The neurohypophysis has two visible parts. The distal part forms the bulk of the neurohypophysis extending caudally far beyond the level of the mammillary bodies towards the midbrain [33]. The other part is the elongated infundibular stalk (pars infundibularis; Figs 7, 17 and 26: inf), located in the middle between the two lobes of the adenohypophysis in transverse images. The ventral part of the third ventricle builds a large and wide recess extending into the infundibular stalk (infundibular recess; Fig 17: ir). The pars intermedia of the horse`s pituitary gland is large with a hypointense signal intensity. In contrast to other ungulates it completely surrounds the hyperintense infundibular process [33]. It can be clearly seen in sagittal images as a hypointense rim dorsal on the neurohypophysis. A hypophyseal cleft (cavum hypophysis) that characterizes the pituitary gland in many artiodactyls is not present [33]. Rostral to the pituitary gland the two optic nerves (Nervi optici; Figs 1B, 3, 4, 27, 28 and 30: opn) cross in the optic chiasm. From here, each optic tract (Tractus opticus; Figs 7, 8, 9, 18, 19, 20, 21, 22, 29 and 30; S7 and S8 Figs: ot) proceeds to the lateral geniculate body and via the optic radiation (Radiatio optica; Figs 8, 9, 22, 24, 31, 32, 33 and 34; S7 and S8 Figs: or) to the primary visual cortex.

#### Epithalamus

The epithalamus represents the dorsalmost and caudalmost part of the diencephalon. It comprises the habenular nuclei (Nuclei habenulares; Figs 8 and 22; S8 Fig: han), the stria medullaris thalami, the pineal body (Glandula pinealis; Figs 9, 10 and 26; S9 Fig: pb) and the caudal commissure (Commissura caudalis; Figs 9, 26 and 27; S8 Fig: cdc). The epithalamic structures are well-developed in the horse. The pineal body is large and has a lanceolated shape in sagittal images. It houses a deep pineal recess (Fig 26: rpb). Underneath the pineal body, the paired habenular nuclei are positioned, protruding into the dorsal part of the third ventricle. The habenular nuclei project to the interpeduncular nucleus of the midbrain via the habenulo-interpeduncular tract (Tractus habenulo-interpeduncularis; Figs 8, 21, 22, 27, 28, 30 and 31; S7 Fig: hit) (fasciculus retroflexus of Meynert). Between each nucleus the habenular commissure (Commissura habenularum; Figs 9 and 22; S8 Fig: cha) spans over the third ventricle. Ventral to it, the fibers of the caudal commissure also crosses midline immediately dorsal to the mesencephalic aqueduct (Aqueductus mesencephali; Figs 10, 11 and 26; S9 and S10 Figs: aq), where it becomes continuous with the third ventricle (Figs 19, 22 and 26: 3).

### Mesencephalon

The mid-brain of the horse is short, however, has a considerable diameter. In our transverse scans it is square to rectangular in shape. Ventrally it extends from behind the mammillary bodies (Corpus mammillare; Figs 18, 26 and 27: mb) to the rostral margin of the pons (Figs 1A, 1B, 17, 26, 27 and 31: po). The caudal commissure (Commissura caudalis; Figs 9, 26 and 27; S8 Fig: ccd) can be seen as the dorsal junctional landmark between the diencephalon and the mesencephalon [23]. Caudal to this point the habenulae and the posterior thalamic nuclei form the rostral aspect of the mesencephalic pretectal area (Area praetectalis; Figs 22, 28 and 29; S8 Fig: pta). Laterally the pretectal area reaches to the caudal nuclei of the thalamus. Medially, it meets the caudal commissure and the central grey substance (Substantia grisea centralis; Figs 9–11, 19 and 27; S8 and S9 Figs: cgs).

The tectum is dominated by large rostral colliculi (Colliculi rostrales; Figs 10, 22 and 26–28: roc), and the cerebral aqueduct, which is wide and high in the horse. The rostral colliculus is much larger than the caudal one (Colliculus caudalis; Figs 9, 11 and 28; S8 and S10 Figs: cdc). Alternating strata of cells and fibers give the rostral colliculus a faintly layered appearance, best seen in transverse images (Fig 10). The whole colliculus shows eight layers in histological slices (S9 Fig). The superficial zone includes the stratum zonale, stratum griseum superficialis, and stratum opticum [28, 34]. The deep zone can be divided into the stratum griseum intermedium and stratum griseum profundum each separated by a stratum album intermedium and -profundum (S9 Fig) [28, 34]. Axon bundles arising from the white matter layers of the colliculi link the two rostral colliculi along their whole rostro-caudal extent. This commissure of the rostral colliculi (Commissura colliculi rostrales; Fig 10: ccr) can be seen as a hypointense stripe at the medial basis between the rostral colliculi. At the lateral basis of the colliculus a flat hypointense fibre bundle leave the optic tract medial to the pulvinar to enter the pretectal area and the rostral colliculus. This band is slightly rising over the surface forming the brachium of the rostral colliculus (Brachium colliculi rostralis; Figs 28 and 29: bcr).

The caudal colliculus (Colliculus caudalis; Figs 9, 11 and 28; S10 Fig: cdc) has a rather homogenous compact central nucleus (Fig 11: nca), which is distinctly hyperintense [35]. Fiber bundles arise from that nucleus, cross the midline (commissure of the caudal colliculi; Figs 11, 26 and 27: ccc), and end in the contralateral colliculus. Just below the caudal colliculus and the central lobe of the cerebellum (Figs 11, 12, 21–23, 26 and 27: cl) trochlear nerve fibers cross midline (decussation of the trochlear nerve; Figs 21, 26 and 27; S10 Fig: dtn). Where these fibers meet the caudal colliculi, the crescent shaped parapeduncular nuclei flank the rostral cerebellar peduncle where it enters the midbrain tectum [23, 34]. The brachium of the caudal colliculus passes forward and upward from the caudal colliculus and disappears beneath the medial geniculate body.

The sulcus limitans in the mesencephalic aqueduct seperates the tectum from the ventral tegmentum of the midbrain (Fig 14; S9, S12 and S13 Figs: slm). Long projection fibers enter and exit the prosencephalon mainly via the internal capsule, to continue into the mesencephalon as the crura cerebri. Crura cerebri and nuclei of the mesecenphalic tegmentum constitute the pedunculi cerebri. At the level of the rostral pons, the crural fibers perforate the later and either end in the pontine nuclei or continue into the rostral medulla. The cerebral peduncles accomodate the large substantia nigra (Figs 8, 9, 10, 19 and 28–31; S7 and S8 Figs: snr). A pars reticulata and pars compacta (S8 Fig), which lies medial to the pars reticulata cannot be discerned in our MR-images. The white matter bundles are rather flat and constitute the external parts of the peduncles. The bulk of the white matter bundles divide into a flat ventral part and a rounded (level of habenular nuclei) to triangular dorsolateral part (level of the caudal commissure).

The elongated red nucleus (Nucleus ruber; Figs 9 and 27; S8 Fig: rn), located dorsomedially to the substantia nigra within the tegmentum, appears only slightly hyperintense. Ventro-medially between the red nuclei, the decussation of the rostral cerebellar peduncles is obvious (Decussatio pedunculorum cerebellarium rostralium; Figs 10, 20 and 26–28: drp). Rubrospinal fibers that originate in the red nucleus cross the midline immediately after exiting the nucleus (Decussatio tegmentalis dorsalis, sive Decussatio nuclei ruberis: decussation of the red nucleus; Fig 27: dtd). Then they course dorsally running beneath the rostral cerebellar peducle in the midbrain deviating laterally in the medulla as the rubrospinal tract (Tractus rubrospinalis; Figs 12–14, 18, 21 and 28; S10, S12 and S13 Figs: rst) [23, 35].

The medial longitudinal fasciculus is caudally continuous with the fasciculus proprius of the spinal cord [23]. It runs close to midline on the ventral aspect of the cerebral aqueduct. Close to the location, where the crus enters the forebrain, the optic tract winds around its ventrolateral surface in an oblique direction on its way to the lateral geniculate body. The area between the crura is termed the interpeduncular fossa, which is pierced by small blood vessels (caudal perforated substance; Fig 26 not labeled). The interpeduncular nucleus is a hyperintense unpaired cell group at the base of the tegmentum between the cerebral crura.

The midbrain is longitudinally traversed by the mesencephalic aqueduct that connects the fourth with the third ventricle. The hyperintense signal around the mesencephalic aqueduct corresponds to the periaqueductal grey matter (Substantia grisea centralis; central grey substance), which is formed by neurons that form a caudal continuation of the diencephalic periventricular nuclei of the hypothalamus. These neuronal masses surround the aqueduct and continue throughout the midbrain. Its shape, as seen in transverse sections, varies at different levels. Scattered throughout the central grey substance are numerous nuclei, which are collectively called the tegmental nuclei. Besides these scattered nuclei the central grey substance contains the nuclei of the oculomotor (Nucleus nervi oculomotorii; Fig 10: nom) and trochlear nerves (Nucleus nervi trochlearis; Fig 11; S10 Fig: nto), and the nucleus of the mesencephalic root of the trigeminal nerve (Nucleus tractus mesencephalicus nervi trigemini, S10 Fig: nmt). Between the fasciculus longitudinalis medialis (Figs 10–15, 19, 20 and 26–28; S9–S14 Figs: flm) and the decussation of the rostral cerebellar peduncles is an area of hyperintense signal corresponding to the oculomotor nucleus. The small oculomotor nerve exits the midbrain ventrally running rostrally towards the orbital fissure.

### Metencephalon

#### Cerebellum

The shape of the large cerebellum is square in sagittal and almost triangular in the transverse planes. Transverse fissures divide the cerebellum into lobes and smaller lobules that are highly irregularly arranged. The equine vermis (Fig 1C: ver) comprises the complete set of lobules found in other mammals [24, 25, 36, 37]. The primary fissure (Fissura prima; Figs 23 and 26–29: Fp) represents the border between the rostral and caudal lobe. The rostral lobe of the vermis is larger than the caudal lobe. This relation is also reflected by the larger rostral trunk of the medullary body (Corpus medullare cerebelli). This is somewhat unique in the horse in contrast to other ungulates that have larger caudal lobes [14, 15, 36–38]. The medullary branches help to identify the subdivisions of the vermis in sagittal images. From the medullary body, three major branches of white matter take their origin. The rostral-most enters the central lobe (lobule II and III), the second almost vertical branch passes into the culmen (Lobule IV and V; Figs 11–13 and 22–28: cu). The caudal branch spreads into the declive (“lobule VI”; Figs 14, 24, 26 and 28: de), tuber (“lobule VIIB”; Figs 15, 21–24 and 26: tu), folium (“lobule VIIA”; Fig 26: fol), pyramis (“lobule VIII”;), and uvula (“lobule IX”; Figs 15, 16, 26 and 27). The nodulus (“lobule X”, Figs 26–29: no) and lingula (“lobule I”; Figs 13, 14, 20, 21, 26 and 27: li) derive their white matter directly from the medullary center [38].

The caudal vermis of the horse shows a characteristic S-shaped deviation of the cerebellar pyramis, tuber and folium (Fig 15). This can also be found in other ungulates and carnivores, but seems to have reached an extreme in the horse [24]. It is interesting to note that in some images the medullary branches within the tuber and/or folium seem to end without a cortical covering (Figs 26 and 28–30; marked by an asterisk), which has also been described in the pig but not in other ungulates. [39, 40].

The cerebellar hemispheres are dominated by the massive ansiform (meaning loop-like) lobule (Lobulus ansiformis; Figs 1A, 1C, 13–15, 22–24, 29, 30 and 31–34: ans), which is the hemispheric extension of the vermal folium and tuber [23, 38]. Unlike in other mammals, this lobule does not form a horizontal semicircle on its dorsal surface, but rather a straight vertical lobe running more or less parallel to the vermis. Shortly before the caudal end, it turns ventrally forming a lateral deviation resembling a vertical loop. The dorsal part of this loop borders a small paramedian lobule (Lobulus paramediuanus; Figs 1C, 14, 15, 22, 23, 29 and 31: pml) laterally. The hemispheric portions of the culmen consist of the comparably large quadrangular lobe. Also the large flocculus (Figs 1, 13, 14 and 30–32: flo) and paraflocculus (Figs 1A, 13, 14, 22–24, 32 and 33: paf) stand rather upright than projecting outwards, which contributes to the rectangular appearance of the equine cerebellum in transverse images. The flocculus is the bilateral extension of the vermian nodulus into the cerebellar hemispheres (also together referred to as the flocculonodular lobe). The flocculonodular lobe receives major afferents from the vestibular nuclei via the caudal cerebellar peduncle (Pedunculus cerebellaris caudalis; Figs 13, 14, 15, 29 and 30; S13 Fig: ccp).

In dorsal and transverse images, the mildly hyperintense cerebellar nuclei can be seen, embedded in the hypointense white matter. The fastigial nuclei (Nuclei fastigei; Figs 14, 22, 26 and 27: nf) lie directly dorso-lateral to the fastigial recess of the fourth ventricle, whereas the dentate nucleus (Nucleus dentatus; Figs 22, 29 and 30–32: nd) is located more ventro-laterally in the cerebellar hemisphere. Between these two lies the interpositus nucleus (Nucleus interpositus; Figs 14, 22, 28 and 29: nip), which is composed of two subnuclei (globose and- emboliform nucleus) [38]. Although surrounded by the intensively hypointense medullary body of the cerebellum, the cerebellar nuclei cannot be well visualized.

A large bundle of hypointense fibers originate from the dentate and interpositus nuclei running along the lateral wall of the fourth ventricle in the rostral direction. This bundle is the rostral cerebellar peduncle. Most of these fibers cross in the mesencephalic tegmentum (decussation of the rostral cerebellar peduncles) ending in the contra-lateral red nucleus and the ventral thalamus. The caudal lobe has extensive cortico-pontocerebellar connections via the middle cerebellar peduncles (Figs 11, 12 and 18–21; S11 and S13 Figs: mcp) [23, 25].

#### Medulla

The most characteristic feature of the brainstem is the massive metencephalic pons building the transition from mesencephalon to the medulla oblongata. It is only moderately developed in the horse. It consists of transversely arranged fiber bundles, interspersed with collections of mildly hyperintense pontine nuclei (Nuclei pontis; Figs 11, 12, 28 and 29; S10 and S11 Figs: npo). Cortico-pontine fibers descend from cerebral cortex towards the pontine nuclei, decussate as the transverse fibers of the pons (Figs 11, 27, 29 and 30; S10 Fig: tfp) entering the contralateral cerebellum as the medial cerebral peduncles, which bound the pons laterally. Linear bands of low signal correspond to ascending and descending tracts passing through the pons. The ascending fibers of the medial lemniscus (Lemniscus medialis; Figs 8–12, 17–20, 27 and 28; S7, S8, S10 and S11 Figs: ml) delineate the ventral part of the pons (basis pontis) and the dorsal part of the pons (Tegmentum pontis). The tegmentum of the pons is continuous with the mesencephalic tegmentum and the reticular formation of the medulla. The continuation of the deep arcuate fibers run to the ventral medulla where they cross to the other side (decussation of medial lemniscus; Fig 17: dcml). They proceed rostrally as the paired medial lemniscus through the pons and later the dorsolateral brainstem to the ventral nuclei of the thalamus.

Corticospinal tract fibers (Tractus corticospinalis; Figs 12, 17 and 29; S10, S11 and S15 Figs: cst) traverse the pons caudally forming the pyramidal tracts (Figs 13–16, 26, 28 and 29; S12 and S15 Figs: pyr), which are recognizable on each side along the ventral midline. The pyramidal tracts are only small and flat in the horse [41]. At the most caudal pole of the pyramids the cortico-spinal axons cross over the midline and now continue their descent on the contralateral side (Decussatio pyramidalis; Fig 28: dpy).

The sensory and motor divisions of the trigeminal nerve emerge as separate roots (radix motoria, radix sensoria) from the ventrolateral pons. The trigeminal ganglion (Gasserian ganglion; Figs 12, 18, 19, 20 and 32–34: gg) is a great sensory ganglion of the trigeminal nerve. It receives sensory neurons of all three branches of the ophthalmic, mandibular, and maxillary divisions (Figs 6–8, 17, 32 and 34: max), forwarding sensory input to the sensory root. The bulk of afferent fibers descend united to the trigeminal spinal tract (Tractus spinalis nervi trigemini; Figs 13–16, 18, 19 and 30; S12–S15 Figs: tsnt) that is situated just below the lateral surface of the medulla. It is dorso-laterally covered by the middle cerebellar peduncle. They project towards a longitudinal cell column medial to the tract, the nucleus of the trigeminal spinal tract (Nucleus tratctus spinalis nervi trigemini; Figs 14–16, 18, 19 and 30; S12–S15 Figs: ntsn). From here, ascending fibers cross midline, mingle with the medial lemniscus and travel towards the thalamus and somatosensory cortex [42–44].

Some afferent axons from the mandibular subdivision do not enter the trigeminal spinal tract but project rostrally towards the mesencephalic trigeminal nucleus (Nucleus mesencephalicus nervi trigemini, S9 Fig: nmt). This nucleus covers in a shell-like manner the most dorsolateral part of the periaqueductal grey matter. Mesencephalic trigeminal neurons are considered centrally displaced ganglion cells, exclusively responding to stretch in the muscles of mastication [23].

The mandibular division (Ramus mandibularis nervi trigemini; Figs 31 and 34: man) of the trigeminal nerve contains sensory and motor fibers. The motor nucleus (Fig 29: mtn) of the trigeminal nerve can be found in the mid-pons.

Caudal to the pons the mildly hyperintense nucleus of the facial nerve (Nucleus nervi facialis; Figs 13 and 18; S12 Fig: nnf) can be seen, situated in the ventro-lateral pontine tegmentum. Strongly hypointense fibers originating from that nucleus take a remarkable course, traveling dorsally and looping around the hyperintense nucleus of the abducens nerve (Nucleus nervi abducentis; Figs 13, 20 and 28; S12 Fig: nab). This curve of the internal fibers of the facial nerve are called the genu nervi facialis (Figs 13, 20 and 28; S12 Fig: gnf). The fibers of the genu bulge the floor of the fourth ventricle creating the colliculus of the facial nerve. The axons then travel ventrally to exit the ventral pons lateral to the spinal tract of the trigeminal nerve (tractus spinalis nervi trigemini). The facial nerve proceeds upwards and exits the brainstem laterally (root of the facial nerve: S12 Fig: rnf).

The vestibulocochlear nerve enters the brain stem at the cerebellopontine angle dorsolateral to the emergence of the facial nerve. Its cochlear fibers synapse in dorsal and ventral cochlear nuclei (Figs 13; S12 and S13 Figs: ncv) that are located in the laterally protruding acoustic tubercle (tuberculum acusticum, Fig 14; S13 Fig: tac). This prominence underneath the caudal cerebellar peduncle is flat and rounded in the horse. From here the fibers decussate through the trapezoid body, then ascend in the lateral lemniscus (Lemniscus lateralis; Figs 11, 19 and 29; S10 and S11 Figs: lal) running to the brachium of the caudal colliculus (Brachium colliculi rostralis; Figs 10, 11, 21, 30 and 31; S9 and S10 Figs: bcc). The trapezoid body (Corpus trapezoideum; Figs 1B, 13, 14, 17, 27 and 29; S12 and S13 Figs: tb) is made up of commissural fibers (decussation of the trapezoid body; Figs 14 and 17; S13 Fig: dctb), which originate from either the ventral cochlear nucleus or nucleus of the trapezoid body (Nucleus cochlearis ventralis; Figs 13 and 14; S12 and S13 Figs: ndct), which is also called rostral (superior) olivary nucleus. It can be seen behind the pons as a small transverse ridge.

The caudal continuation of the periaqueductal grey matter is the periventricular grey matter. In its caudal extension through the medulla, numerous nuclear groups can be seen. The topographical relationship between cranial nerve nuclei and the sulcus limitans (Fig 14; S9, S12 and S13 Figs: slm) can serve as a useful landmark in identification of these structures. The lateral sulcus limitans of the fourth ventricle separates the alar and basal plates of the embryonic brain and spinal cord. The derivatives from the alar plate (sensory) lie dorsal or lateral to the sulcus limitans and derivatives from the basal plate (motor) lie ventral or medial to it, which resembles the relationship in the spinal cord.

The medial (Figs 13, 14, 20 and 28; S13 Fig: nvm) and lateral vestibular nuclei (Nuclei vestibulares mediales, -laterales; Figs 13 and 14: S12 and S13 Figs: nvl) are a group of neurons laterally flanking the fourth ventricle. They can be seen as hyperintense areas dorso-lateral to the sulcus limitans and dorsal to the internal fibers of the facial nerve. On the level of these nuclei, a hyperintense band runs vertically from the median sulcus of the fourth ventricle to the trapezoid body. This slender accumulation of cells are the medial raphe nuclei (Fig 14, S13 Fig: nrm) the tegmental part of the periventricular grey matter.

Further caudally afferent nerve fibers from the viscera unite in the solitary tract (Tractus solitarius; Figs 16; S14 and S15 Figs: sol) carrying information to the nucleus of the solitary tract (nucleus tractus solitarii, Fig 16; S15 Fig: nsol). The tract can be seen as a hypointense spot at the ventrolateral end of the hyperintense nucleus. The cell bands gradually turn medially, meet in midline and close the fourth ventricle. The connection between the two nuclei is the obex, which marks the spino-medullary transition (Fig 26; S15 Fig: obx).

Just rostral to the obex, the nucleus of the solitary tract is bordered medially by the slender motor nucleus of the vagus nerve (Fig 16, S15 Fig: vgn), followed by the conspicuous hypoglossal nucleus (Nucleus nervi hypoglossi; Figs 15 and 16; S14 and S15 Figs: hypn). The nucleus of the soliary tract is dorsally bordered with the nucleus gracilis and nucleus cuneatus.

Ventrally within the reticular formation of the medulla an elongated nuclear column contains motor neurons associated with three cranial nerves, the branchiomotor glossopharyngeal, -vagus and accessory nerve [35]. This is the ambiguus nucleus (Nucleus ambiguus; S13 Fig: amb). The most striking nucleus of the medulla is the inhomogenously hyperintense caudal (inferior) olivary nuclei. It consists of a convoluted band of cells that presents itself as a characteristic serpentine profile. It is located dorsolateral to the pyramid. Axons arising from the nucleus ambiguus pass laterally and slightly ventrally to exit the medulla at the level of the caudal olivary nucleus (Nucleus olivaris; Figs 15–17 and 27–30; S14 and S15 Figs: oli).

### Discussion

In contrast to the number of atlases in small animals, to our knowledge there is currently no comparable morphological study in the horse. The present paper is intended to serve as a broad introduction to equine brain morphology. Using a 3 Tesla MR system, images with high spatial resolution and contrast were acquired and described for the use in large animal neurology. However, some factors limit the scope and applicability of the present investigation. In this study, we described one individual horse without taking intraspecies variation due to breed, age, sexual dimorphism, reproductive cycle and other confounding factors into account. It is therefore vital to scan more individuals to reflect the anatomy of the majority of individuals in further studies. To achieve sufficient image quality and to provide morphological detail we have used a 1.5-hour scan time per plane. The resolution of most clinical scanners, which can accommodate horses might be lower and and it is unlikely that MR-images with a comparable resolution and contrast can be produced in a routine in-vivo examination. However, detailed images will improve the diagnosis and understanding of equine central nervous system diseases, especially those affecting distinct structural or functional parts [45] and may also help to establish functional MRI in equine neuroradiology [46].

In general, the equine brain reflects the mammalian blueprint. The characteristics of the ungulate brain incuding the impressive expansion of the neocortex and it`s intensive gyrification are obvious in the scans. The pattern of the equine gyri and sulci is by far more complex than in carnivores and in other ungulates. It has been shown that the level of gyrification in mammals is associated with the increase in body mass [47–50]. However, when species of similar brain weights were compared ungulate brains were significantly more gyrencephalic [51]. It has been proposed that next to the allometric expansion, ungulate-specific sulci have developed in association with special senses of the animals. One important example is the diagonal sulcus, which includes the somatosensory area of the cortex. Sensory information from the lips which are an important explorative organ is processed in the cortex surrounding the diagonal gyrus. Nostrils and tongue are widely represented in the area rostral to the suprasylvian sulcus. [42, 44]. The scaling of specialized areas in the cortex also has an indirect impact on the whole brain volume as areas involved in associative processes containing a somatosensory component may also increase.

Due to expansion of the temporal lobe in horses, the insular cortex is is deeply hidden in the depth of the sylvian- and oblique sulcus (opercularisation), and is no longer visible from the outer surface [52]. Little is known about other functional cortical fields in the horse. It is unclear whether the development of the oblique sulcus and gyrus can be seen as a result of a specialized perceptual processing demand or merely based on the evolutionary trend of increased volume of the temporal lobe [53].

The horse has been referred to as being a macrosmatic mammal [23]. Interestingly there is a certain discrepancy between the size of the large rhinencephalon and the rather small olfactory bulbs. In other ungulates as the pig and cow, the linkeage between the two structures is stronger [16–18, 40]. The same holds for the hippocampus and parahippocampal gyrus that appear small in relation to the massive rhinecephalon. Detailed allometric investigations on the brain and its parts could solve the riddle between volume and function [54, 55]. It is uncertain, as to whether this discrepancy is due to a phylogenetic trend for the volume of the allocortex (hippocampus) to decrease in association with volume expansion of the neocortex [56] or if the structures develop independently.

The descending tracts from the motor and premotor region in the cortex running to the internal capsule and continue to form the cerebral peduncles are not well developed. The pyramids are comparatively small [47]. The bulk of the tracts in the peduncles is rather expansive and flattened in the medioventral part. The thickest part runs dorsolaterally on the brainstem. Minimal motor control of the distal limbs in horses probably account for this comparatively underdeveloped efferent system [41].

The shape of the equine cerebellum has been described as beeing very chracteristic amongst ungulates [37, 38]. In general, one can see a vertical orientation rather than lateral expansion. A characteristic feature of the equine cerebellum is the deviation of the vermis. There seems to be no rule as to why this shape actually develops. Bolk [38] describes a relationship between the bend of the tuber, folium and declive of the vermis (together forming the median cerebellar lobe) and development and growth of the ansiform lobule (Figs 11–16 and 21–27). He proposes that the sharper the bend of the vermis the smaller is the ansiform lobule. The growth of the tuber results in lateral deviation of the caudal part of the vermis causing an S-shaped bend [38]. A striking feature of the cerebellum are the blind ending medullary fascicles. This was first observed in histological examinations of the pig’s cerebellum [57]. It was proposed to be a developmental impediment, thus creating a mechanical barrier due to a lack of space or compression through blood vessels. It was also described to be a physiological cortical aplasia [23, 58, 59]. Although there are also descriptions of this suspected aplasia in anatomical studies of other species, we propose this finding as to be an artifact that it is probably due to the sharp turns of the foliae out of the sagittal plane, which give the impression of a blind ending of the medullary branches. Other imaging planes do not confirm the presence of this supposed aplasia.

## Supporting information

S1 Table Abbreviations list. (DOCX)

S1 Fig Transverse histological image of the equine brain on the level of the olfactory peduncle. cin: cingulum, cig: cingulate gyrus, Cor: coronal sulcus, cor: coronal gyrus, Cru: cruciate sulcus, Dia: diagonal sulcus, Ectg: ectogenula sulcus, Ecs: ectosylvian sulcus, Gen: genual sulcus, lot: lateral olfactory tract, mot: medial olfactory tract, olr: olfactory recess, Prr: prorean sulcus, prr: prorean gyrus, Prs: presylvian sulcus, Rfi: rhinal fissure, Sss: suprasylvian sulcus.
(TIF)

S2 Fig Transverse histological image of the equine brain at the level of the genu of the corpus callosum. acn: accumbens nucleus, cc: colossal commissure, cin: cingulum, cla: claustrum, cn: caudate nucleus, cor: coronal gyrus, Cor: coronal sulcus, Cru: cruciate sulcus, cso: centrum semiovale, Dia: diagonal sulcus, ec: external capsule, Ecs: ectosylvian sulcus, fsc: subcallosal fasciculus, gcc: genu of the corpus callosum, Gen: genual sulcus, ic: internal capsule, log: lateral olfactory gyrus, lot: lateral olfactory tract, lv: lateral ventrikel, mot: medial olfactory tract, Prs: presylvian sulcus, put: putamen, Rfi: rhinal fissure, Scl: sulcus of corpus callosum, ssg: suprasylvian gyrus, Sss: suprasylvian sulcus.
(TIF)

S3 Fig Transverse histological resonance image of the equine brain on the level of the septal nuclei. acn: accumbens nucleus, Ans: Ansate sulcus, cla: claustrum, cig: cingulate gyrus, cin: cingulum, cn: caudate nucleus, Cor: coronal sulcus, cso: centrum semiovale, ec: external capsule, Ecs: ectosylvian sulcus, Eng: endogenual sulcus, ex: extreme capsule, fsc: subcallosal fasciculus, gcc: genu of the corpus callosum, Gen: genual sulcus, ic: internal capsule, lot: lateral olfactory tract, mot: medial olfactory tract, otb: olfactory tubercle, put: putamen, rcc: radition of corpus callosum, Rfi: rhinal fissure, sl: lateral septal nuclei, sm: medial septal nuclei.
(TIF)

S4 Fig Transverse histological image of the equine brain on the level of the diagonal band of Broca. cc: callosal commissure, cho: optic chiasm, cig: cingulate gyrus, cin: cingulum, cla: claustrum, cn: caudate nucleus, cso: centrum semiovale, dbb: diagonal band of broca, Dias: diagonal sulcus (Rhinencephalon), ec: external capsule, ecs: ectosylvian gyrus, Ecs: ectosylvian sulcus, Enrh: endorhinal sulcus, ex: extreme capsule, fsc: subcallosal fasciculus, gp: globus pallidus, ic: internal capsule, icl: islands of Calleja, ins: insular cortex, log: lateral olfactory gyrus, lot: lateral olfactory tract, lv: lateral ventricle, otb: olfactory tubercle, put: putamen, rc: rostral commissure, rcc: radiation of corpus callosum, Rfi: rhinal fissure, Scl: sulcus of corpus callosum, sl: lateral septal nuclei, sm: medial septal nuclei, Spl: splenial sulcus, Sss: suprasylvian sulcus, Syl: Sylvian fissure.
(TIF)

S5 Fig Transverse histological image of the equine brain on the level of the rostral commissure. Ans: ansate sulcus, ansl: ansa lenticularis, cc: corpus callosum, cho: optic chiasm, cig: cingulate gyrus, cin: cingulum, cla: claustrum, cn: caudate nucleus, cso: supraoptic commissure, ec: external capsule, Ecs: ectosylvian sulcus, ectosylvian gyrus, ex: extreme capsule, fsc: subcallosal fasciculus, gp: globus pallidus, ic: internal capsule, log: lateral olfactory gyrus, lot: lateral olfactory tract, lv: lateral ventricle, Mar: marginal sulcus, mar: marginal gyrus, prpc: prepiriform cortex, put: putamen, rc: rostral commissure, rcc: radiation of corpus callosum, Rfi: rhinal fissure, Scl: sulcus of corpus callosum, smt: stria medullaris thalami, Spl: splenial sulcus, Sss: suprasylvian sulcus, stt: terminal stria, Syl: sylvian fissure, syl: sylvian sulcus, tl: terminal lamina.
(TIF)

S6 Fig Transverse histological image of the equine brain hemisphere on the level of the amygdala. ab: amygdaloid body,alv: alveus, Ans: ansate sulcus, cc: corpus callosum, cin: cingulum, cig: cingulate gyrus, cf: column of fornix, cfo: corpus of fornix, cn: caudate nucleus, crc: cerebral crus, ec: external capsule, Ecs: ectosylvian sulcus, ecs: ectosylvian gyrus, ex: extreme capsule, fmt: mammilo-thalamic fasciculus, fsc: subcallosal fasciculus, hs: hypothalamic sulcus, ic: internal capsule, ita: interthalamic adhesion, lme: external medullary lamina, lv: lateral ventricle, Mar: marginal sulcus, mar: marginal gyrus, nad: nucleus anterior dorsalis thalami, nrt: reticular nucleus of the thalamus, Obl: oblique sulcus, obl: oblique gyrus, pfc: piriform cortex, put: putamen, rcc: radiation of corpus callosum, Rfi: rhinal fissure, slu: gyrus semilunaris, smt: stria medullaris thalami, ssg: suprasylvian gyrus, Sss: suprasylvian sulcus, stt: terminal stria, syl: sylvian gyrus, Syl: sylvian fissure, 3: third ventricle.
(TIF)

S7 Fig Transverse histological image of the equine brainat the level of the hippocampus. alv: alveus, are: entorhinal area, cam: cornu amonis, cc: corpus calosum, cla: claustrum, cn: caudate nucleus, crc: cerebral crus, dg: dentate gyrus, fh: fimbria of the hippocampus, fsc: subcallosal fasciculus, H1: fields of Forel 1, H2: Fields of Forel 2, ha: habenula, han: habenular nuclei, hf: hippocampal fissure, hit: habenulo-interpeduncular tract, ins: insular cortex, lgb: lateral geniculate body, lme: external medullary lamina, lv: lateral ventricle, mgb: medial geniculate body, ml: medial lemniscus, nrt: reticular nucleus of the thalamus, or: optic radiation, ot: optic tract, pcm: peduncles of the mammillary body, pul: pulvinar nuclei, Rfi: rhinal fissure, Sgs: sagittal sulcus, snr: substantia nigra, sub: subiculum, stt: terminal stria, Syl: sylvian fissure, zi: zona incerta.
(TIF)

S8 Fig Transverse histological image of the equine brain on the level of the caudal commissure. are: entorhinal area, alv: alveus, cam: ammon’s horn, CA1: cornu ammonis field 1, CA2: cornu ammonis field 2, CA3: cornu ammonis field 3; CA4: cornu ammonis field 4, cdc: caudal colliculus, cgs: central grey substance, cha: habenular commissure, cn: caudate nucleus, crc: cerebral crus, df: dentate fascia, fh: fimbria of the hippocampus, flv: ventral longitudinal fasciculus, fsc: subcallosal fasciculus, lgb: lateral geniculate body, mgb: medial geniculate body, ml: medial lemniscus, or: optic radiation, ot: optic tract, pb: pineal body, pcm: peduncles of the mammillary body, prs: presubiculum, pta: pretectal area, pul: pulvinar nuclei, Rfi: rhinal fissure, rn: red nucleus, scc: splenium of corpus callosum, scmo: subcommissural organ, snrc: pars compacta of the substantia nigra, snrr: pars reticularis of the substantia nigra, sub: subiculum, vtc: ventral tegmental commissure.
(TIF)

S9 Fig Transverse histological image of the equine brain on the level of the rostral colliculi. aq: mesencephalic aqueduct, bcc: brachium of the caudal colliculus, cgs: central grey substance, ctt: central tegmental tract, nmt: mesencephalic nucleus of the trigeminal nerve, pb: pineal body, slm: sulcus limitans.
(TIF)

S10 Fig Transverse histological image of the equine brain on the level of the caudal colliculi. aq: mesencephalic aqueduct, bcc: brachium of the caudal colliculus, cdc: caudal colliculus, cst: corticospinal tract, dtn: decussation of the trochlear nerve, flm: medial longitudinal fasciculus, ftp: transverse fibres of the pons, ipd: interpeduncular nucleus, lal: lateral lemniscus, mcp: medial cerebellar peduncle, ml: medial lemniscus, nII: nucleus of lateral lemniscus, npo: nuclei of the pons, nto: nucleus of trochlear nerves, rcp: rostral cerebellar peduncle, rf: reticular formation, rst: rubrospinal tract, tmnt: mesencephalic tract of the trigeminal nerve, VI: abducence nerve.
(TIF)

S11 Fig Transverse histological image of the equine brain on the level of the rostral cerebellar peduncle. cst: corticospinal tract, fld: dorsal longitudinal fasciculus, flm: medial longitudinal fasciculus, IaI: lateral lemniscus, mcp: medial cerebellar peduncle, ml: medial lemniscus, nII: nucleus of lateral lemniscus, npo: nuclei of the pons, ppd: parapeduncular nuclei, rcp: rostral cerebellar peduncle, rf: reticular formation, vst: vestibulospinal tract, V: trigeminal nerve.
(TIF)

S12 Fig Transverse histological image of the equine brain on the level of the genu of the facial nerve. dctb: decussation of the trapezoid body, flm: medial longitudinal fasciculus, gnf: genu of the facial nerve, nab: nucleus of the abducent nerve, ncd: dorsal cochlear nucleus, ncv: ventral cochlear nucleus, ndct: superior olivary nucleus, ntsn: nucleus of the spinal tract of the trigeminal nerve, nvl: lateral vestibular nuclei, pyr: pyramidal tract, rcp: rostral cerebellar peduncle, rf: reticular formation, rnf: radix of the facial nerve, rst: rubro-spinal tract, slm: sulcus limitans, tb: trapezoid body, tsnt: spinal tract of the trigeminal nerve, VI: roots of the abducence nerve, VII: facial nerve, VIII: vestibulocochleal nerve.
(TIF)

S13 Fig Transverse histological image of the equine brain on the level of the acoustic tubercle. ccp: caudal cerebellar peduncle, dctb: decussation of the fibres of trapezoid body, flm: medial longitudinal fasciculus, li: lingula of the vermis, mcp: medial cerebellar peduncle, ncd: dorsal cochlear nucleus, ncv: ventral cochlear nucleus, ndct: superior olivary nucleus, ntsn: nucleus of the spinal tract of the trigeminal nerve, nvl: lateral vestibular nuclei, nvm: medial vestibular nucleus, pyr: pyramidal tract, rst: rubrospinal tract, slm: sulcus limitans, tac: acoustic tubercle, tb: trapezoid body, tsnt: spinal tract of the trigeminal nerve, VI: roots of the abducence nerve, VIII: vestibulocochleal nerve.
(TIF)

S14 Fig Transverse histological image of the equine brain on the level of the cuneate nuclei. amb: ambiguus nucleus, ccp: caudal cerebellar peduncle, cun: cunetae nucleus, flm: medial longitudinal fasciculus, hypn: nucleus of the hypoglossal nerve, nfl: nucleus of the lateral fascicle, ntsn: nucleus of the spinal tract of the trigeminal nerve, oli: olivary nucleus, pyr: pyramidal tract, sol: nucleus of the solitary tract, soln: nucleus of the solitary trasct, tsnt: spinal tract of the trigeminal nerve, vagn: nucleus of the vagus nerve, X: vagus nerve.
(TIF)

S15 Fig Transverse histological image of the equine brain on the level of the obex. cec: central canal, cst: corticospinal tract, cun: cuneate nucleus, gra: gracile nucleus, hypn: nucleus of the hypoglossal nerve, nfl: nucleus of the lateral fascicle, ntsn: nucleus of the spinal tract of the trigeminal nerve, obx: obex, oli: olivary nucleus, pyr: pyramidal tract, soln: nucleus of the solitary tract, sol: solitary tract, tsnt: spinal tract of the trigeminal nerve, vgn: nucleus of the vagus nerve.
(TIF)
