# Supplementary material for: Neuroanatomy of the equine brain as revealed by high-field (3Tesla) magnetic-resonance-imaging
Source: PLoS One. 2019 Apr 1;14(4):e0213814. doi: 10.1371/journal.pone.0213814 (PMC6443180; doi:10.1371/journal.pone.0213814)
Supplement: S1 Table — (DOCX) [file pone.0213814.s001.docx]

**Abbreviations list**

**A**

ab: amygdaloid body, Corpus amygdaloideum: 7, 7S, 19-21, 32-34

acn: accumbens nucleus, Nucleus accumbens: 3, 3S, 4, 4S, 19, 20, 28, 29

ah: adenohypophysis, Adenohypophysis: 8, 17, 26, 28, 29

alv: alveus, Alveus: 8, 8S, 21, 23.

amb: ambiguus nucleus, Nucleus ambiguous: 15S

Ans: ansate sulcus, Sulcus ansatus: 1A, 1C-E, 4-6, 6S, 7, 7S, 26, 28, 29.

ans: ansiform lobule, Lobulus ansiformis: 1A, 1C, 13-15, 22, 23, 24, 29, 30-34.

ansl: lenticular ansa, Ansa lenticularis: 6A, 6S.

aq: mesencephalic aqueduct, Aqueductus mesencephalicus: 10, 10S, 11, 11S, 26.

are: entorhinal aera, Area entorhinalis: 8S, 9, 9S, 32.

**B**

bcc: Brachium of the caudal colliculus, Brachium colliculi caudalis: 10, 10S, 11, 11S, 21, 30, 31.

bcr: Brachium of the rostral colliculus, Srachium colliculi rostralis: 28, 29.

boc: Basioccipital bone, Os basilare: 26.

bsp: Basisphenoid bone, Os basisphenoidale: 26.

**C**

CA1: Cornu ammonis Field 1: 9S.

CA2: Cornu ammonis Field 2: 9S.

CA3: Cornu ammonis Field 3: 9S.

CA4: Cornu ammonis Field 4: 9S.

cam: ammon’s horn, Cornu ammonis: 8, 9, 9S, 20, 22, 23, 26-33.

cc: Corpus callosum: 1E, 3S, 5, 5S, 6, 6S, 7, 7S, 8, 8S, 23, 26-29.

ccc: commissure of the caudal colliculus, Commissura colliculi caudalis: 11, 26, 27.

ccd: caudal commissure, Commissura caudalis: 9, 9S, 26, 27.

ccp: caudal cerebellar peduncle, Pedunculus cerebellaris caudalis: 13, 14, 14S, 15S, 29, 30.

ccr: commissure of the rostral colliculus, Commissura colliculi rostralis: 10.

cdc: caudal colliculus, Colliculus caudalis: 9, 9S, 11, 11S, 28.

cec: central canal, Canalis centralis: 16S, 27.

cf: column of fornix, Columna fornicis: 7, 7S, 19, 27, 28.

cfo: corpus of fornix, Corpus fornicis: 6, 7, 7S, 21, 22.

cgs: central grey substance, Substantia grisea centralis: 9, 9S, 10, 10S, 11, 19, 27.

cha: habenular commissure, Commissura habenularum: 9, 9S, 22.

cho: optic chiasm, Chiasma opticum: 1S, 5, 5S, 6, 6S, 17, 26, 27.

chp: choroid plexus, Plexus choroideus: 1S, 5, 6, 20, 21, 26, 27, 29-32.

cig: cingulated gyrus: 2, 3, 3S, 5, 5S, 6, 6S, 8, 9, 24-26, 28-30.

cin: cingulum: 2, 3, 3S, 5, 5S, 6, 6S, 8, 9, 24-26, 28, 29, 30

Cing: Cingulate sulcus: Fig 1 C, 2

cl: central lobule, Lobulus centralis: 11, 12, 21, 22, 23, 26, 27, 28.

cla: claustrum, Claustrum: 3, 3S, 4, 4S, 5, 5S, 6, 6S, 8S, 22, 33, 34.

cmf: commissure of fornix, Commissura fornicis: 8, 28, 29.

cn: caudate nucleus, Nucleus caudatus: 3, 3S, 4, 4S, 5, 5S, 6, 6S, 7, 7S, 8, 8S, 9S, 21, 22, 23, 27-31.

cor: coronal gyrus, Gyrus coronalis: 2S, 3, 3S.

Cor: coronal sulcus, Sulcus coronalis: 1A, 1C, 1D, 1E, 2, 2S, 3, 3S, 4, 6, 23, 24, 25, 30, 31.

crc: cerebral crus, Crus cerebri: 1S, 7, 7S, 8, 8S, 9, 9S, 10, 18, 19, 20, 22, 29-31

crt: rubro-cerebello-thalamic tract, Tractus cerebello-rubro-thalamicus: 8, 9, 11, 19, 21, 26, 28.

Cru: cruciate sulcus, Sulcus cruciatus: 1C, 1D, 1E, 2, 2S, 3S, 24, 25, 28, 29.

cso: supraoptic commissure, Commissura supraoptica: 3, 3S, 4, 4S, 5, 5S, 6, 6S.

cst: corticospinal tract, Tractus corticospinalis: 11S, 12, 12S, 16S, 17, 29.

ctt: central tegmental tract, Tractus tegmentalis centralis: 10, 10S.

cu: culmen: 11, 12, 13, 22, 23, 24, 26, 27, 28.

cun: cuneate nucleus, Nucleus cuneatus: 15, 15S, 16, 16S.

**D**

dbb: diagonalband of broca, Stria diagonalis: 1S, 1E, 5, 5S, 19.

dcml: decussation of medial lemniscus, Decussatio lemniscorum medialis: 17.

dctb: decussation of the fibres of trapezoid body, Decussatio trapezoideum: 14, 14S, 17.

de: declive of the vermis, Declive vermis: 14, 24, 26, 28.

dg: dentate gyrus, Gyrus dentatus: 8, 8S, 9, 9S, 21.

Dia: diagonal sulcus, Sulcus diagonalis: 1A, 1D, 2, 2S, 3, 3S, 4, 5, 5S, 21-24, 31-34.

dpy: pyramidal decussation, Decussatio pyramidum: 28.

drp: decussation of the rostral cerebellar peduncle, Decussatio pedunculorum cerebellarium rostralium: 10, 20, 26-28.

dtd: dorsal tegmental decussation, Decussatio tegmentalis dorsalis: 27.

dtn: decussation of trochlear nerve, Decussatio nervorum trochlearium: 11S, 21, 26, 27.

**E**

ec: external capsule, Capsula externa: 3S, 4, 4S, 5, 5S, 6, 6S, 7, 7S, 22, 32.

ecs: ectosylvian gyrus, Gyrus ectosylvius: 1A, 4, 5, 9, 34.

Ecs: ectosylvian sulcus, Sulcus ectosylvius: 1A, 1C, 2S, 3, 3S, 4-7, 7S, 8, 9, 10, 12, 22-25, 34.

ectg: ectogenual gyrus, Gyrus ectogenualis: 28.

Ectg: ectogenual sulcus, Sulcus ectogenualis: 2, 2S, 26.

ectm: ectomarginal gyrus, Gyrus ectomarginalis: 25, 29-31.

Ectm: ectomarginal sulcus, Sulcus ectomarginalis: 1A, 1C, 8, 9, 10, 11, 12, 13, 23-25, 33.

Eng: endogenual sulcus, Sulcus endogenualis: 1E, 3, 4S, 23, 26, 27.

enm: endomarginal gyrus, Gyrus endomarginalis: 25, 28.

Enm: endomarginal sulcus, Sulcus endomarginalis: 1C, 1E, 8, 10-12, 23-26, 28.

enrh: endorhinal sulcus, Sulcus endorhinalis: 5, 5S.

Ensp: endosplenial sulcus, Sulcus endosplenialis: 1E.

Espl: ectosplenial sulcus, Sulcus ectosplenialis: 11, 26.

ex: extreme capsule, Capsula extrema: 3, 4, 4S, 5, 5S, 6, 6S, 7, 7S, 33, 34.

**F**

fh: fimbria of the hippocampus, Fimbria hippocampi: 8, 8S, 9S, 20, 30.

Flc: longitudinal cerebral fissure, Fissura longitudinalis cerebri: 24, 25.

fld: dorsal longitudinal fasciculus, Fasciculus longitudinalis dorsalis: 12S.

flm: medial longitudinal fasciculus, Fasciculus longitudinalis medialis: 10, 11, 11S, 12, 12S, 13, 13S, 14, 14S, 15, 15S, 19, 20, 26-28.

flo: flocculus, Flocculus: 1, 13, 14, 30-32.

flv: ventral longitudinal fasciculus, Fasciculus longitudinalis ventralis: 9, 9S

fmt: mammilo-thalamical fasciculus, Fasciculus mammillo-thalamicus: 7, 7S, 18, 19, 20, 27-29.

fo: fornix, Fornix: 6, 18, 20, 23, 26, 27.

fol: folium of the vermis, Folium vermis: 26.

Fp: primary fissure, Fissura prima: 23, 26-29.

fpaf: paraflocculare fissure, Fissura parafloccularis: 23.

Fpc: praeculminate fissure, Fissura praeculminata: 23, 26, 27.

fsc: subcallosal fasciculus, Fasciculus subcallosus: 3, 3S, 4, 4S, 5, 5S, 6, 6S, 7, 7S, 8, 8S, 9, 9S, 23, 30.

ftp: transversal fibres of the pons, Fibrae transversae pontis: 11, 11S, 27, 29, 30.

**G**

gcc: genu of the corpus callosum, Genu corporis callosi: 3, 3S, 4, 4S, 22, 26, 28.

gen: genual gyrus, Gyrus genualis: 28.

Gen: genual sulcus, Sulcus genualis: 1E, 2, 2S, 3, 3S, 4, 4S, 21, 23, 26, 27.

gg: Gasserian ganglion, Ganglion trigeminale (Gasser): 12, 18, 19, 20, 32-34

gnf: genu of the facial nerve, Genu nervi facialis: 13, 13S, 20, 28.

gp: Globus pallidus: 5, 5S, 6, 6S, 21, 30, 31.

gra: gracile nucleus, Nucleus gracilis: 16S

grcl: granula cell layer, Stratum granulare: 8C

**H**

H1: fields of Forel 1, Fasciculus thalamicus: 8S.

H2: fields of Forel 2, Fasciculus lenticularis: 8S.

ha: habenula, Habenula: 8, 8S.

han: habenular nuclei, Nuclei habenulares: 8, 8S, 22.

hf: hippocampal fissure, Fissura hippocampi: 8, 8S.

hip: hippocampus proper, hippocampus proper: 21.

hit: habenulo-interpeduncular tract, Tractus habenulo-interpeduncularis: 8, 8S, 21, 22, 27, 28, 30, 31.

hs: hypothalamic sulcus: 7, 7S.

hyp: hypothalamus, Hypothalamus: 18, 19.

hypn: nucleus of the hypoglossal nerve, Nucleus nervi hypoglossi: 15, 15S, 16, 16S.

**I**

ic: internal capsule, Capsula interna: 3S, 4, 4S, 5, 5S, 6, 6S, 7, 7S, 19, 21, 22, 23, 30, 32-34.

icl: Island of Callela, insula callelae: 5

icrs: intercrural sulcus, Sulcus intercruralis: 15.

idg: supracallosal gyrus, Indusium griseum: 8.

inf: infundibular stalk, Pars infundibularis: 7, 17, 26.

ins: insular cortex, Cortex insularis: 5, 8S.

ipd: interpeduncular nucleus, Nucleus interpeduncularis: 10, 11, 11S, 26.

ir: infundibular recess, Recessus infundibularis: 17.

ita: interthalamic adhesion, Adhaesio interthalamica: 7, 7S.

**L**

lal: lateral lemniscus, Lemniscus lateralis: 11, 11S, 12S, 19, 29.

lgb: lateral geniculate body, Corpus geniculatum laterale: 8, 8S, 9, 9S, 22, 29-32.

li: lingula of the vermis, Lingula vermis: 13, 14, 14S, 20, 21, 26, 27.

lme: external medullary lamina, Lamina medullaris externa: 7, 7S, 8S, 21, 22.

lmi: internal medullary lamina, Lamina medullaris interna: 29, 30.

Lms: lateral mesencephalic sulcus, Sulcus mesencephalicus lateralis: 10.

log: lateral olfactory gyrus, Gyrus olfactorius latralis: 1S, 3, 3S, 5, 5S.

lot: lateral olfactory tract, Tractus olfactorius lateralis: 2, 2S, 3, 3S, 4, 4S, 5, 5S, 6, 6S, 18, 19, 30.

lt: terminal lamina, Lamina terminalis: 26, 6S

lv: lateral ventricle, Ventriculus lateralis: 3S, 5, 5S, 6S, 7, 7S, 8, 8S, 23, 30.

**M**

man: mandibular nerve, Nervus mandibularis: 31, 34.

mar: marginal gyrus, Gyrus marginalis: 5, 9, 11, 25, 28, 29.

Mar: marginal sulcus, Sulcus marginalis: 1C, 5, 7, 7S, 8, 9, 10, 12, 13, 23-25.

max: maxillary nerve, Nervus maxillaris: 6, 7, 8, 17, 32, 34.

mb: mamillary body, Corpus mammillare: 18, 26, 27.

mcp: medial cereellar peduncle, Pedunculus cerebellaris medialis: 11, 11S, 12, 12S, 14S, 18-21.

mgb: medial geniculate body, Corpus geniculatum mediale: 8, 8S, 9, 9S, 11, 21, 29, 31, 32.

ml: medial lemniscus, Lemniscus medialis: 8, 8S, 9, 9S, 10, 11A, 11S, 12, 12S, 17, 18, 19, 20, 27, 28.

mot: medial olfactory tract, Tractus olfactorius medialis: 2, 2S, 3, 3S, 4S.

mtn: motor nucleus of the trigeminal nerve, Nucleus motorius nervi trigemini: 29.

**N**

nad: nucleus anterior dorsalis thalami: 7, 7S.

nab: nucleus of the abbducent nerve, Nucleus nervi abducentis: 13, 13S, 20.

nca: nucleus of the caudal colliculus: 11

ncd: dorsal cochlear nucleus, Nucleus cochlearis dorsalis: 13, 13S, 14, 14S.

ncv: ventral cochlear nucleus, Nucleus cochlearis ventralis: 13, 13S, 14S.

nd: dentate nucleus, Nucleus dentatus: 22, 29-32.

ndct: superior olivary nucleus, Nucleus dorsalis corpus trapezoidei: 14, 14S

nf: fastigial nucleus, Nucleus fastigii: 14, 22, 26, 27.

nfl: nucleus of the lateral fascicle, Nucleus fasciculi lateralis: 15, 15S, 16, 16S.

nh: neurohypophysis, Neurohypophysis: 17, 26.

nip: interpositus nucleus, Nucleus interpositus: 14, 22, 28, 29.

nll: nucleus of lateral lemniscus, nucleus lemnisci lateralis: 11S, 12S.

nmt: mesencephalic tract of the trigeminal nerve, Tractus mesencephalicus nervi trigemini: 10S.

nnf: nucleus of the facial nerve, Nucleus nervi facialis: 13, 13S, 18.

no: nodulus of the vermis, Nodulus vermis: 26-29.

nom: nucleus of the oculomotor nerve, Nucleus nervi oculomotorii: 10.

npo: nuclei of the pons, Nuclei pontis: 11, 11S, 12, 12S, 28, 29.

nrt: reticular nucleus of the thalamus, Nucleus reticularis thalami: 7, 7S, 8S, 21, 22, 30, 31.

nto: nucleus of trochlear nerve, Nucleus nervus trochlearis: 11, 11S.

ntsn: nucleus of the spinal tract of the trigeminal nerve, Nucleus tractus spinalis nervi trigemini: 13S, 14, 14S, 15, 15S, 16, 16S, 18, 19, 30.

nvg: nucleus of the vagal nerve, Nucleus nervi vagi: 28.

nvl: lateral vestibular nuclei, Nuclei vestibulares laterales: 13, 13S, 14, 14S.

nvm: medial vestibular nucleus, nucleus vestibularis medialis: 13, 14, 14S, 20, 28.

**O**

ob: olfactory bulb, Bulbus olfactorius: 1ACD, 19 20, 29-32.

obl: oblique gyrus, Gyrus obliquus: 1, 7, 9.

Obl: oblique sulcus, Sulcus obliquus: 1, 1C, 24.

obx: obex, Obex: 16S, 26.

olf: olfactory fibres, Filae olfactoriae: 20, 27-32.

oli: olivary nucleus, Nucleus olivaris: 15, 15S, 16, 16S, 17, 27-30.

olr: olfactory recess, Recessus olfactorius: 2, 2S, 18, 20.

Ols: olfactory sulcus, Sulcus olfactorius: 2, 2S.

omn: oculomotor nerve, Nervus oculomotorius: 27.

op: olfactory peduncle, Pedunculus olfactorius: 1S, 18, 29.

opn: optic nerve, Nervus opticus: 1S, 3, 4, 27, 28, 30.

opth: ophthalmic nerve, Nervus ophthalmicus: 32.

or: optic radiation, Radiatio optica: 8S, 9, 9S, 22, 24, 31-34.

ot: optic tract, Tractus opticus: 7, 8, 8S, 9, 9S, 18, 19, 20, 21, 22, 29, 30.

otb: olfactory tubercle, Tuberculum olfactorium: 1S, 4, 5S, 18, 19, 29, 30.

**P**

paf: paraflocculus, Paraflocculus: 1 A,13, 14, 22, 23, 24, 32, 33.

pb: pineal body, Corpus pineale: 9, 10, 10S, 26.

pcm: peduncles of the mammillary Sody, Pedunculi corporis mammilares: 8, 8S, 9, 9S, 17.

pdt: thalamic peduncle, Pedunculus thalami: 29.

prpc: prepiriform cortex: 4, 4S, 6, 6S

pfc: piriform cortex: 7S.

pfs: perforated substance, Substantia perforata: 19.

pg: pituitary gland, Glandula pituitaria: 1S, 7, 8, 9.

phg: parahippocampal gyrus, Gyrus parahippocampalis: 8, 9, 20, 21, 30, 33, 34

pir: piriform lobe, Lobus piriformis: 7A, 7S, 19, 31.

pml: paramedian lobule, Lobulus paramedianus: 1C, 14, 15, 22, 23, 29, 31.

po: pons, Pons: 1A, 1S, 17, 26, 27, 31.

poa: preoptic area, Area praeoptica: 19, 28, 29.

ppd: parapeduncular nuclei, Nuclei parapedunculares: 12, 12S, 29.

prpc: praepiriform cortex, Cortex praepiriformis: 21.

prr: prorean gyrus, Gyrus proreus: 1, 2, 2S, 19, 26, 30.

Prr: prorean sulcus, Sulcus proreus: 1A, 2, 2S, 19, 20, 22, 28, 31, 32.

Prs: presylvian sulcus, Sulcus praesylvius: 1A, 1C, 1D, 2, 2S, 3, 3S, 21, 22, 31-34.

psi: pars intermedia of the pituitary gland, Pars intermedia glandulae pituitariae: 26, 28.

pta: pretectal area, Area praetectalis: 9S, 22, 28, 29.

pul: pulvinar, Pulvinar: 8, 8S, 9, 9S, 22.

put: putamen, Putamen: 3, 3S, 4, 4S, 5, 5S, 6, 6S, 7, 7S, 20, 21, 22, 32.

py: pyramis of the vermis, Pyramis vermis: 14, 15, 16, 21, 26-28.

pyr: pyramidal tract, Tractus pyramidalis: 1S, 13, 13S, 14S, 15, 15S, 16, 6S, 26, 28, 29.

**Q**

qdr: quadrangular lobule, Lobulus quadrangularis: 12, 23, 29, 30-33.

**R**

rc: rostral commissure, Commissura rostralis: 5, 5S, 6, 6S, 20, 26-29

rcc: radiation of corpus callosum, Radiatio corporis callosi: 3, 4, 4S, 5, 5S, 6, 6S, 7, 7S, 8, 9, 21, 26-28, 30.

rccl: rostrum of the corpus callosum, Rostrum corporis callosi: 26-28.

rcp: rostral cerebellar peduncle, Pedunculus cerebellaris rostralis: 11, 11S, 12, 12S, 13S, 14, 20, 21, 28-32.

rf: reticular formation, Formatio reticularis: 11, 11S, 12, 12S, 13S, 18, 19, 28.

Rfi: rhinal fissure, Fissura rhinalis: 1A, 2, 2S, 3, 3S, 4, 4S, 5, 5S, 6, 6S, 7, 7S, 8, 8S, 9, 9S, 10, 11, 12, 18, 20, 21, 27, 30-34.

rmt: motor root of the trigeminal nerve, Radix motoria nervi trigemini: 12.

rn: red nucleus, Nucleus ruber: 9, 9S, 27.

rnf: root of the facial nerve, Radix nervi facialis: 13S.

rpb: recess of the pineal body: 26

roc: rostral colliculus, Colliculus rostralis: 10, 22, 26-28.

rsn: sensory root of the trigeminal nerve, Radix sensoria nervi trigemini: 12, 12S, 20.

rst: rubrospinal tract, Tractus rubrospinalis: 11S, 13S, 12-14, 14S, 18, 21, 29.

**S**

scc: splenium of corpus callosum, Splenium corporis callosi: 9, 9S, 26-28.

Scf: secondary fissure, Fissura secunda: 26, 27.

scg: subcallosal gyrus, Gyrus subcallosus: 19, 26.

Scl: sulcus of corpus callosum, Sulcus corporis callosi: 3S, 5, 5S, 6S, 26.

scmo: subcommissural organ, Organum subcommissurale: 9S.

scn: supracommissurale nucleus, Nucleus supracommissuralis: 18.

Sgs: sagittal sulcus, Sulcus sagittalis: 1A, 1S, 8, 8S, 8C.

sl: lateral septal nuclei, Nuclei septales laterales: 4, 4S, 5, 5S, 21, 26.

slm: sulcus limitans, Sulcus limitans: 10S, 13S, 14, 14S.

slu: semilunar gyrus, Gyrus semilunaris: 1S.

sm: medial septal nuclei, Nuclei septales mediales: 4S, 5, 5S, 27.

smt: medullary stria of the thalamus, Stria medullaris thalami: 6, 6S, 7, 7S, 21, 22, 26-29.

sn: septal nuclei, Nuclei septales: 26.

snr: substantia nigra, Substantia nigra: 8, 8S, 9, 9S, 10, 19, 28-31.

snrc: pars compacta of the substantia nigra: S9

snrr: pars reticularis of the substantia nigra: S9

soln: nucleus of the solitary tract, Nucleus tractus solitarii: 15S, 16, 16S.

sol: solitary tract, Tractus solitarius: 16S.

Spl: splenial sulcus, Sulcus splenialis: 1E, 5, 5S, 6, 6S, 8, 9, 10, 11, 23-27.

Spm: paramedian sulcus, Sulcus paramedianus: 23.

ssg: suprasylvian gyrus, Gyrus suprasylvius: 3S, 4, 5, 7, 7S, 9, 31-33.

Sspl: suprasplenial sulcus, Sulcus suprasplenialis: 1E, 9, 12, 24, 25, 26.

Sss: suprasylvian sulcus, Sulcus suprasylvius: 1A, 1C, 2, 2S, 3, 3S, 4, 5, 6, 7, 7S, 8-13, 23-25, 32, 33.

stn: subthalamic nucleus, Nucleus subthalamicus: 29.

sto: stratum opticum of the rostral colliculus, Stratum opticum colliculi rostralis: 28.

stse: external stratum sagittale, Stratum sagittale externum: 11.

stsi: internal stratum sagittale, Stratum sagittale internum: 11.

stt: terminal stria, Stria terminalis: 6S, 7S, 8S, 29.

sub: subiculum, Subiculum: 9S, 21.

Syl: sylvian fissure, Fissura sylvii: 1A, 1C, 6, 6S, 7, 7S, 8, 8S, 22, 23, 34.

syl: sylvian gyrus, Gyrus sylvius: 1A, 4, 7, 7S.

**T**

tb: trapezoid body, Corpus trapezoideum: 1B, 13, 13S, 14, 14S, 17, 27, 29.

tac: acoustic tubercle, tuberculum acusticum: 14, 14S

tfp: transvers fibres of pons, Fibrae transversae pontis: 28.

th: thalamus, Thalamus: 22, 26, 28-31.

tmnt: mesencephalic tract of the trigeminal nerve: 11

trcn: trochlear nucleus, Nucleus trochlearis: 21.

tsnt: spinal tract of the trigeminal nerve, Tractus spinalis nervi trigemini: 13, 13S, 14, 14S, 15, 15S, 16, 16S, 18, 19, 30.

tu: tuber of the vermis, Tuber vermis: 15, 21-24, 26.

**U**

uv: uvula of the vermis, Uvula vermis: 15, 16, 26, 27.

**V**

ver: vermis, Vermis: 1C.

vgn: nucleus of the vagus nerve, Nucleus nervi vagi: 15, 15S.

vst: vestibulospinal tract, Tractus vestibulospinalis: 12, 12S.

vtc: ventral tegmental commissure, Commissura tegmentalis ventralis: 9S.

vtd: ventral tegmental decussation, Decussatio tegmentalis ventralis: 9, 19, 27.

**Z**

zi: zona incerta, Zona incerta: 8, 8S, 28, 29.

**Cranial nerves**

II: optic nerve, Nervus opticus: 17, 29, 31.

III: oculomotor nerve, Nervus oculomotorius: 6, 7, 8, 28, 29, 30.

V: trigeminal nerve, Nervus trigeminus: 1,10, 11, 12S, 32.

VI: abducence nerve, Nervus abducens: 11S, 13S, 14S.

Vll: facial nerve, Nervus facialis: 13, 13S, 17, 19, 21, 29, 30, 32.

Vlll: vestiSulocochleal nerve, Nervus vestiSulocochlearis: 13, 13S, 14S, 17, 21, 32.

X: vagus nerve, Nervus vagus: 15, 15S, 16, 18, 20.

XII: hypoglossal nerve, Nervus hypoglossus: 16, 18

Arabic numbers

3: third ventricle, Ventriculus tertius: 19, 22, 26.

4: fourth ventricle, Ventriculus quartus: 26, 27.

Blood vessels

acc: corpus callosum artery, Arteria corporis callosi: 20, 26.

aci: internal carotid artery, Arteria carotis interna: 7.

bsa: basilar artery, Arteria basilaris: 17.

cca: caudal cerebral artery, Arteria cerebri caudalis: 19, 20, 30.

ccba: caudal cerebellar artery, Arteria cerebelli caudalis: 17, 21.

ccma: caudal communicating artery, Arteria communicans caudalis: 17, 26.

cvs: cavernus sinus, Sinus cavernosus: 17, 30.

icvs: intercavernous sinus, Sinus intercavernosus: 17, 26, 28, 30.

mca: medial cerebral artery, Arteria cerebralis medialis: 5, 6, 18, 19, 20, 22.

rca: rostral cerebral artery, Arteria cerebri rostralis: 4, 17, 18, 26.

rcba: rostral cerebellar artery, Arteria cerebellaris rostralis: 18.

rcma: rostral communicating artery, Arteria communicans rostralis: 18, 30.

sre: straight sinus, Sinus rectus: 27.

stra: striate artery, Ramus striatus arteriae cerebri mediae: 6.

trvs: transverse sinus, Sinus transversus: 13.
