# Supplementary figures and images for: Neuroanatomy of the equine brain as revealed by high-field (3Tesla) magnetic-resonance-imaging
Source: PLoS One. 2019 Apr 1;14(4):e0213814. doi: 10.1371/journal.pone.0213814 (PMC6443180; doi:10.1371/journal.pone.0213814)

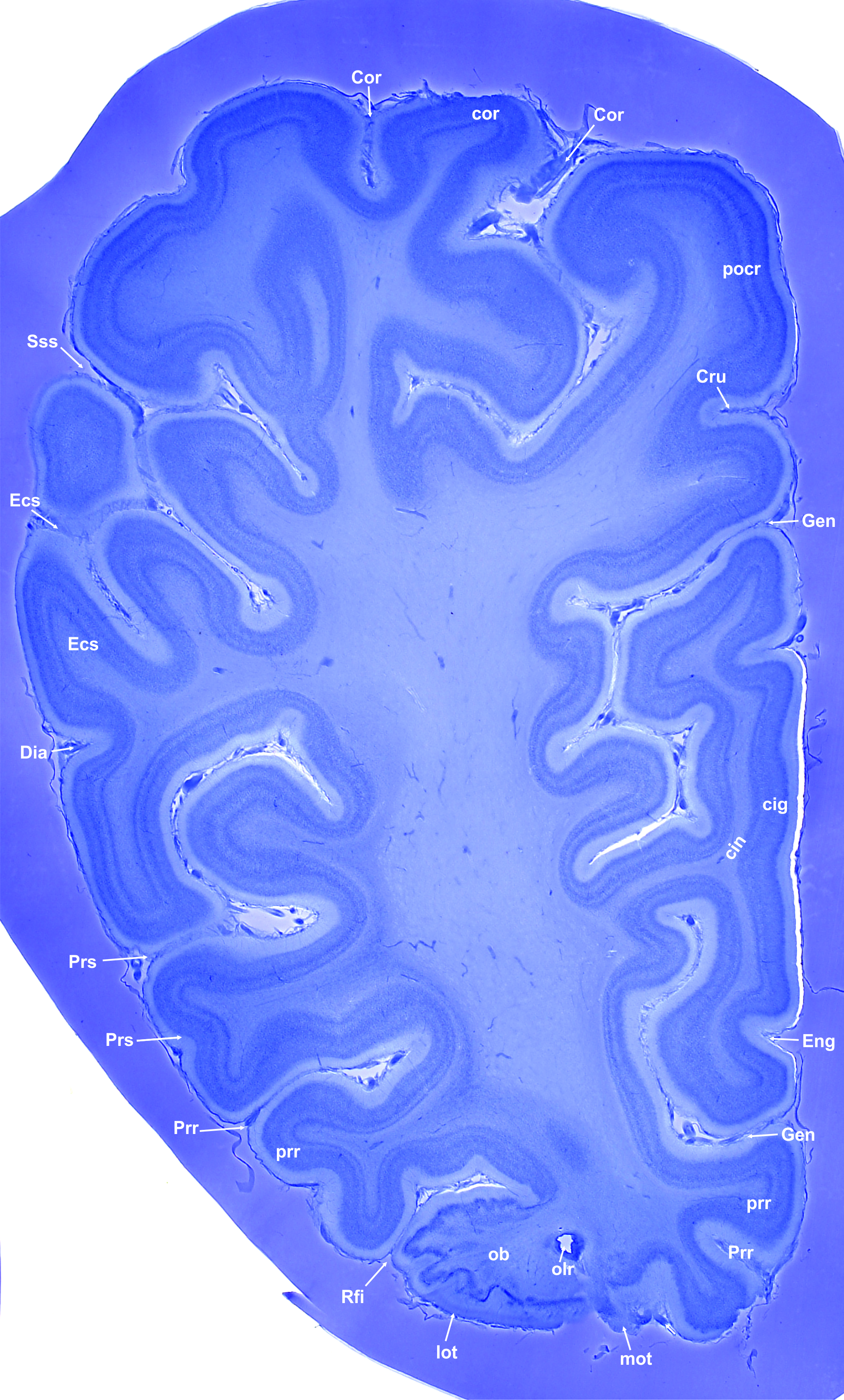

Supplement: S1 Fig — cin: cingulum, cig: cingulate gyrus, Cor: coronal sulcus, cor: coronal gyrus, Cru: cruciate sulcus, Dia: diagonal sulcus, Ectg: ectogenula sulcus, Ecs: ectosylvian sulcus, Gen: genual sulcus, lot: lateral olfactory tract, mot: medial olfactory tract, olr: olfactory recess, Prr: prorean sulcus, prr: prorean gyrus, Prs: presylvian sulcus, Rfi: rhinal fissure, Sss: suprasylvian sulcus. (TIF) [file pone.0213814.s002.tif]

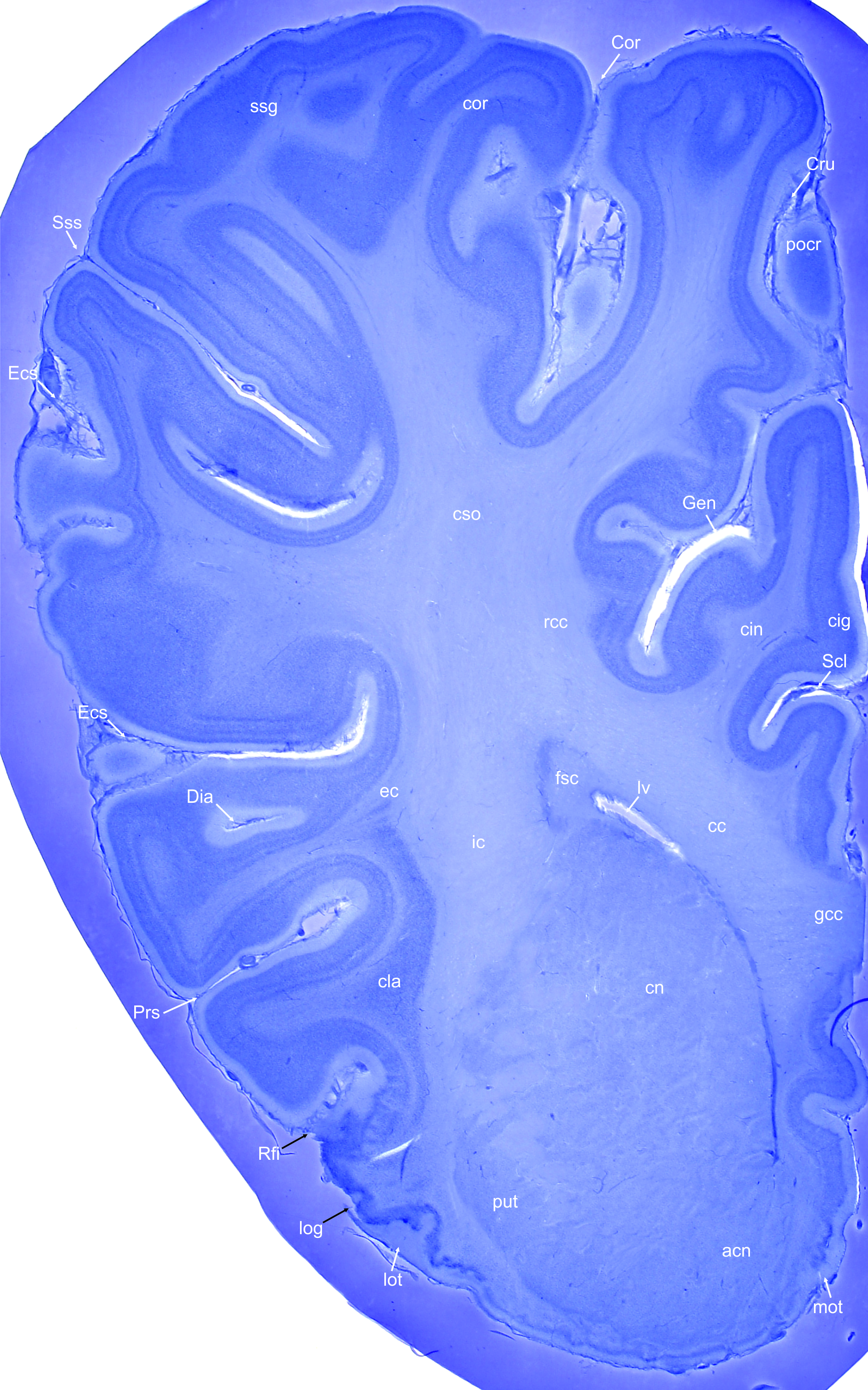

Supplement: S2 Fig — acn: accumbens nucleus, cc: colossal commissure, cin: cingulum, cla: claustrum, cn: caudate nucleus, cor: coronal gyrus, Cor: coronal sulcus, Cru: cruciate sulcus, cso: centrum semiovale, Dia: diagonal sulcus, ec: external capsule, Ecs: ectosylvian sulcus, fsc: subcallosal fasciculus, gcc: genu of the corpus callosum, Gen: genual sulcus, ic: internal capsule, log: lateral olfactory gyrus, lot: lateral olfactory tract, lv: lateral ventrikel, mot: medial olfactory tract, Prs: presylvian sulcus, put: putamen, Rfi: rhinal fissure, Scl: sulcus of corpus callosum, ssg: suprasylvian gyrus, Sss: suprasylvian sulcus. (TIF) [file pone.0213814.s003.tif]

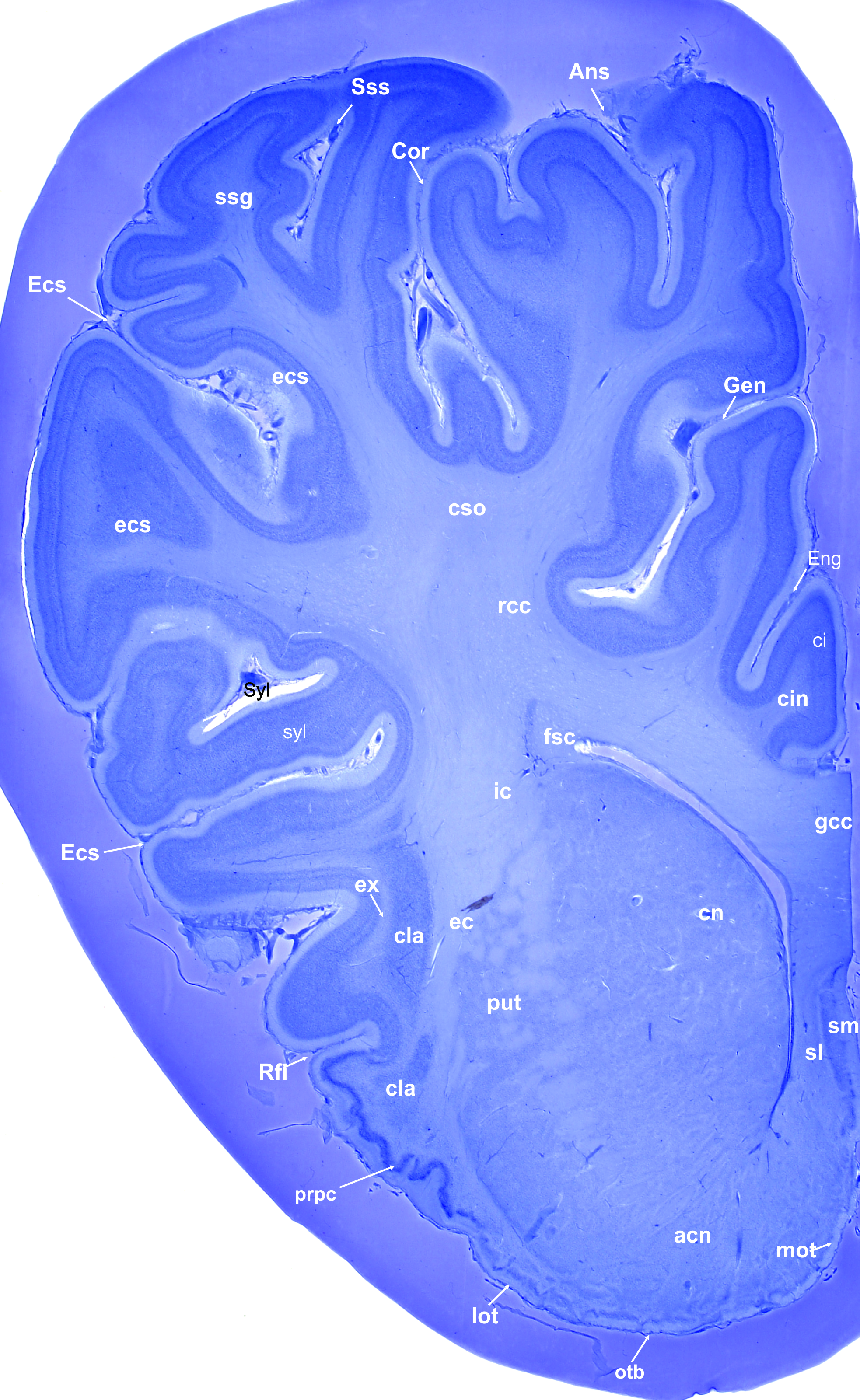

Supplement: S3 Fig — acn: accumbens nucleus, Ans: Ansate sulcus, cla: claustrum, cig: cingulate gyrus, cin: cingulum, cn: caudate nucleus, Cor: coronal sulcus, cso: centrum semiovale, ec: external capsule, Ecs: ectosylvian sulcus, Eng: endogenual sulcus, ex: extreme capsule, fsc: subcallosal fasciculus, gcc: genu of the corpus callosum, Gen: genual sulcus, ic: internal capsule, lot: lateral olfactory tract, mot: medial olfactory tract, otb: olfactory tubercle, put: putamen, rcc: radition of corpus callosum, Rfi: rhinal fissure, sl: lateral septal nuclei, sm: medial septal nuclei. (TIF) [file pone.0213814.s004.tif]

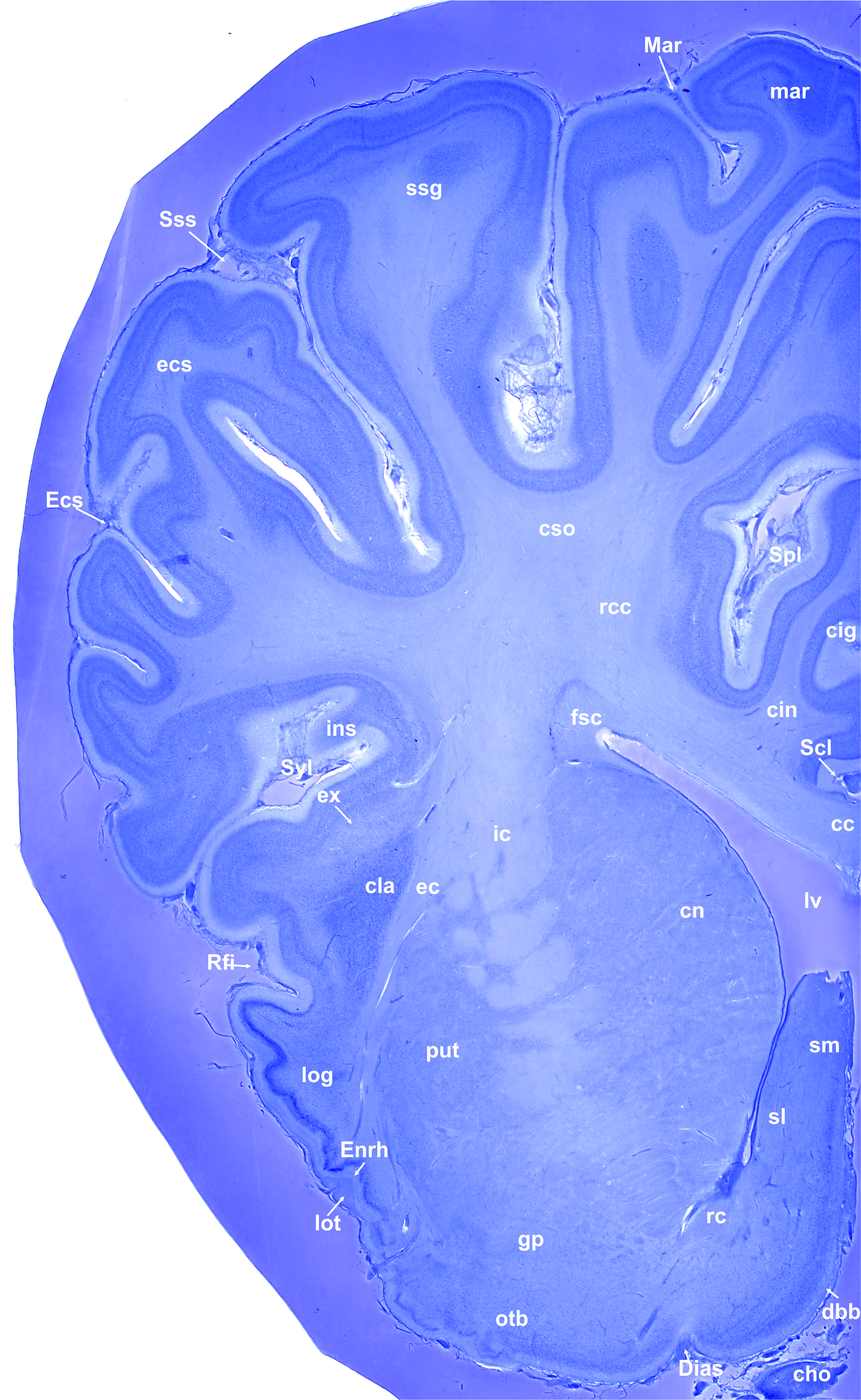

Supplement: S4 Fig — cc: callosal commissure, cho: optic chiasm, cig: cingulate gyrus, cin: cingulum, cla: claustrum, cn: caudate nucleus, cso: centrum semiovale, dbb: diagonal band of broca, Dias: diagonal sulcus (Rhinencephalon), ec: external capsule, ecs: ectosylvian gyrus, Ecs: ectosylvian sulcus, Enrh: endorhinal sulcus, ex: extreme capsule, fsc: subcallosal fasciculus, gp: globus pallidus, ic: internal capsule, icl: islands of Calleja, ins: insular cortex, log: lateral olfactory gyrus, lot: lateral olfactory tract, lv: lateral ventricle, otb: olfactory tubercle, put: putamen, rc: rostral commissure, rcc: radiation of corpus callosum, Rfi: rhinal fissure, Scl: sulcus of corpus callosum, sl: lateral septal nuclei, sm: medial septal nuclei, Spl: splenial sulcus, Sss: suprasylvian sulcus, Syl: Sylvian fissure. (TIF) [file pone.0213814.s005.tif]

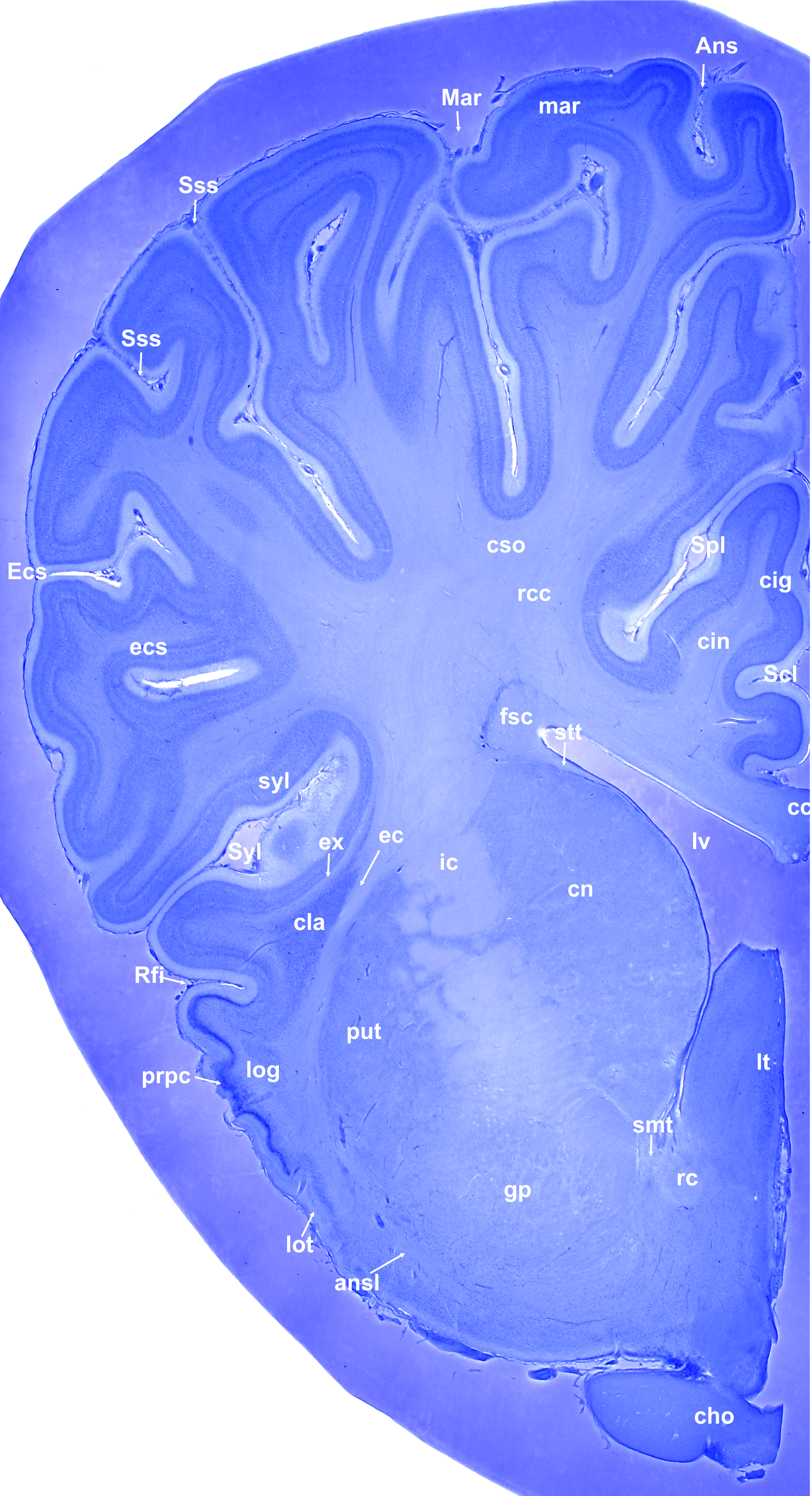

Supplement: S5 Fig — Ans: ansate sulcus, ansl: ansa lenticularis, cc: corpus callosum, cho: optic chiasm, cig: cingulate gyrus, cin: cingulum, cla: claustrum, cn: caudate nucleus, cso: supraoptic commissure, ec: external capsule, Ecs: ectosylvian sulcus, ectosylvian gyrus, ex: extreme capsule, fsc: subcallosal fasciculus, gp: globus pallidus, ic: internal capsule, log: lateral olfactory gyrus, lot: lateral olfactory tract, lv: lateral ventricle, Mar: marginal sulcus, mar: marginal gyrus, prpc: prepiriform cortex, put: putamen, rc: rostral commissure, rcc: radiation of corpus callosum, Rfi: rhinal fissure, Scl: sulcus of corpus callosum, smt: stria medullaris thalami, Spl: splenial sulcus, Sss: suprasylvian sulcus, stt: terminal stria, Syl: sylvian fissure, syl: sylvian sulcus, tl: terminal lamina. (TIF) [file pone.0213814.s006.tif]

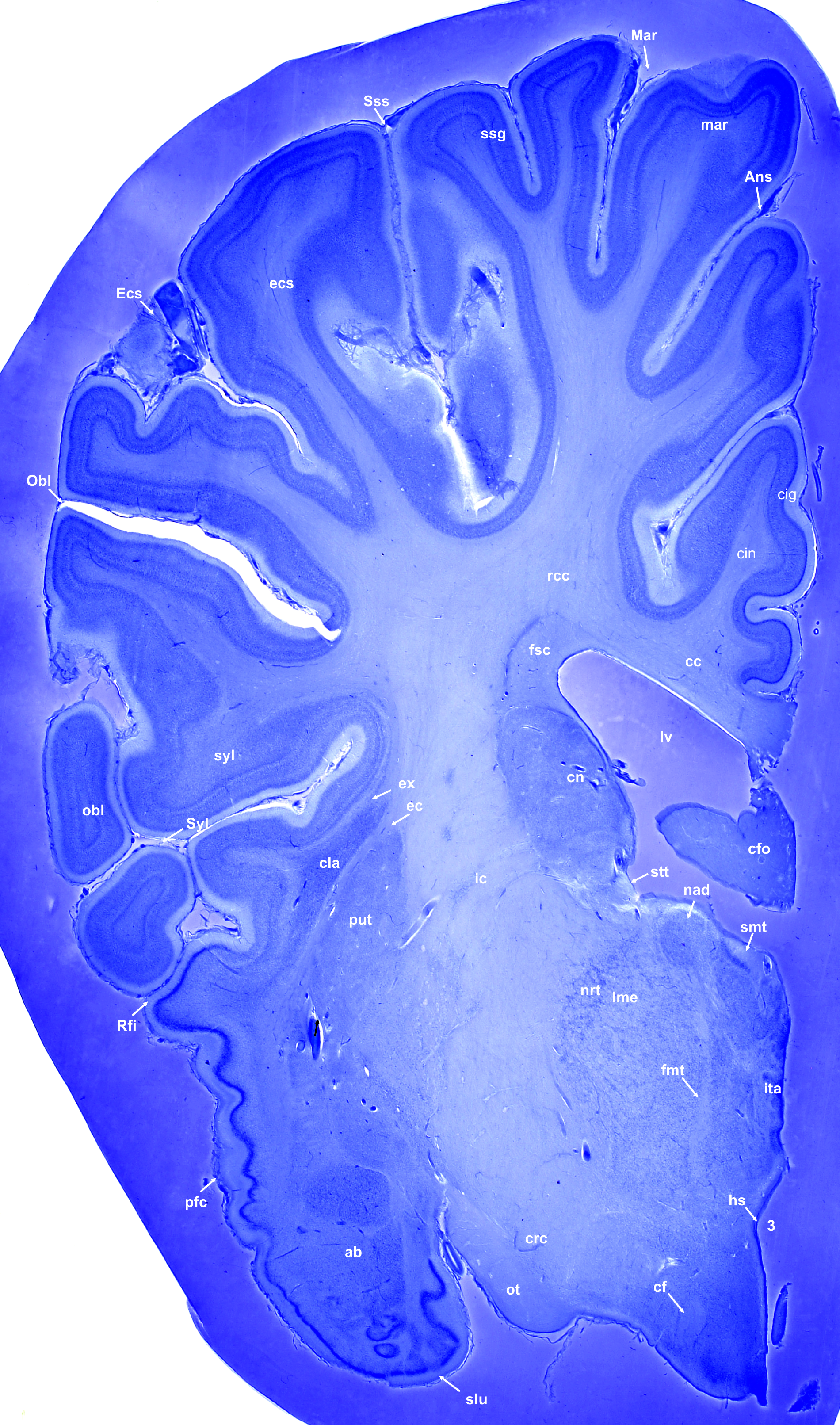

Supplement: S6 Fig — ab: amygdaloid body,alv: alveus, Ans: ansate sulcus, cc: corpus callosum, cin: cingulum, cig: cingulate gyrus, cf: column of fornix, cfo: corpus of fornix, cn: caudate nucleus, crc: cerebral crus, ec: external capsule, Ecs: ectosylvian sulcus, ecs: ectosylvian gyrus, ex: extreme capsule, fmt: mammilo-thalamic fasciculus, fsc: subcallosal fasciculus, hs: hypothalamic sulcus, ic: internal capsule, ita: interthalamic adhesion, lme: external medullary lamina, lv: lateral ventricle, Mar: marginal sulcus, mar: marginal gyrus, nad: nucleus anterior dorsalis thalami, nrt: reticular nucleus of the thalamus, Obl: oblique sulcus, obl: oblique gyrus, pfc: piriform cortex, put: putamen, rcc: radiation of corpus callosum, Rfi: rhinal fissure, slu: gyrus semilunaris, smt: stria medullaris thalami, ssg: suprasylvian gyrus, Sss: suprasylvian sulcus, stt: terminal stria, syl: sylvian gyrus, Syl: sylvian fissure, 3: third ventricle. (TIF) [file pone.0213814.s007.tif]

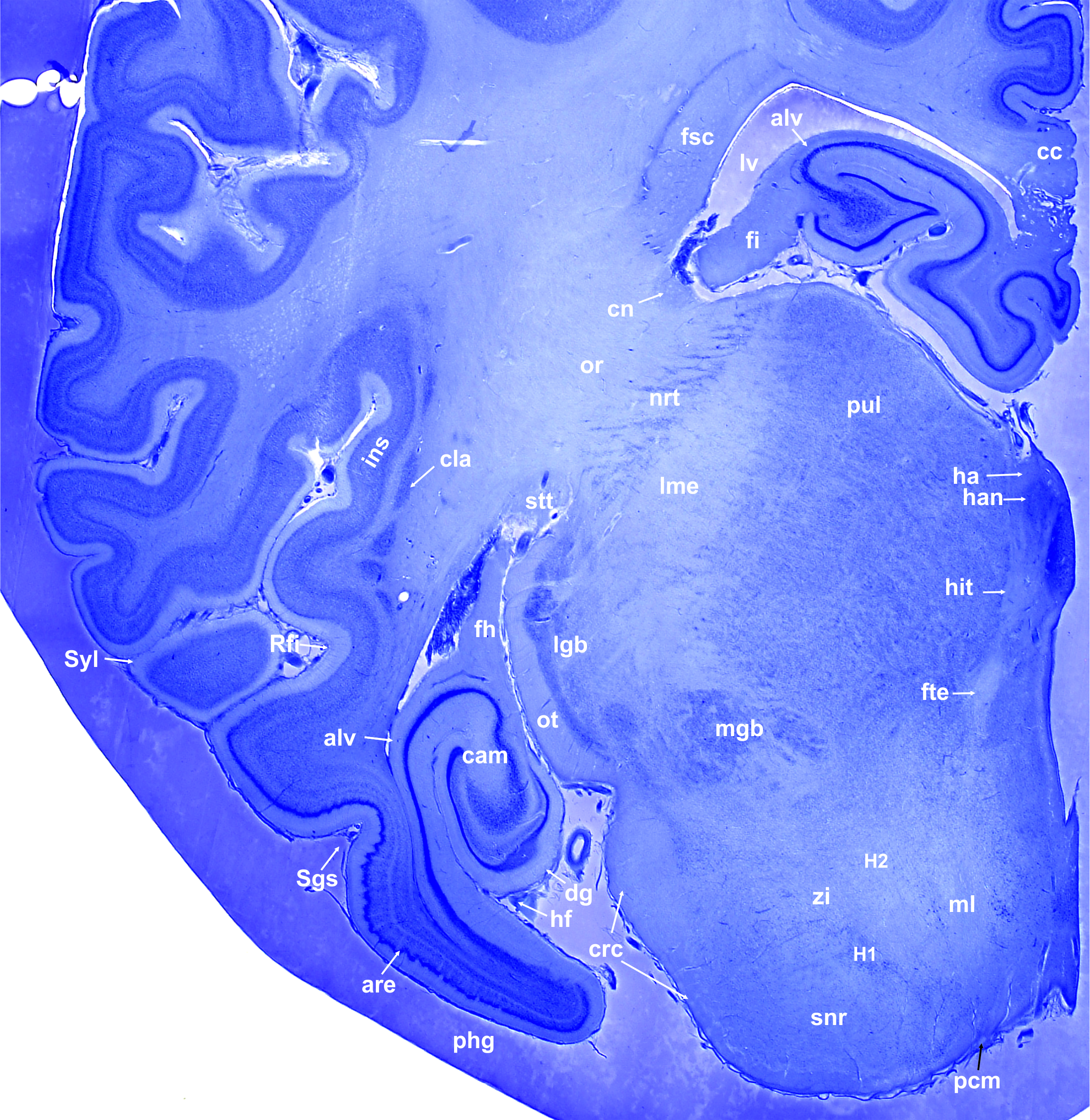

Supplement: S7 Fig — alv: alveus, are: entorhinal area, cam: cornu amonis, cc: corpus calosum, cla: claustrum, cn: caudate nucleus, crc: cerebral crus, dg: dentate gyrus, fh: fimbria of the hippocampus, fsc: subcallosal fasciculus, H1: fields of Forel 1, H2: Fields of Forel 2, ha: habenula, han: habenular nuclei, hf: hippocampal fissure, hit: habenulo-interpeduncular tract, ins: insular cortex, lgb: lateral geniculate body, lme: external medullary lamina, lv: lateral ventricle, mgb: medial geniculate body, ml: medial lemniscus, nrt: reticular nucleus of the thalamus, or: optic radiation, ot: optic tract, pcm: peduncles of the mammillary body, pul: pulvinar nuclei, Rfi: rhinal fissure, Sgs: sagittal sulcus, snr: substantia nigra, sub: subiculum, stt: terminal stria, Syl: sylvian fissure, zi: zona incerta. (TIF) [file pone.0213814.s008.tif]

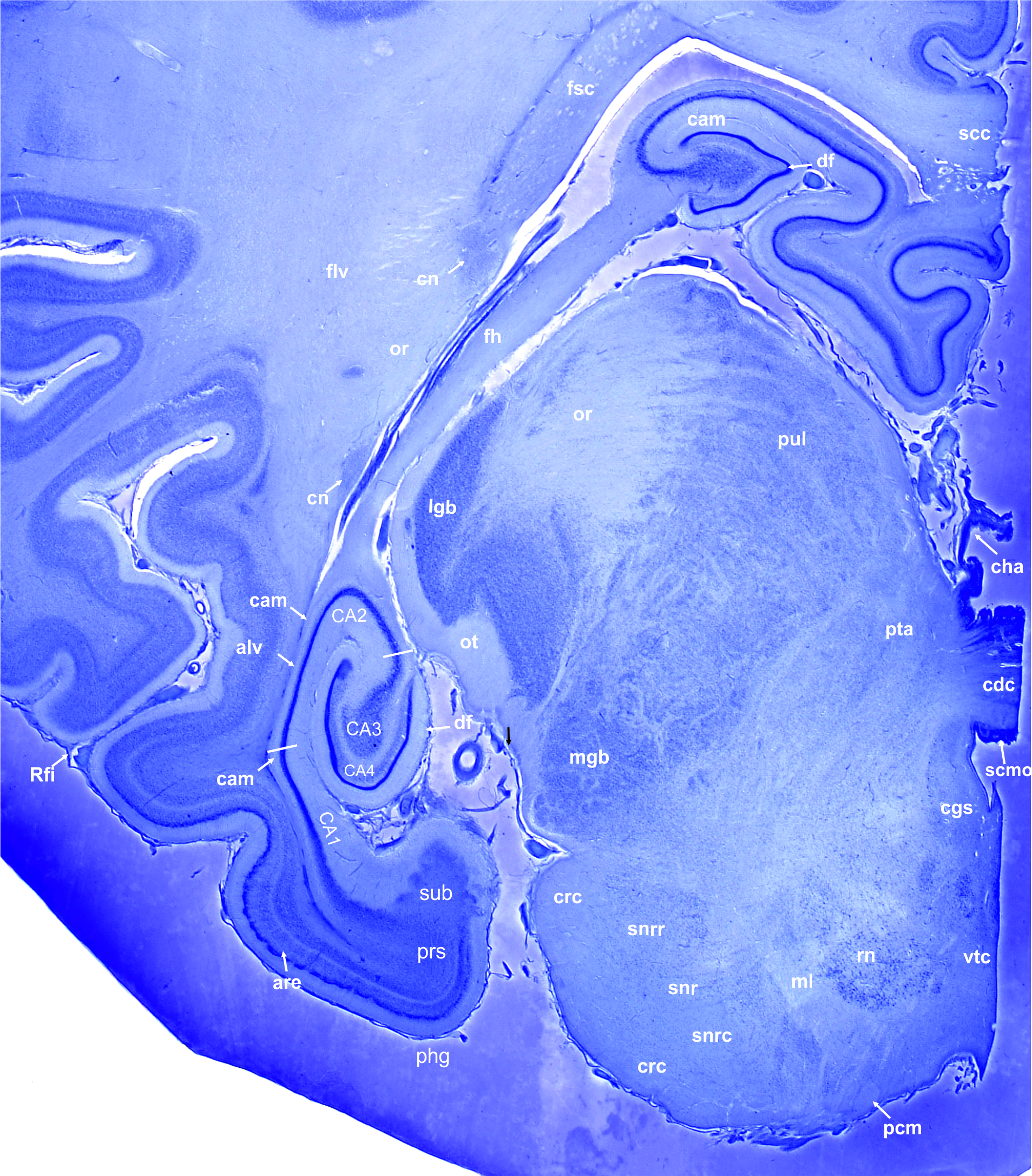

Supplement: S8 Fig — are: entorhinal area, alv: alveus, cam: ammon’s horn, CA1: cornu ammonis field 1, CA2: cornu ammonis field 2, CA3: cornu ammonis field 3; CA4: cornu ammonis field 4, cdc: caudal colliculus, cgs: central grey substance, cha: habenular commissure, cn: caudate nucleus, crc: cerebral crus, df: dentate fascia, fh: fimbria of the hippocampus, flv: ventral longitudinal fasciculus, fsc: subcallosal fasciculus, lgb: lateral geniculate body, mgb: medial geniculate body, ml: medial lemniscus, or: optic radiation, ot: optic tract, pb: pineal body, pcm: peduncles of the mammillary body, prs: presubiculum, pta: pretectal area, pul: pulvinar nuclei, Rfi: rhinal fissure, rn: red nucleus, scc: splenium of corpus callosum, scmo: subcommissural organ, snrc: pars compacta of the substantia nigra, snrr: pars reticularis of the substantia nigra, sub: subiculum, vtc: ventral tegmental commissure. (TIF) [file pone.0213814.s009.tif]

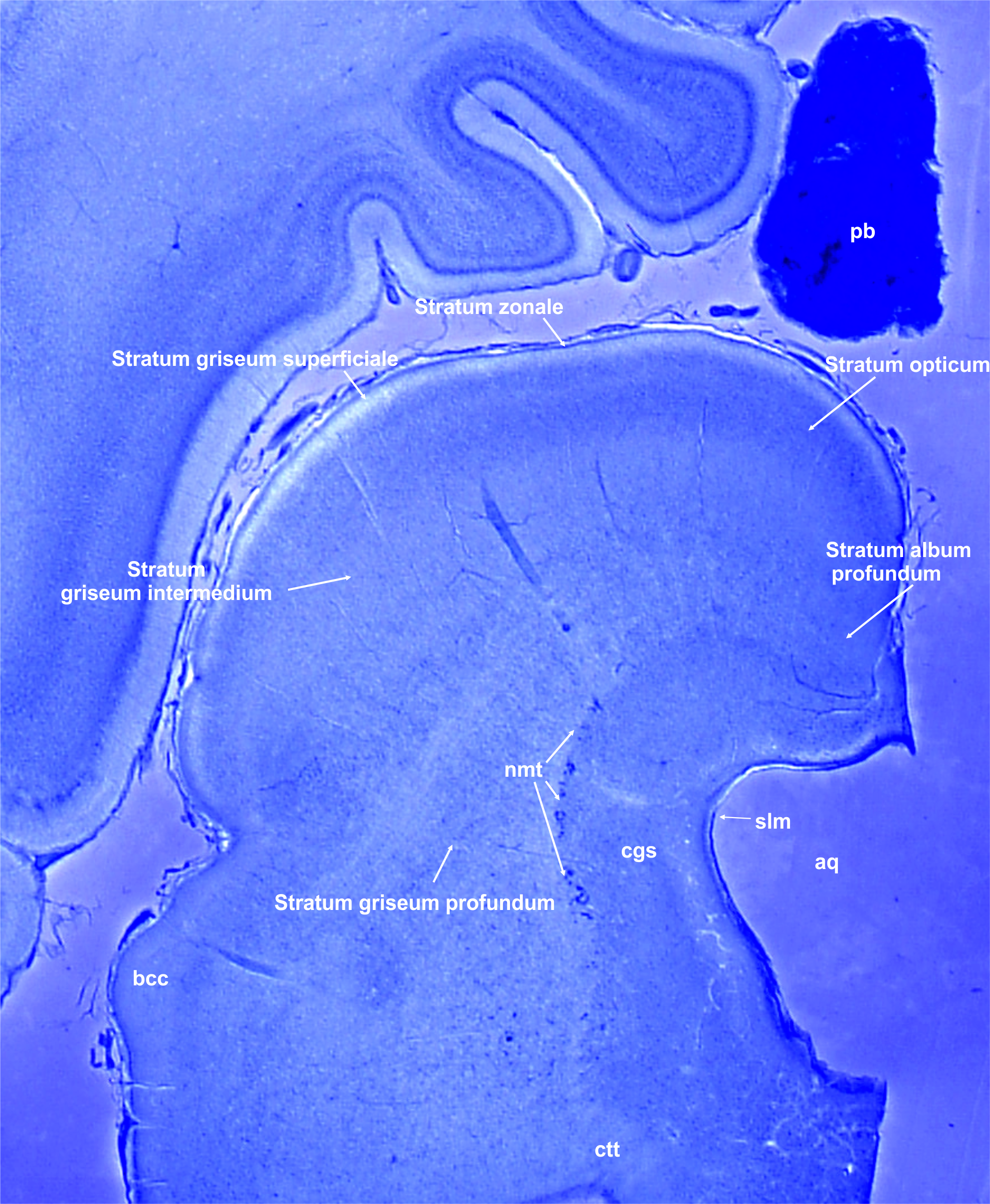

Supplement: S9 Fig — aq: mesencephalic aqueduct, bcc: brachium of the caudal colliculus, cgs: central grey substance, ctt: central tegmental tract, nmt: mesencephalic nucleus of the trigeminal nerve, pb: pineal body, slm: sulcus limitans. (TIF) [file pone.0213814.s010.tif]

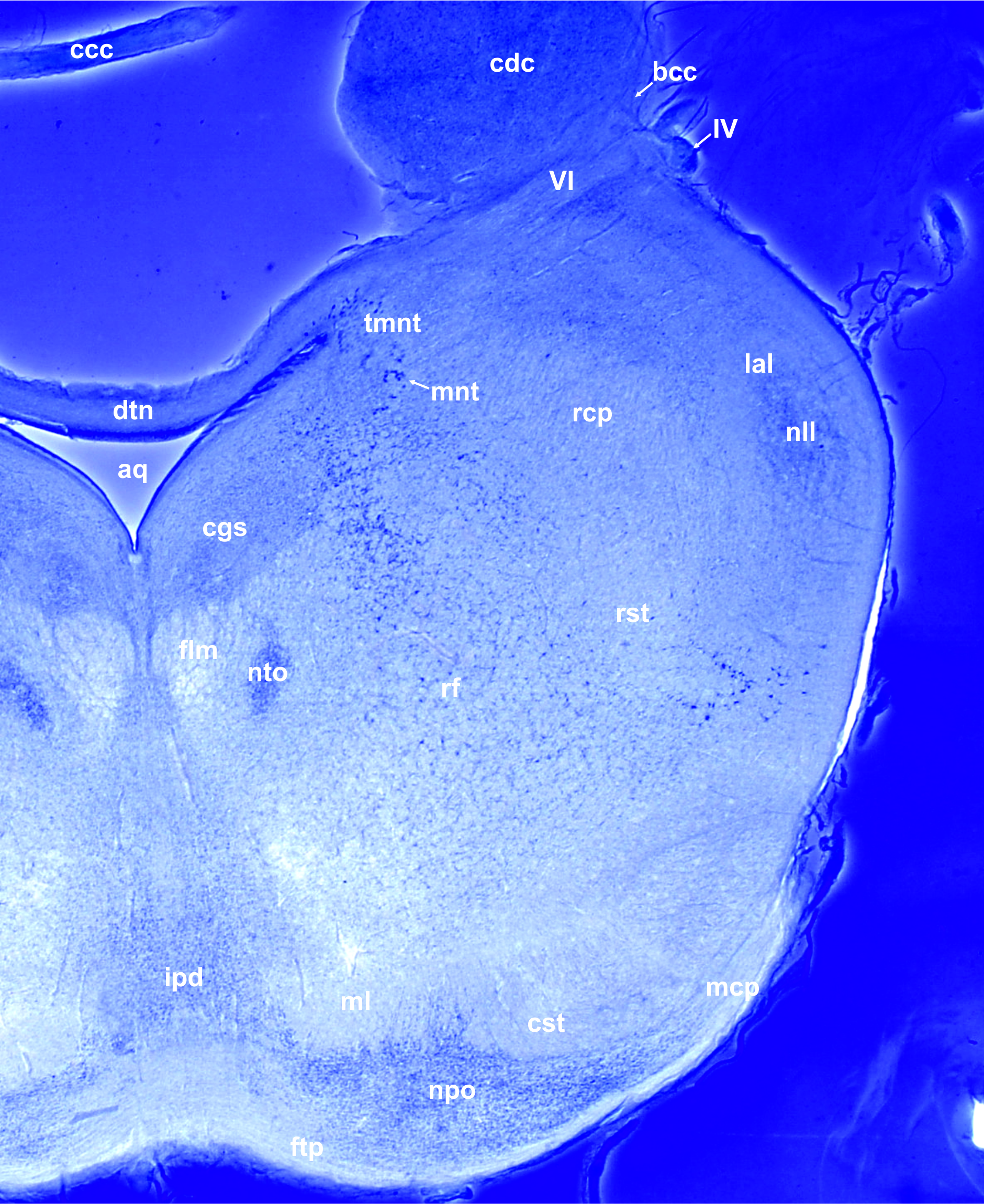

Supplement: S10 Fig — aq: mesencephalic aqueduct, bcc: brachium of the caudal colliculus, cdc: caudal colliculus, cst: corticospinal tract, dtn: decussation of the trochlear nerve, flm: medial longitudinal fasciculus, ftp: transverse fibres of the pons, ipd: interpeduncular nucleus, lal: lateral lemniscus, mcp: medial cerebellar peduncle, ml: medial lemniscus, nII: nucleus of lateral lemniscus, npo: nuclei of the pons, nto: nucleus of trochlear nerves, rcp: rostral cerebellar peduncle, rf: reticular formation, rst: rubrospinal tract, tmnt: mesencephalic tract of the trigeminal nerve, VI: abducence nerve. (TIF) [file pone.0213814.s011.tif]

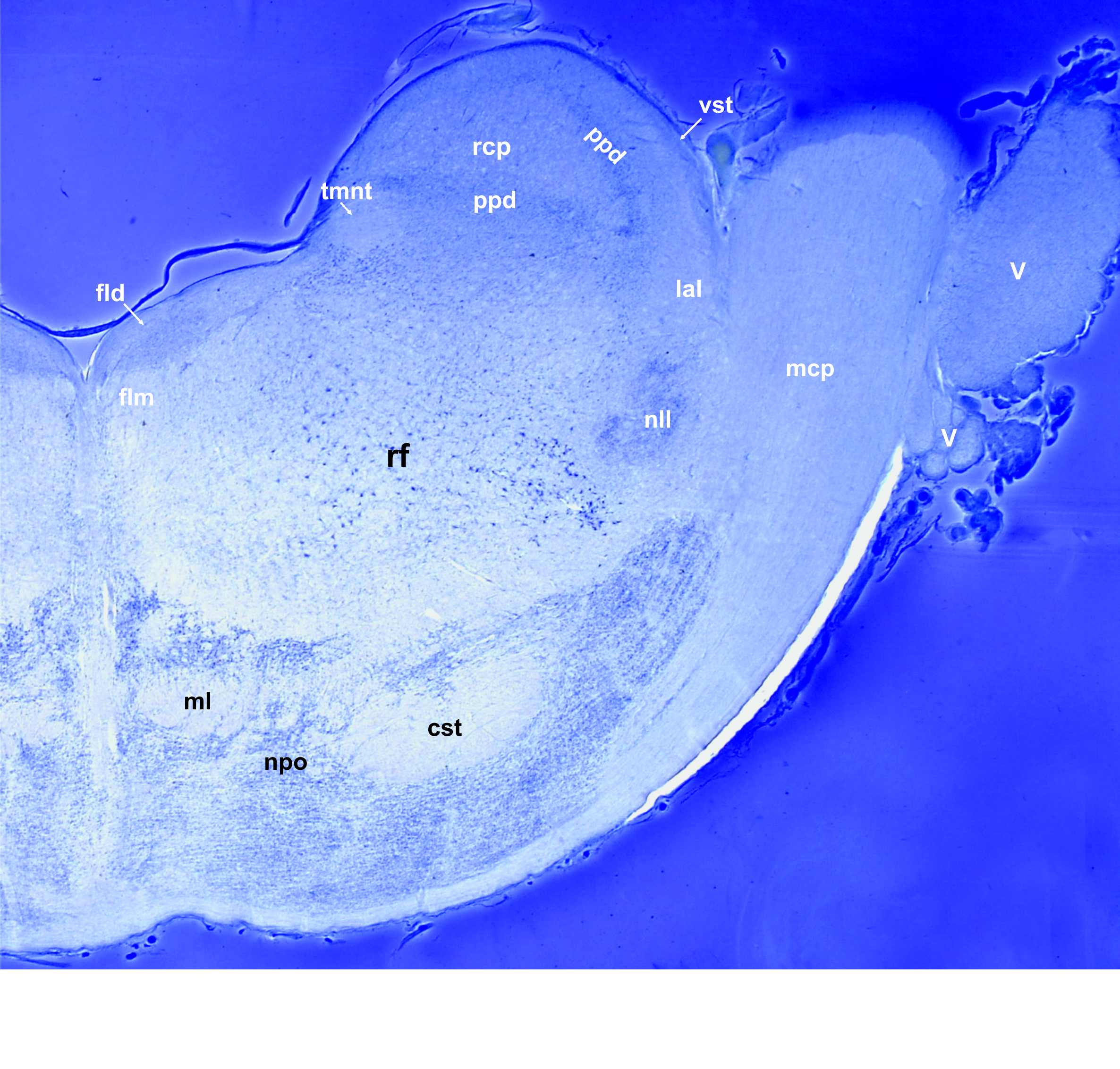

Supplement: S11 Fig — cst: corticospinal tract, fld: dorsal longitudinal fasciculus, flm: medial longitudinal fasciculus, IaI: lateral lemniscus, mcp: medial cerebellar peduncle, ml: medial lemniscus, nII: nucleus of lateral lemniscus, npo: nuclei of the pons, ppd: parapeduncular nuclei, rcp: rostral cerebellar peduncle, rf: reticular formation, vst: vestibulospinal tract, V: trigeminal nerve. (TIF) [file pone.0213814.s012.tif]

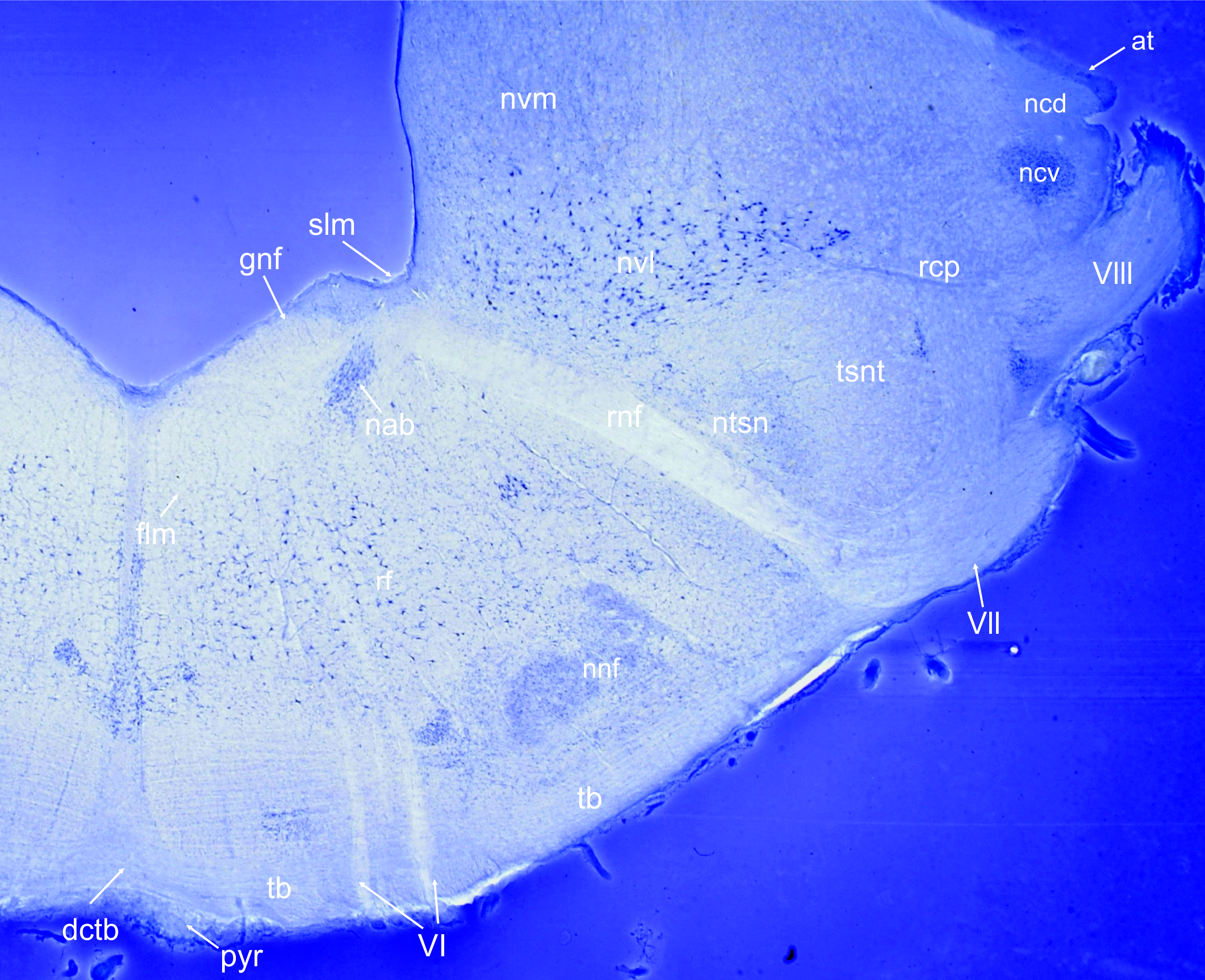

Supplement: S12 Fig — dctb: decussation of the trapezoid body, flm: medial longitudinal fasciculus, gnf: genu of the facial nerve, nab: nucleus of the abducent nerve, ncd: dorsal cochlear nucleus, ncv: ventral cochlear nucleus, ndct: superior olivary nucleus, ntsn: nucleus of the spinal tract of the trigeminal nerve, nvl: lateral vestibular nuclei, pyr: pyramidal tract, rcp: rostral cerebellar peduncle, rf: reticular formation, rnf: radix of the facial nerve, rst: rubro-spinal tract, slm: sulcus limitans, tb: trapezoid body, tsnt: spinal tract of the trigeminal nerve, VI: roots of the abducence nerve, VII: facial nerve, VIII: vestibulocochleal nerve. (TIF) [file pone.0213814.s013.tif]

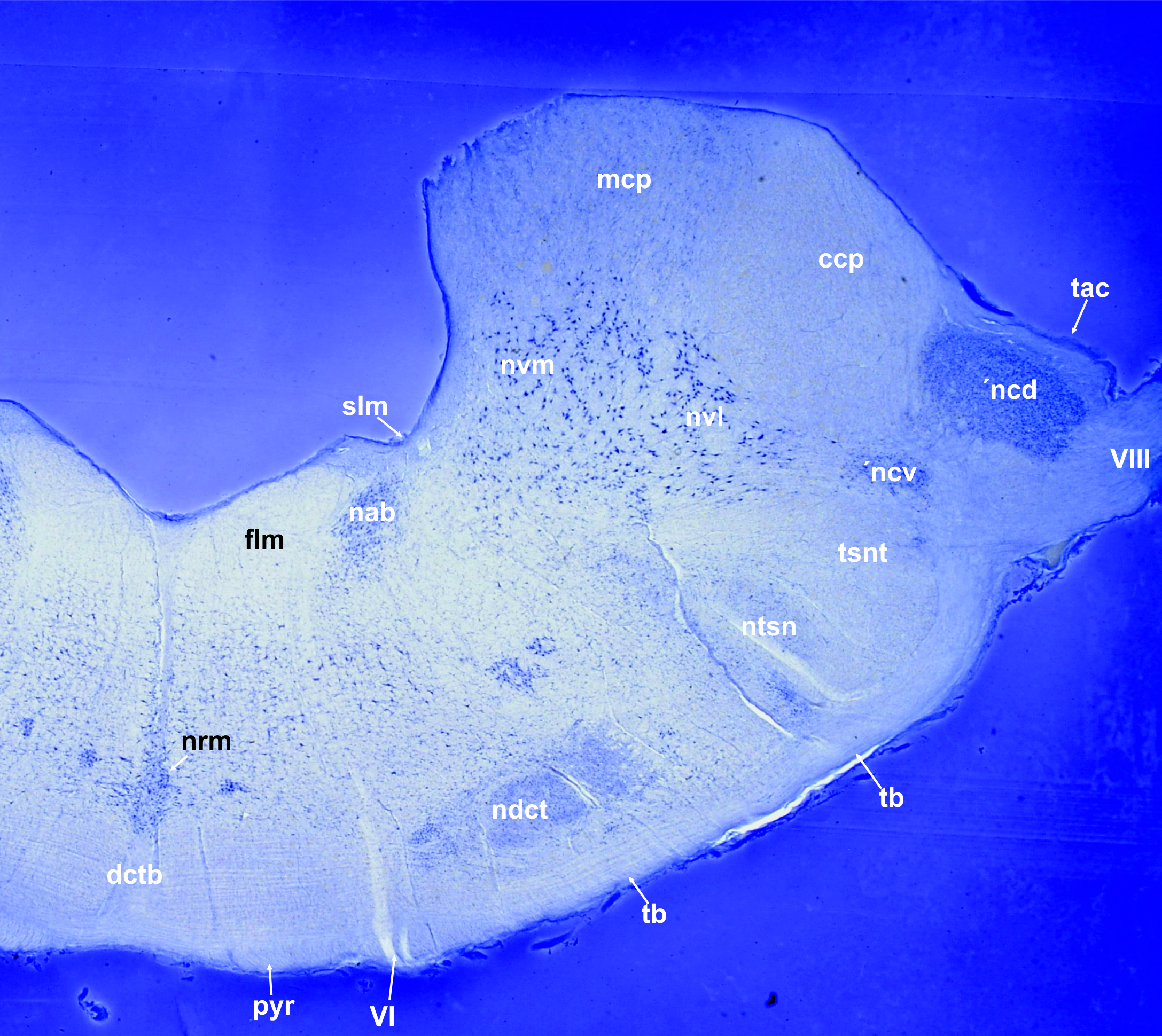

Supplement: S13 Fig — ccp: caudal cerebellar peduncle, dctb: decussation of the fibres of trapezoid body, flm: medial longitudinal fasciculus, li: lingula of the vermis, mcp: medial cerebellar peduncle, ncd: dorsal cochlear nucleus, ncv: ventral cochlear nucleus, ndct: superior olivary nucleus, ntsn: nucleus of the spinal tract of the trigeminal nerve, nvl: lateral vestibular nuclei, nvm: medial vestibular nucleus, pyr: pyramidal tract, rst: rubrospinal tract, slm: sulcus limitans, tac: acoustic tubercle, tb: trapezoid body, tsnt: spinal tract of the trigeminal nerve, VI: roots of the abducence nerve, VIII: vestibulocochleal nerve. (TIF) [file pone.0213814.s014.tif]

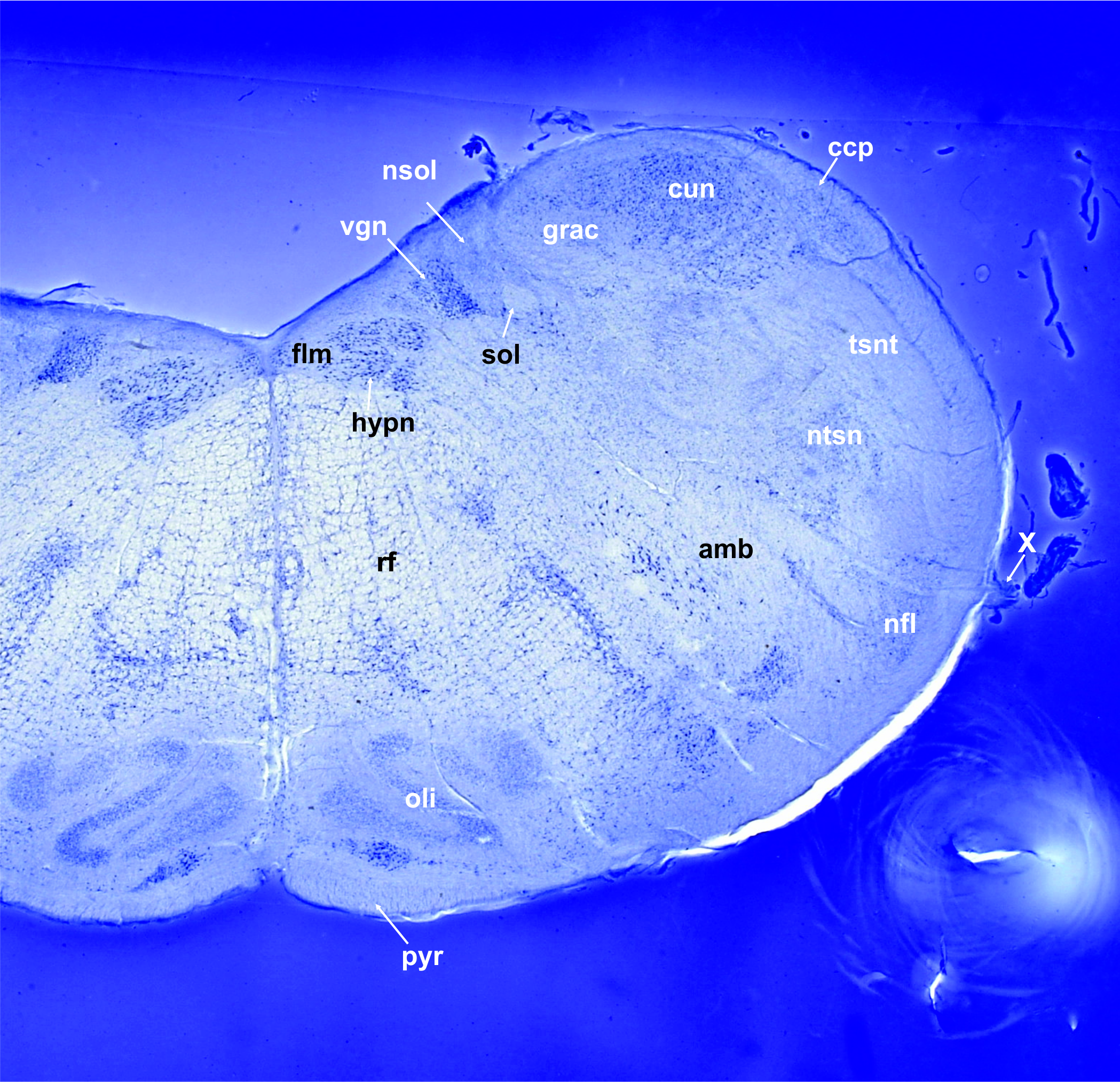

Supplement: S14 Fig — amb: ambiguus nucleus, ccp: caudal cerebellar peduncle, cun: cunetae nucleus, flm: medial longitudinal fasciculus, hypn: nucleus of the hypoglossal nerve, nfl: nucleus of the lateral fascicle, ntsn: nucleus of the spinal tract of the trigeminal nerve, oli: olivary nucleus, pyr: pyramidal tract, sol: nucleus of the solitary tract, soln: nucleus of the solitary trasct, tsnt: spinal tract of the trigeminal nerve, vagn: nucleus of the vagus nerve, X: vagus nerve. (TIF) [file pone.0213814.s015.tif]

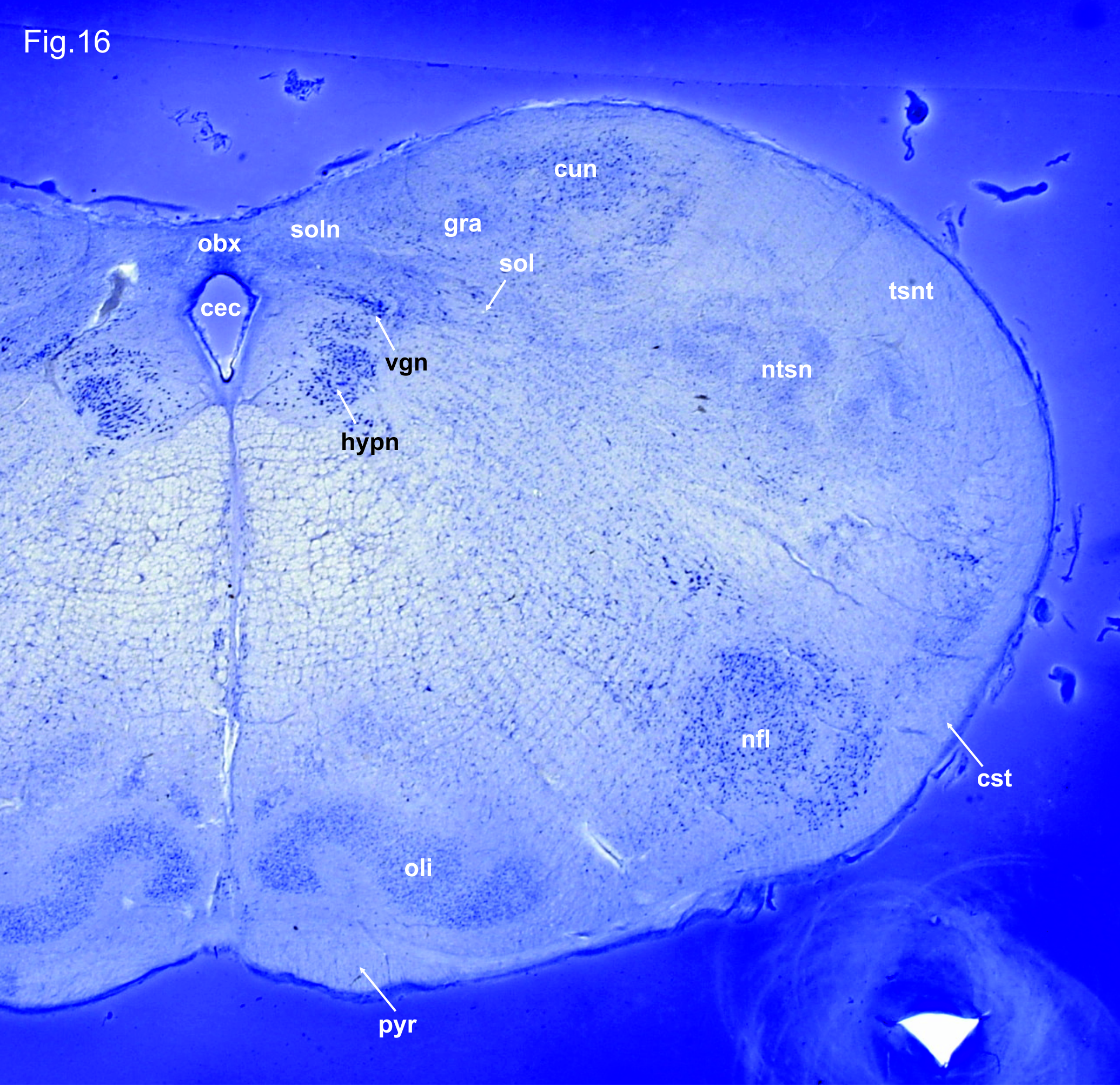

Supplement: S15 Fig — cec: central canal, cst: corticospinal tract, cun: cuneate nucleus, gra: gracile nucleus, hypn: nucleus of the hypoglossal nerve, nfl: nucleus of the lateral fascicle, ntsn: nucleus of the spinal tract of the trigeminal nerve, obx: obex, oli: olivary nucleus, pyr: pyramidal tract, soln: nucleus of the solitary tract, sol: solitary tract, tsnt: spinal tract of the trigeminal nerve, vgn: nucleus of the vagus nerve. (TIF) [file pone.0213814.s016.tif]
